# Spontaneous membrane protrusion and cell morphogenesis via self-propelled actin filaments

**DOI:** 10.1038/s44319-026-00804-6

**Published:** 2026-06-25

**Authors:** Kio Yagami, Takunori Minegishi, Kentarou Baba, Shinji Misu, Hiroko Katsuno-Kambe, Kazunori Okano, Yuichi Sakumura, Yoichiroh Hosokawa, Naoyuki Inagaki

**Affiliations:** 1 Laboratory of Systems Neurobiology and Medicine, Division of Biological Science, Nara Institute of Science and Technology, Ikoma, Japan; 2 Laboratory of Multicellular Network, Division of Biological Science, Nara Institute of Science and Technology, Ikoma, Japan; 3 Bio-Process Engineering Laboratory, Division of Materials Science, Nara Institute of Science and Technology, Ikoma, Japan; 4 Laboratory of Data-driven Biology, Division of Biological Science, Nara Institute of Science and Technology, Ikoma, Japan; 5 Laboratory of Mechano-Systems Biology and Medicine, Strategic Initiative for Research and Innovation, Nara Institute of Science and Technology, Ikoma, Japan

**Keywords:** Cell Adhesion, Polarity & Cytoskeleton, Computational Biology

## Abstract

Cells frequently undergo spontaneous morphogenesis, yet the underlying mechanisms remain incompletely understood. While actin filaments are central to cell morphogenesis and are typically regulated by biochemical signaling, cells can form protrusions even without clear external cues, suggesting the existence of intrinsic mechanisms. Here, we report that actin filament assemblies undergo directional movement driven by their directional polymerization and disassembly. These filament assemblies move as discrete “particles” and exhibit random yet directional motion. Since this motion resembles that of self-propelled “particles” rather than the previously reported reaction-diffusion “waves”, we have termed them Self-propelled Treadmilling Actin filaments (SpTAs). SpTA arrival at the cell periphery drives membrane protrusion by orienting their polymerizing ends outwards. Furthermore, SpTAs spontaneously accumulate at cell protrusions, guided by membrane curvature. This SpTA accumulation, further boosts the growth and expansion of protrusions, driving cellular polarization for migration. Our findings establish that the assembly of actin filaments as a novel class of biological active particle, and provides new insight into how molecular-scale motion orchestrates complex higher-order organization.

## Synopsis

Self-propelled Treadmilling Actin filaments (SpTAs) undergo intracellular movement in a manner similar to that of self-propelled particles studied in active matter physics. SpTA functions as an intrinsic driver of membrane protrusion and cell shaping.

- SpTA undergoes directional yet random intracellular movement via actin polymerization and disassembly.
- SpTA drives membrane protrusion and accumulates spontaneously at cell protrusions.
- SpTA mediates spontaneous membrane protrusion and cell morphogenesis.

## Abstract (web summary)

Self-propelled Treadmilling Actin filaments (SpTAs) undergo intracellular movement in a manner similar to that of self-propelled particles studied in active matter physics. SpTA functions as an intrinsic driver of membrane protrusion and cell shaping.

## Graphical abstract

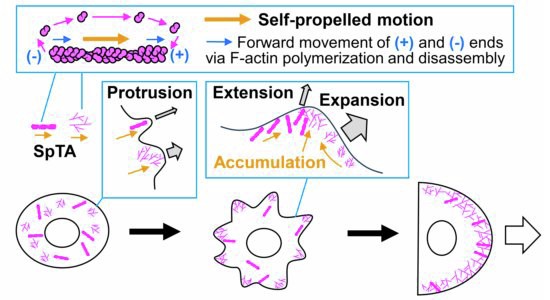

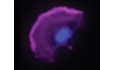

## Introduction

Biological systems exhibit complex shapes that undergo dynamic changes, crucial processes for their function. Among these, actin-based protrusions are essential for cellular morphogenesis and motility (Pollard and Borisy, 2003; Carlier and Shekhar, 2017; Rottner and Schaks, 2019). These structures appear in various forms, such as lamellipodia and filopodia for cell migration (Pollard and Borisy, 2003; Carlier and Shekhar, 2017; Rottner and Schaks, 2019), microvilli to enhance nutrient absorption (Gov, 2006; Meenderink et al, 2019), and phagocytic cups to ingest microbes (Rottner and Schaks, 2019). Their formation has conventionally been explained by local polymerization and disassembly of actin filaments (F-actins), orchestrated by biochemical signaling that activates key regulator proteins, such as the Arp2/3 complex, formins, Ena/VASP family members and ADF/cofilin (Pollard and Borisy, 2003; Bernstein and Bamburg, 2010; Vitriol et al, 2013; Wang et al, 2015; Carlier and Shekhar, 2017; Rottner and Schaks, 2019; Goode et al, 2023). Notably, cells exhibit the remarkable ability to spontaneously extend protrusions, even in the absence of explicit signaling cues. This suggests the existence of an intrinsic mechanism that drives these structural transformations beyond localized signaling.

F-actins undergo wave-like propagation, termed actin “waves”, in various cell types (Carlsson, 2010; Allard and Mogilner, 2013; Inagaki and Katsuno, 2017; Beta et al, 2023). These phenomena have primarily been modeled by the reaction-diffusion or Turing-type frameworks, involving positive and negative feedback interactions (Carlsson, 2010; Allard and Mogilner, 2013; Inagaki and Katsuno, 2017; Beta et al, 2023). However, the underlying molecular basis and physical principles that validate these propagation models remain unclear (Beta et al, 2023). In parallel, the field of active matter physics has seen a surge of interest in self-propelled particles (also known as self-propelled Brownian particles, active particles or microswimmers) (Ebbens and Howse, 2010; Kaiser et al, 2012; Elgeti et al, 2015; Bechinger et al, 2016). They can take energy from their environment and convert it into directed motion to propel themselves, and their trajectories involve random fluctuations. Recent computational simulations and microswimmer studies have shown that these particles, when confined within cell-like vesicles, can spontaneously induce diverse membrane deformations similar to those observed during cellular morphogenesis (Paoluzzi et al, 2016; Li and Ten Wolde, 2019; Vutukuri et al, 2020; Boudet et al, 2021).

F-actins have the inherent property to undergo energy-driven directional polymerization-disassembly cycle, known as treadmilling (Pollard and Borisy, 2003; Carlier and Shekhar, 2017; Oosterheert et al, 2022; Goode et al, 2023). In this process, ATP-bound actin monomers polymerize at the plus ends of F-actins. This is followed by ATP hydrolysis within the filaments, which in turn leads to F-actin disassembly from the minus ends as ADP-bound actin molecules destabilize F-actins. Thus, F-actins undergo treadmilling by consuming ATP. Actin-binding proteins further control the organization and polymerization/disassembly of the cellular F-actin assemblies that constitute protrusions (Carlier and Shekhar, 2017; Rottner and Schaks, 2019). Here we show that F-actin assemblies, which we term Self-propelled Treadmilling Actin filaments (SpTAs), emerge widely in cells and translocate by directional polymerization and disassembly. Crucially, unlike to the continuous propagation of diffusion-reaction based actin wave, SpTAs exhibit random change in their direction of movement and accumulate at protrusive plasma membrane regions in a manner similar to the self-propelled particles (Kaiser et al, 2012). Perturbing SpTA movement impairs the spontaneous cell polarization essential for migration. We propose that SpTAs broaden our understanding of intracellular actin dynamics and shed light on previously unresolved aspects of cellular behavior, including spontaneous membrane protrusion and cell morphogenesis.

## Results

### F-actin assemblies emerge widely in cells and move in random directions

**Figure 1:**
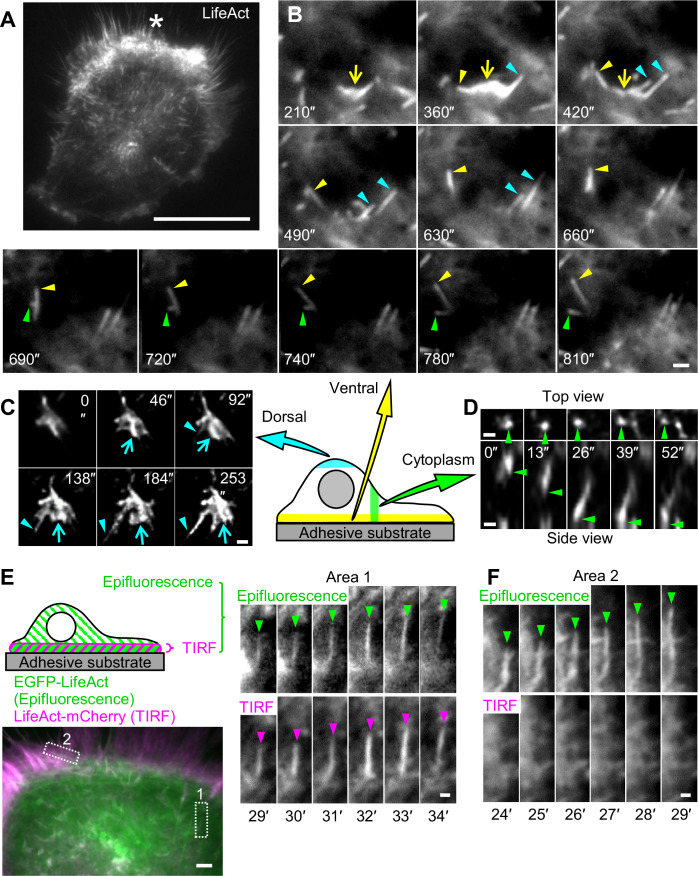
F-actin assemblies emerge widely in cells and move forward. (**A**) A fluorescence image of a U251 cell expressing EGFP-LifeAct obtained by TIRF microscopy. Asterisk indicates cellular leading edge. See Movie EV1. Scale bar, 20 µm. (**B**) Fluorescence time-lapse images of linear F-actin bundles (arrowheads) and F-actin meshworks (arrows) in U251 cells expressing LifeAct-mCherry obtained by TIRF microscopy. Linear F-actin bundles frequently changed their direction of translocation (yellow arrowheads): some emerged and separated (yellow and cyan arrowheads) from F-actin meshworks (arrows) or branched off from linear F-actin bundles (green arrowheads). See Movie EV1. Scale bar, 2 µm. (**C**, **D**) Fluorescence time-lapse images of F-actin assemblies on the dorsal membrane (blue arrowheads and arrows, **C**) and linear F-actin assemblies in cytoplasm (green arrowheads, **D**) in U251 cells expressing LifeAct-mCherry obtained by 3D imaging with confocal deconvolution microscopy. See Movie EV3. Scale bars, 1 µm. (**E**, **F**) Fluorescence time-lapse images of a U251 cell expressing LifeAct-mCherry (obtained by TIRF microscopy) and EGFP-LifeAct (obtained by epifluorescence microscopy): right panel and (**F**) indicate enlarged time-lapse images in rectangular regions 1 and 2. See Movie EV4. Scale bars, 5 µm (**E**); 1 µm (E enlarged view, **F**). Source data are available online for this figure.

To investigate the intracellular actin dynamics in detail, we analyzed a subline of U251 glioma cells that spontaneously polarize and migrate in the absence of local signaling cues (Appendix Fig. S1A) (Vassilev et al, 2017). These cells actively form two types of protrusions, broad lamellipodia and thin filopodia, at the front (Appendix Fig. S1B). Actin dynamics near the ventral plasma membrane were visualized with the F-actin marker LifeAct using total internal reflection fluorescence (TIRF) microscopy (Fig. 1A). Notably, assemblies of F-actins that form linear bundles or broad meshworks emerged widely near the ventral plasma membrane, moved in random directions, and disappeared (Movie EV1). The majority of them were linear bundles (arrowheads, Fig. 1B; Movie EV1 right) and underwent random yet directional motion (yellow arrowheads, Fig. 1B and Movie EV1, right). The F-actin bundles and meshworks were interchangeable: some F-actin bundles emerged (yellow and cyan arrowheads) from meshworks (arrows) or branched off (green arrowheads) from linear bundles (Fig. 1B; Movie EV1, right). We also observed meshworks (arrows, Appendix Fig. S1C; Movie EV1, right) expanded from linear bundles (arrowheads). Live cell imaging of Lifeact-mCherry and mNeonGreen-fascin revealed that the filopodial constituent fascin is localized in the F-actin bundles (arrowheads, Appendix Fig. S1D). Similar movement of F-actin assemblies were observed in COS7 cells (Appendix Fig. S1E; Movie EV2), suggesting that they are not cell type specific.

3D imaging with confocal deconvolution microscopy revealed F-actin assemblies moving along the dorsal plasma membrane (Fig. 1C) as well as those translocating through the cytoplasm without interacting with the plasma membrane (Fig. 1D; Movie EV3). In addition, a combination of TIRF microscopy, which visualizes the region near the ventral plasma membrane, and epifluorescence microscopy, which visualizes the entire cell thickness, revealed a substantial fraction of F-actin assemblies translocating along the ventral plasma membrane (arrowheads, Fig. 1E; Movie EV4), as well as those moving apart from the adhesive substrate at the leading edge (arrowheads, Fig. 1F; Movie EV4). Thus, these F-actin assemblies emerge and translocate widely within cells, dynamically changing their shape and direction.

### Directional polymerization and disassembly drive F-actin assembly translocation

**Figure 2:**
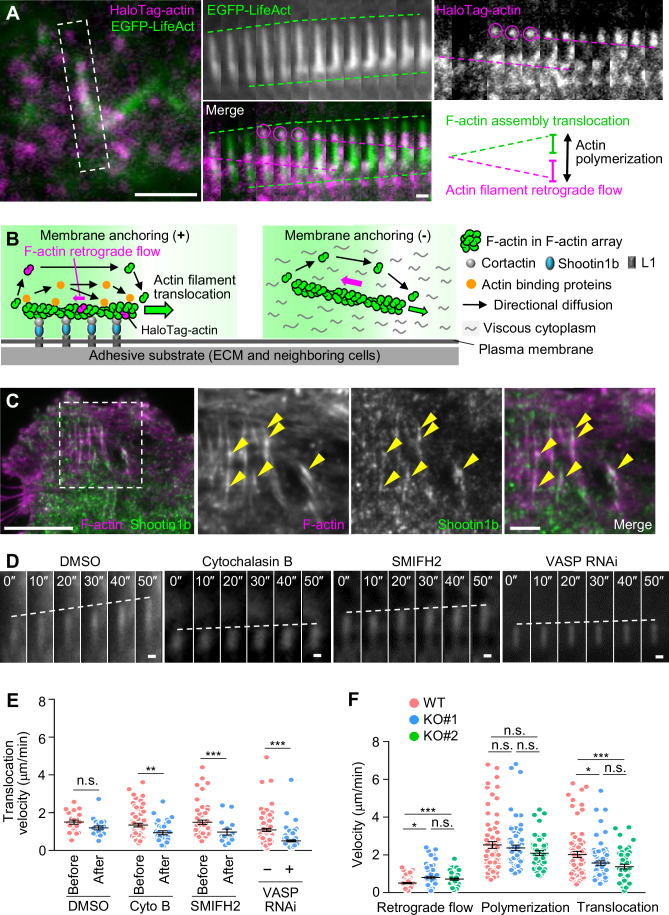
Directional polymerization and disassembly drive F-actin assembly translocation. (**A**) A fluorescent speckle image of HaloTag-actin in a U251 cell obtained by TIRF microscopy; F-actins were also monitored by EGFP-LifeAct. Time-lapse montages of a linear F-actin bundle in the rectangular region at 10-s intervals are shown to the right. F-actin bundle moved forward (green line). HaloTag actin molecules expressed at a low level were incorporated at the front of the F-actin bundle (magenta circles), by polymerization, and underwent retrograde flow (magenta lines). Actin polymerization rate can be calculated as the sum of the forward translocation rate and actin retrograde flow rate (double-headed arrow). See Movie EV5. Scale bars, 5 µm (left); 1 µm (right). (**B**) A diagram showing the translocation mechanisms of the F-actin assembly travelling on the ventral plasma membrane (left). Right panel shows a possible mode of F-actin assembly translocation observed in Fig. 1C,D. For explanations, see text. (**C**) A fluorescence image of a U251 cell stained with anti-shootin1b antibody and Alexa Fluor 594 conjugated phalloidin (for F-actin) obtained by TIRF microscopy. An enlarged view of the area within the rectangle is shown in the right panel. Arrowheads indicate shootin1b colocalization with F-actins. See Appendix Fig. 2E,F. Scale bars, 20 µm (left); 5 µm (right). (**D**) Fluorescence time-lapse images of F-actin bundles in U251 cells after the application of DMSO, 0.05 µM cytochalasin B, and 5 µM SMIFH2, and in a U251 cell expressing VASP knockdown vector. Images were obtained by TIRF microscopy at 10-s intervals. White lines indicate forward translocation of the F-actin bundles. Scale bars, 1 µm. (**E**) Translocation velocities of linear F-actin bundles (white lines, **D**) before and 10–30 mim after the application of DMSO (control, *N* = 3 experiments; *n* = 3 cells; before application, *n* = 34 F-actin bundles; after application, *n* = 32 F-actin bundles), cytochalasin B (*N* = 5 experiments; *n* = 10 cells; before application, *n* = 66 F-actin bundles; after application, *n* = 32 F-actin bundles), SMIFH2 (*N* = 4 experiments; *n* = 8 cells; before application, *n* = 73 F-actin bundles; after application, *n* = 31 F-actin bundles), and in U251 cells expressing control vector (VASP RNAi -, *N* = 3 experiments; *n* = 11 cells, *n* = 90 F-actin bundles) and VASP knockdown vector (VASP RNAi +, *N* = 3 experiments; *n* = 11 cells, *n* = 81 F-actin bundles). *P* = 0.0658 (DMSO); *P *= 0.0142 (cytochalasin B); *P *= 0.00302 (SMIFH); *P* = 3.6 × 10^−13^ (VASP RNAi). Data represent means ± SEM. (**F**) Velocities of F-actin retrograde flow (magenta line, **A**), F-actin polymerization (double-headed arrow, **A**), and translocation (green line, **A**) of linear F-actin bundles in WT and shootin1b KO U251 cells (WT, *N* = 3 experiments; *n* = 11 cells, *n* = 71 F-actin bundles; KO#1, *N* = 3 experiments; *n* = 9 cells, *n* = 69 F-actin bundles; KO#2, *N* = 3 experiments; *n* = 10 cells, *n* = 45 F-actin bundles). Retrograde flow: *P* = 0.0462 (WT vs KO#1); *P* = 0.0022 (WT vs KO#2); *P* = 0.825 (KO#1 vs KO#2). Polymerization: *P* = 0.918 (WT vs KO#1); *P* = 0.249 (WT vs KO#2); *P* = 0.318 (KO#1 vs KO#2). Translocation: *P* = 0.0462 (WT vs KO#1); *P* = 0.0091 (WT vs KO#2); *P* = 0.322 (KO#1 vs KO#2). See also Appendix Figs. S3 and S7. Data represent means ± SEM. ***P < 0.01; **P < 0.02; *P < 0.05; n.s. not significant. Statistical analyses were performed using the two-tailed Mann‒Whitney *U* test (**E**, **F**). Source data are available online for this figure.

To analyze the mechanism that translocates F-actin assemblies, we monitored their subunit, actin molecules, by speckle imaging of HaloTag-actin (Minegishi et al, 2021). F-actin assemblies moving forward were labelled by EGFP-LifeAct (green lines, Fig. 2A, Appendix Fig. S2A and B, Movie EV5). HaloTag actin molecules expressed at a low level were incorporated at the front of the F-actin assemblies, by polymerization (magenta circles, Fig. 2A, Appendix Fig. S2A), and underwent retrograde flow (magenta lines). Similar retrograde movement of actin molecules was observed in F-actin assemblies advancing without interaction with the plasma membrane (Appendix Fig. S2C), indicating that the F-actins in the forward-advancing assemblies undergo retrograde flow, accompanied by the polymerization at the front and disassembly at the rear (lower panel, Appendix Fig. S2A).

We previously reported F-actin assemblies that translocate one-dimensionally along neuronal axons through their directional polymerization and disassembly (Katsuno et al, 2015). Their front and rear ends polymerize and disassemble, respectively (black arrows, Fig. 2B left) (Katsuno et al, 2015; Inagaki and Katsuno, 2017). In addition, they are weakly anchored to the plasma membrane and adhesive substrate, by the linker molecules shootin1a and cortactin and the cell adhesion molecule L1 (Katsuno et al, 2015; Kubo et al, 2015). As this anchoring impedes the retrograde flow (slippage) of F-actins (magenta arrow, Fig. 2B, left), the actin polymerization/disassembly rate exceeds the retrograde flow rate, resulting in the forward movement of the front and rear ends of F-actins (green arrow). During the forward movement, local concentrations of actin subunits and actin-binding proteins decrease at the front by polymerization and increase at the rear by disassembly, resulting in their diffusion along the local concentration gradients (black arrows, Fig. 1B, left). Accompanied by this forward diffusion, F-actin assemblies translocate actin and actin-binding proteins along axons (Flynn et al, 2009; Katsuno et al, 2015).

To examine whether the observed F-actin assemblies translocate via directional polymerization and disassembly, we further analyzed their motion under drug treatments and molecular knockdown. However, the shape of the F-actin meshworks changes dynamically, which makes analyzing their velocity difficult. Additionally, the F-actin bundles comprise the majority of the F-actin assemblies, and the F-actin bundles and meshworks are interchangeable. Therefore, we focused on the analyses of the F-actin bundles. Immunoblot analyses showed that U251 cells express, shootin1b, cortactin and L1 (Appendix Fig. S2D); shootin1b is a splicing variant of shootin1a that also mediates the actin-L1 linkage (Minegishi et al, 2018). Shootin1b co-localized with the F-actin bundles (arrowheads, Fig. 2C; Appendix Fig. S2E,F). Partial inhibition of actin polymerization by cytochalasin B at a low concentration (0.05 µM) (Katsuno et al, 2015) decreased the velocity of F-actin bundle translocation (Fig. 2D,E). A relatively non-specific formin inhibitor SMIFH2 (5 µM) (Nishimura et al, 2021) and VASP knockdown also reduced the translocation velocity (Fig. 2D,E; Appendix Fig. S3A), indicating that their translocation is driven by actin polymerization. Interestingly, the Arp2/3 complex inhibitor CK666 (100 µM) (Nolen et al, 2009) also reduced the translocation velocity (Appendix Fig. S3B, see Appendix discussion). The length of the translocating F-actin bundles was also reduced by these inhibitors and VASP knockdown (Appendix Fig. S3C).

Furthermore, shootin1b knockout (KO) (Appendix Fig. S3D) increased the retrograde flow velocity of F-actins in the F-actin assemblies, without affecting the polymerization rate (Fig. 2F), indicating that shootin1b impedes the retrograde flow of F-actins by anchoring them to the plasma membrane (for explanation see also Appendix Fig. S3E). Consistently, shootin1b KO reduced the velocity of F-actin assembly translocation (Fig. 2F). Together, these results indicate that directional polymerization and disassembly drives translocation of the F-actin assemblies and that shootin1b promotes their translocation by impeding their retrograde flow (Fig. 2B, left).

We have also observed F-actin assemblies translocating apart from the adhesive substrate (Fig. 1C,F), as well as in the cytoplasm without interacting with the plasma membrane (Fig. 1D). Moreover, the inhibition of F-actin anchorage to the plasma membrane by shootin1b KO did not completely prevent their translocation (Fig. 2F). The cytoplasm represents a viscous and crowded intracellular environment containing proteins, macromolecules, and cytoskeletal components (Cameron et al, 2004; Kalwarczyk et al, 2011). We consider that the F-actin assemblies can move forward without anchoring to the membrane when retrograde F-actin flow is mechanically obstructed in the viscous and crowded intracellular environment (Fig. 2B, right).

### F-actin assemblies move as self-propelled particles and drive membrane protrusions

**Figure 3:**
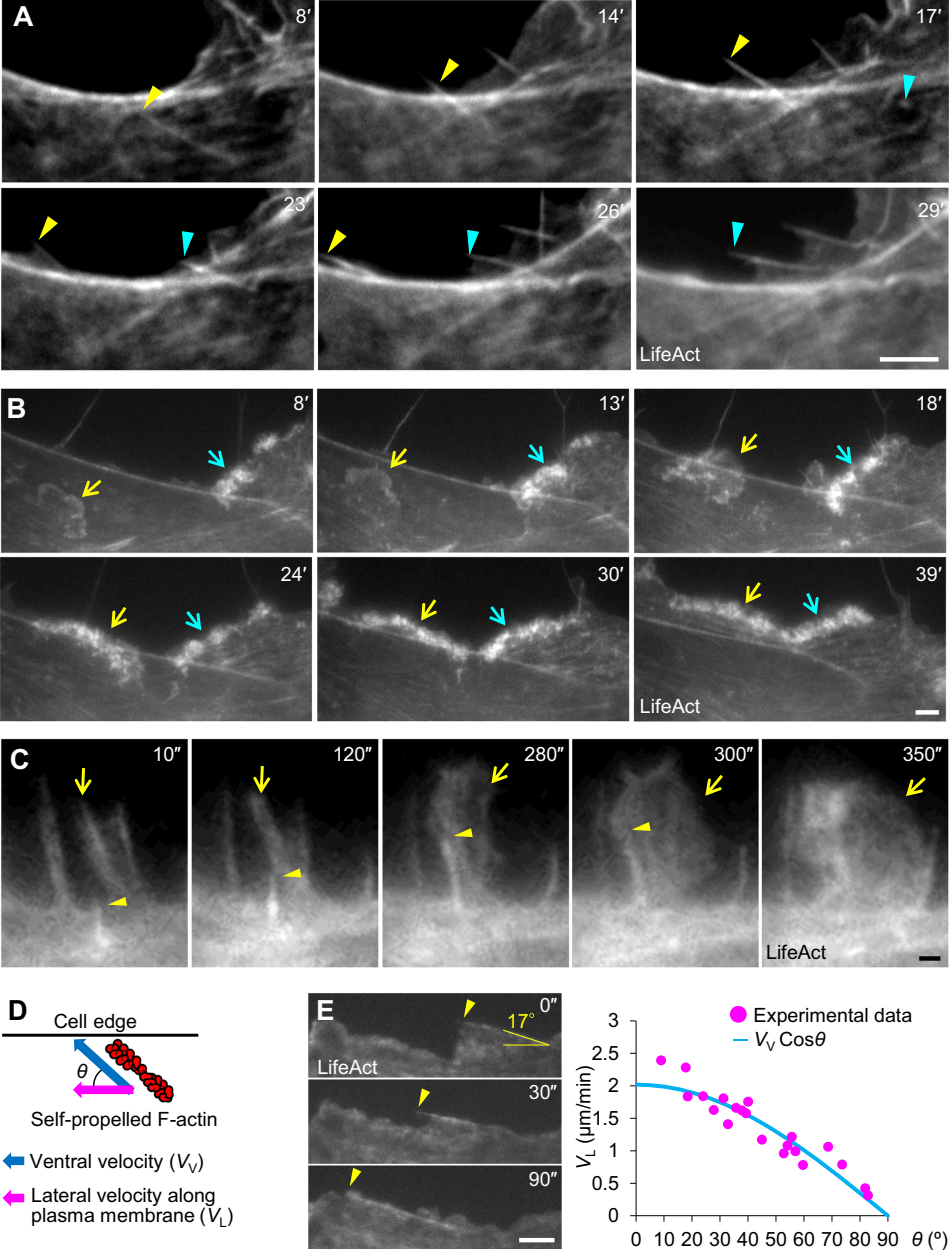
F-actin assembly arrival at the cell periphery drives membrane protrusion. (**A**, **B**) Fluorescence time-lapse images of linear F-actin bundles (**A**) and F-actin meshworks (**B**) arrived at the cell periphery of U251 cells expressing EGFP-LifeAct obtained by epifluorescence microscopy (**A**) and expressing LifeAct-mCherry obtained by TIRF microscopy (**B**). They pushed the plasma membrane, resulting in the formation of filopodia (arrowheads) and lamellipodium (yellow arrows), and continued to move laterally along the membrane. The latter merged with the pre-existing lamellipodium (blue arrows). See Movies EV6 and EV7. Scale bars, 5 µm. (**C**) Fluorescence time-lapse images of a linear F-actin bundle (arrowheads) which entered into a pre-existing lamellipodium (arrows) in U251 cells expressing EGFP-LifeAct obtained by TIRF microscopy. Its entry into lamellipodia resulted in local F-actin accumulation and lamellipodial extension (arrows). See Movie EV8 (left). Scale bar, 1 µm. (**D**) A diagram describing the relationship between the velocities of lateral movement (*V*_L_) and ventral movement (*V*_V_) of linear F-actin bundles. If F-actin bundles move laterally along the membrane driven by directional actin polymerization and disassembly, *V*_L_ is equal to *V*_V_ cos*θ*, where *θ* is the angle of the polymerizing F-actins with respect to the membrane. (**E**) Fluorescence time-lapse images of a linear F-actin bundle travelling along the lateral membrane (arrowheads) of a U251 cell expressing LifeAct-mCherry obtained by epifluorescence microscopy. See Movie EV9. The right graph shows *V*_L_ and *θ* obtained by the time-lapse imaging; *V*_L_ closely matched *V*_V_cos*θ* (*N* = 3 experiments; *n* = 10 cells, *n* = 21 F-actin bundles). Scale bar, 5 µm. Source data are available online for this figure.

When the advancing F-actin bundles and meshworks arrived at the cell periphery, they pushed the plasma membrane to form filopodia (arrowheads, Fig. 3A; Movie EV6) and lamellipodia (yellow arrows, Fig. 3B; Movie EV7), respectively. We also observed F-actin bundles entering pre-existing lamellipodia (arrowheads, Fig. 3C; Movie EV8, left) and filopodia (Movie EV8, right). Their entrance in the lamellipodia resulted in local F-actin accumulation and lamellipodial expansion (arrows, Fig. 3C; Movie EV8, left). Some of them continued to move laterally along the membrane (Fig. 3A,B). We also noted F-actin meshworks (yellow arrows, Fig. 3B; Movie EV7) that merged with the pre-existing lamellipodia (cyan arrows) through their lateral movement. These data suggest that the F-actin bundles and meshworks are equivalent to the F-actin assemblies that constitute filopodia and lamellipodia, respectively.

If so, the lateral movements of filopodia and lamellipodia (Fig. 3A,B) would also be propelled by directional polymerization and disassembly. Therefore, their velocity (magenta arrow, Fig. 3D) would depend on the angle *θ* of their polymerizing F-actins with respect to the membrane, as well as the velocity of the advancing F-actin assemblies prior to arriving at the membrane (blue arrow). To examine this possibility, we focused on the motion of the linear F-actin bundles because they are the majority of advancing F-actin assemblies and because it is difficult to observe individual F-actins in F-actin meshworks (Movie EV1). Time-lapse measurements of both the angle *θ* and the velocity of the filopodial lateral movement (*V*_L_) (Fig. 3E; Movie EV9) demonstrated that the velocity closely matched the cosine *θ* for the average velocity of the ventrally translocating F-actin bundles (*V*_V_) (2.0 ± 0.2 µm/min, Figs. 2F and 3E). Taken together, these data indicate that the advancing F-actin assemblies and those in filopodia and lamellipodia are equivalent structures propelled by directional polymerization and disassembly.

Importantly, the F-actin assemblies move as discrete “particles” and do not propagate uniformly as “waves”. In addition, they randomly change the direction of movement and their arrival at the cell periphery results in local membrane protrusion, similar to the self-propelled particles (Kaiser et al, 2012). Thus, we refer to these actin assemblies as Self-propelled Treadmilling Actin filaments (SpTAs). Taking into account that the F-actin bundles and meshworks are equivalent to the F-actin assemblies in filopodia and lamellipodia, we refer to them as filopodium-type SpTAs and lamellipodium-type SpTAs, respectively. Namely, filopodium-type SpTAs are self-propelled linear F-actin bundles (arrowheads, Fig. 1B; Movie EV1, right), while lamellipodium-type SpTAs are self-propelled F-actin meshworks (arrows, Fig. 1B; Movie EV1, right). We consider that filopodium-type and lamellipodium-type SpTAs serve as precursors of filopodia and lamellipodia, respectively.

### SpTAs spontaneously accumulate at cell periphery and protrusions

**Figure 4:**
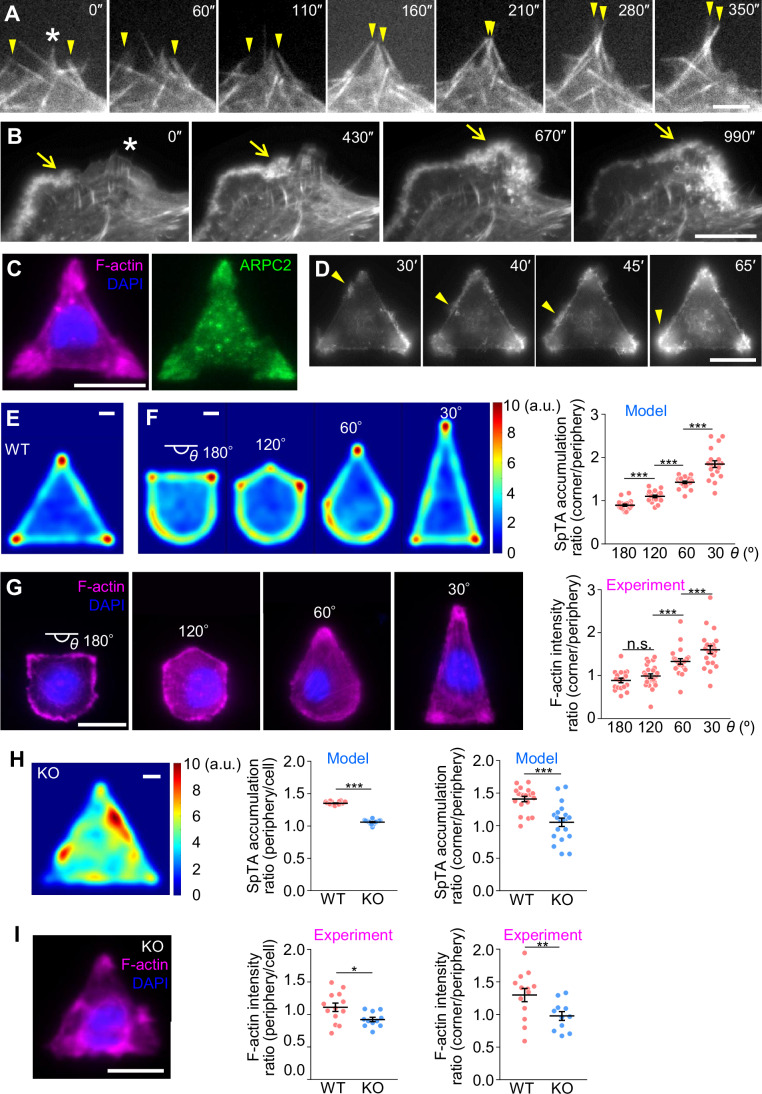
SpTAs mediate F-actin accumulation at the cell periphery and protrusions. (**A**, **B**) Fluorescence time-lapse images of filopodium-type (arrowheads, A) and lamellipodium-type (arrows, **B**) SpTAs which accumulated in protrusive regions (asterisks) in U251 cells expressing EGFP-LifeAct obtained by epifluorescence microscopy. See Movie EV10. Scale bars, 20 µm (**B**); 5 µm (**A**). (**C**) A U251 cell cultured on a laminin-coated triangular adhesive island and stained with Alexa Fluor 594 conjugated phalloidin (for F-actin), anti-ARPC2 antibody, and DAPI (for nucleus). Scale bar, 20 µm. (**D**) Fluorescence time-lapse images of a U251 cell expressing LifeAct-mCherry cultured on a triangular adhesive island. The images were obtained by epifluorescence microscopy. Arrowheads indicate an SpTA travelled along the lateral membrane, resulting in a local accumulation of F-actins at the corner upon arrival. See Movie EV11. Scale bar, 20 µm. (**E**, **F**) Mathematical model data showing SpTA localization in a triangular cell (**E**) and cells with different corner angles, 30°, 60°, 120° or 180° (**F**), presented in arbitrary unit (a.u.). See Movie EV12. Scale bars, 5 µm. The right graph in (**F**) shows quantitative data for the SpTA accumulation at the corners with different corner angles (30–180°, *N* = 3 experiments, *n* = 20 model data). *P* < 0.0001 (180° vs 120°); *P* < 0.0001 (120° vs 60°); *P* < 0.0001 (60° vs 30°). (**G**) U251 cells cultured on laminin-coated adhesive islands with the different corner angles and stained with Alexa Fluor 594 conjugated phalloidin and DAPI. The right graph shows quantitative data for the F-actin accumulation at the corners (30°, *N* = 6 experiments, *n* = 22 cells; 60°, *N* = 8 experiments, *n* = 23 cells; 120°, *N* = 7 experiments, *n* = 26 cells; 180°, *N* = 8 experiments, *n* = 18 cells). *P* = 0.170 (180° vs 120°); *P* < 0.0001 (120° vs 60°); *P* = 0.009 (60° vs 30°). Scale bar, 20 µm. (**H**) Mathematical model data showing SpTA localization in a triangular shootin1b KO cell. See Movie EV12. Scale bar, 5 µm. The right graphs show quantitative data for SpTA accumulation at the edge and corners of WT and shoootin1b KO cells. WT and KO, *N* = 3 experiments, *n* = 20 model data. *P* < 0.0001 (periphery/cell); *P* < 0.0001 (corner/periphery). (**I**) A shootin1b KO#1 U251 cell cultured on a laminin-coated triangular adhesive island and stained with Alexa Fluor 594 conjugated phalloidin and DAPI. The right graphs show quantitative data for F-actin accumulation at the edge and corners of WT and shoootin1b KO cells. WT, *N* = 3 experiments, *n* = 13 cells; KO#1, *N* = 6 experiments, *n* = 11 cells. *P* = 0.0247 (periphery/cell); *P* = 0.0195 (corner/periphery). Scale bar, 20 µm. Data represent means ± SEM. ***P < 0.01; **P < 0.02; *P < 0.05; n.s., not significant. Statistical analyses were performed using the two-tailed unpaired Student′s *t* test (**F**, 180° vs 120° and 120° vs 60°; G, 180° vs 120°; **H**) and two-tailed unpaired Welch′s *t* test (**F**, 60° vs 30°; **I**), the two-tailed Mann‒Whitney *U* test (**G**, 120° vs 60° and 60° vs 30°). Source data are available online for this figure.

We observed translocation of filopodium- and lamellipodium-type SpTAs in protrusive regions through their lateral movement (Fig. 4A,B; Movie EV10), suggesting that their motion is affected by cell shape. To analyze a possible influence of cell shape on the SpTAs, we prepared a triangular pattern of adhesive laminin on the substrate (Appendix Fig. S4). U251 cells showed the triangular shape when cultured on the laminin-coated adhesive island, and F-actins accumulated at the corners of the triangular cells (Fig. 4C). Live imaging of U251 cells labelled with LifeAct-mCherry revealed the dynamic accumulation of F-actins at the corners (Fig. 4D; Movie EV11). SpTAs travelled along the lateral edge, resulting in local accumulation of F-actins at the corners upon arrival (arrowheads). An Arp2/3 complex component, ARPC2, also accumulated at the protruding corners in the absence of local growth factor stimulation (Fig. 4C).

Since the arrival of SpTAs increased F-actins at the protrusive areas (arrowheads, Fig. 4D), we hypothesized that they spontaneously accumulate at cell protrusions. To examine this possibility, we constructed a mathematical model to describe filopodium-type SpTAs, as we could observe F-actins clearly in these structures (Fig. 1B). Our model consists of the following properties of SpTA movement (see “Methods” for details).

1. SpTAs emerge at random locations within the cell and move in random directions (Movie EV1).
2. SpTAs frequently change their direction of translocation and disappear at the end of their lifetime (Movie EV1).
3. SpTAs re-emerge at the location where the preceding SpTA disappeared (see “Methods”).
4. SpTAs located at the cell periphery move laterally along the cell membrane. The velocity of the lateral movement conforms to the cos*θ* of the mean velocity of ventrally translocating SpTAs (*V*_V_), where *θ* is the angle of the direction of F-actins relative to the cell membrane (Fig. 3E).

All the model parameters, i.e., lifetime, length, moving velocity and degree of the change in moving direction, were obtained from our quantitative experimental data (Appendix Fig. S5A–E). The SpTAs described by the model accumulated dynamically at the corners of the triangular cells (Fig. 4E; Movie EV12), as observed in U251 cells. Using this mathematical model, we further analyzed the movement of SpTAs in cells with different corner angles (i.e., 30°, 60°, 120° or 180°; Appendix Fig. S5F) and quantified the relative accumulation of F-actins at the cell periphery and corners (Appendix Fig. S5G). The SpTAs accumulated at the cell periphery (Appendix Fig. S5H). Furthermore, the accumulation of SpTAs at the corner increased as the corner angle decreased from 180 to 30° (Fig. 4F). To validate this model, we cultured U251 cells using the same patterns. As predicted, F-actins accumulated at the periphery (Appendix Fig. S5I) and corners of the cells in a corner angle-dependent manner (Fig. 4G).

We further examined the effect of inhibiting SpTA via knocking out shootin1b. Interestingly, shootin1b KO not only reduced the translocation velocity (Fig. 2F) but also the lifetime (Appendix Fig. S5A–C) of SpTAs. Consistent with our notion that filopodium-type SpTAs are equivalent to the F-actin assemblies in filopodia, the lifetime of filopodia was similar to that of filopodium-type SpTAs and reduced by shootin1b KO (Appendix Fig. S5J). Introduction of these parameters (i.e., SpTA velocity and lifetime derived from shootin1b KO cells) into the model disrupted actin accumulation at the cell periphery and corners (Fig. 4H; Movie EV12). Consistently, shootin1b KO inhibited actin accumulation at the cell periphery and corners of U251 cells (Fig. 4I). Together, these data indicate that SpTAs spontaneously accumulate at cell periphery and protrusions.

### SpTAs drive spontaneous formation of the leading edge for cell polarization

**Figure 5:**
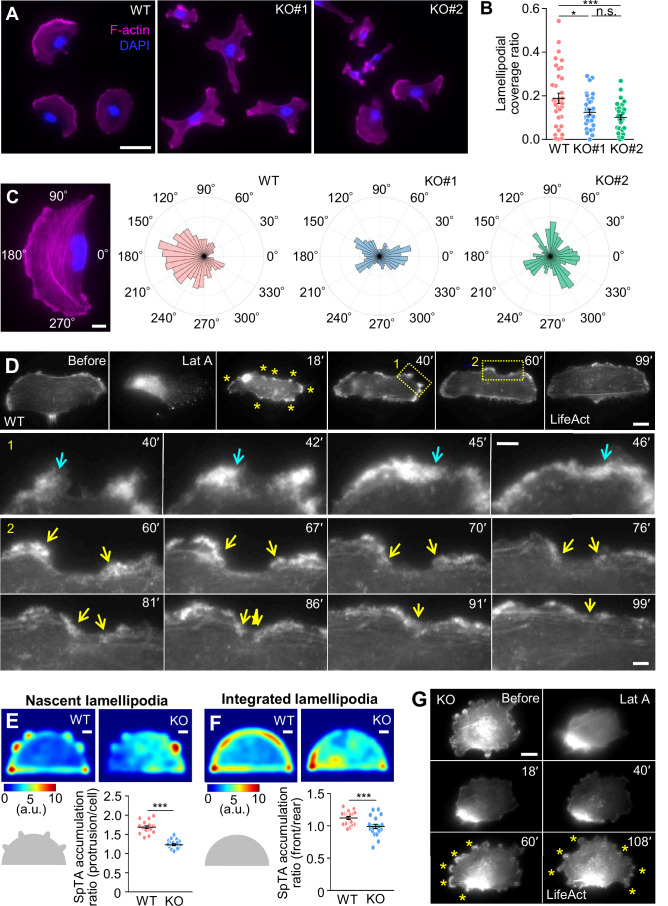
SpTAs drive spontaneous formation of the leading edge for cell polarization. (**A**) WT, shootin1b KO#1 and KO#2 U251 cells stained with Alexa Fluor 594 conjugated phalloidin and DAPI. Scale bar, 50 µm. (**B**) Lamellipodial coverage rate of WT, shootin1b KO#1 and KO#2 U251 cells in (**A**). WT, KO#1, and KO#2, *N* = 3 experiments, *n* = 35 cells. *P* = 0.0223 (WT vs KO#1); *P* = 0.0011 (WT vs KO#2); *P* = 0.588 (KO#1 vs KO#2). Scale bar, 5 µm. (**C**) A WT U251 cell stained with Alexa Fluor 594 conjugated phalloidin and DAPI (left) and histogram of lamellipodia localization at cell periphery (right) of WT, shootin1b KO#1 and KO#2 U251 cells. WT, KO#1, and KO#2, *N* = 3 experiments, *n* = 35 cells. Scale bar, 5 µm. (**D**) Fluorescence time-lapse images of a WT U251 cell expressing LifeAct-mCherry obtained by epifluorescence microscopy: lower panels indicate enlarged time-lapse images of the areas indicated by the dashed yellow boxes 1 and 2. The images were taken before and after the treatment with 100 nM latrunculin A (Lat A), and at the indicated times after the Lat A washout. The asterisks indicate F-actin-enriched nascent lamellipodia. Yellow and blue arrows indicate enlargement and lateral expansions of nascent lamellipodia, respectively. See Movie EV13. Scale bars, 20 µm; 5 µm (enlarged views). (**E**, **F**) Mathematical model data showing SpTA localization in WT and shootin1b KO cells bearing nascent lamellipodia (**E**) and integrated lamellipodium (**F**), presented in arbitrary unit (a.u.). The panels below describe the cell shape (40 µm width). The graphs below show quantitative data for SpTA accumulation at the nascent lamellipodia (**E**) and integrated lamellipodia (**F**) of WT and shoootin1b KO cells. Scale bars, 5 µm. WT and KO, *N* = 3 experiments, *n* = 20 model data. *P* = 4.15 × 10^−13^ (**E**); *P* = 0.00243 (**F**). See also Appendix Fig. S6B,C. (**G**) Fluorescence time-lapse images of a shootin1b KO#1 U251 cell expressing LifeAct-mCherry obtained by epifluorescence microscopy. The images were taken before and after the treatment with 100 nM Lat A, and at the indicated times after the Lat A washout. The asterisks indicate nascent lamellipodia. See Movie EV13. Scale bar, 20 µm. See also Appendix Fig. S6D. Data represent means ± SEM. ***P < 0.01; *P < 0.05; n.s., not significant. Statistical analyses were performed using the two-tailed one-way ANOVA with Tukey’s post hoc test (**B**) and the two-tailed unpaired Student′s *t* test (**E**, **F**). Source data are available online for this figure.

Next, the role of SpTAs in cell morphogenesis was analyzed using U251cells with the deformable plasma membrane. Wild-type (WT) cells typically exhibited a polarized morphology with a large lamellipodium (Fig. 5A). On the other hand, inhibition of SpTAs by shootin1b KO resulted in the fragmentation of lamellipodia (Fig. 5A), leading to a decrease in the lamellipodial coverage ratio at the cell periphery (Fig. 5B; Appendix Fig. S6A). Accordingly, the unilateral integration of lamellipodium was disrupted, resulting in a decrease in the degree of cell polarity (Fig. 5C). To analyze how SpTAs drive cell polarization, we first depolymerized F-actins in U251 cells using latrunculin A, and then monitored the cells’ recovery after latrunculin A washout (Gerisch et al, 2004). Treatment of WT U251 cells with 100 nM latrunculin A disrupted their polarized morphology (Fig. 5D; Movie EV13). During recovery following latrunculin A washout, the cells formed multiple foci of small lamellipodia enriched with F-actins (asterisks, Fig. 5D). The foci then grew and moved laterally (cyan arrows) or expanded laterally (yellow arrows), integrating into a large lamellipodium. Through these processes, the cells eventually formed a leading edge and acquired polarity.

We further monitored the motion of SpTAs in a two-dimensional (2D) model of cells bearing protrusions that mimic the nascent and integrated lamellipodia. SpTAs in the model highly accumulated in the nascent lamellipodia (Fig. 5E; Appendix Fig. S6B) and moderately accumulated in the integrated lamellipodium located at the convex leading edge (Fig. 5F; Appendix Fig. S6C). Introduction of the parameters derived from shootin1b KO cells (Fig. 2F; Appendix Fig. S5A–C) into the model inhibited SpTA accumulation at the nascent and integrated lamellipodia (Fig. 5E,F).

Consistently with the modeling data, shootin1b deletion in glioma cells inhibited the lateral movement, expansion, and integration of nascent lamellipodium (asterisks, Fig. 5G; Movie EV13). This in turn delayed the onset of cell migration after recovery from the latrunculin A treatment (Appendix Fig. S6D). As previously reported, shootin1b also mediates the generation of traction force at the leading edge through its interaction with F-actins (Minegishi et al, 2018). Therefore, the impairment of lamellipodial integration resulting from shootin1b KO can be attributed to the combined effects of reduced accumulation of SpTAs at lamellipodia (Fig. 5E,F) and diminished force generation there. Taken together, we conclude that SpTAs of glioma cells drive spontaneous formation of the leading edge by expanding the nascent lamellipodia.

## Discussion

Treadmilling is an inherent property of actin dynamics that allows for the directional polymerization and disassembly of F-actin arrays. The present study reports SpTAs that translocate within cells as self-propelled “particles” rather than “wave”-like propagation of chemical reactions. The arrival of SpTAs at the cell periphery pushes the membrane to form protrusions. Furthermore, SpTAs accumulate into protrusions, leading to their robust growth and spontaneous formation of the leading edge for cell migration.

### SpTA translocation by actin polymerization and disassembly

**Figure 6:**
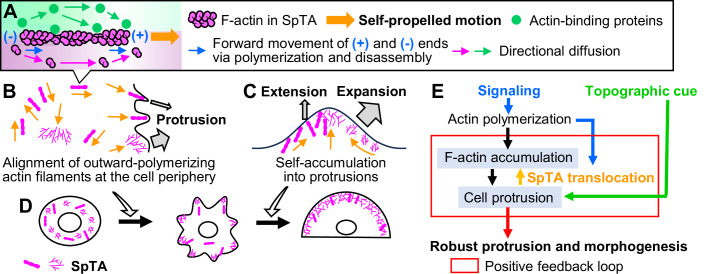
A model of the translocation mechanisms of SpTAs. (**A**) During the translocation, the front and rear ends of SpTAs move forward via polymerization and disassembly, respectively (blue arrows). Shootin1b facilitates the SpTA translocation by impeding their retrograde flow (Fig. 2B, left). As the local concentrations of actin subunits and actin-binding proteins decrease at the front by polymerization and increase at the rear by disassembly, they diffuse along the local concentration gradients (magenta and green arrows). Accompanied by the directional diffusion, SpTAs mediate the translocation of actin and actin-binding proteins. (**B**) SpTA arrival at the cell periphery aligns randomly oriented F-actins towards outward polymerization, which in turn pushes the membrane to protrude (gray arrows). (**C**) SpTAs accumulate at cell protrusions (yellow arrows), leading to their further growth (gray arrows). (**D**) Processes of spontaneous polarization of glioma cells. Through local protrusion (**B**) and lateral expansion (**C**), nascent lamellipodia are formed and integrated into a large leading edge for polarization. (**E**) Accumulation of SpTAs at protrusions (yellow arrow) constitutes a positive feedback interaction with actin-mediated cell protrusion (red box), thereby conferring robustness to the protrusions (red arrow) cell morphogenesis. SpTAs are regulated under cell signaling (blue arrows). This system can also respond to extracellular topographic cues that deform the plasma membrane from outside (green arrow).

During SpTA translocation (yellow arrow, Fig. 6A), their front move forward (blue arrow) via incorporation of ATP-bound actin subunits at the plus end. This is followed by ATP hydrolysis within the filaments, which in turn leads to disassembly of the filaments from the minus ends and forward movement of the rear (blue arrow). This is further regulated by actin-binding proteins. The ADP bound to the disassembled actin subunits is replaced with ATP, allowing the subunits to be reused in polymerization (magenta arrows). Shootin1b facilitates SpTA translocation by impeding their retrograde flow (Fig. 2B, left). During SpTA translocation, local concentrations of actin subunits and actin-binding proteins decrease at the front by polymerization and increase at the rear by disassembly, resulting in their diffusion along the local concentration gradients (green and magenta arrows, Fig. 6A). Accompanied by this directional diffusion, SpTAs translocate actin and actin-binding proteins.

### SpTA and actin wave

We previously reported F-actin assemblies translocating along axons by their directional polymerization and disassembly (Katsuno et al, 2015). This F-actin translocation was originally identified by Ruthel and Banker (Ruthel and Banker, 1998, 1999) and called “waves”. Given this historical context, we called them as actin waves (Inagaki and Katsuno, 2017). Here, we show SpTAs that translocate by directional polymerization and disassembly in non-neuronal cells. SpTAs are equivalent to the previously reported actin wave (Katsuno et al, 2015) since they both translocate via the same mechanism. Notably, they both move as discrete “particles” rather than propagating uniformly as “waves”, and their translocation is not based on the mechanism of the wave propagation. Therefore, we propose referring to them both as SpTAs.

SpTAs potentially explain a wide range of intracellular actin dynamics. First, dynamic F-actins undergo propagation in various cells (Carlsson, 2010; Allard and Mogilner, 2013; Inagaki and Katsuno, 2017; Beta et al, 2023). Their propagations have been mainly explained by the reaction-diffusion model or Turing model, where the excitable component that stimulates actin polymerization at the front simultaneously stimulates the inhibitory component at the rear to depolymerize F-actins (Weiner et al, 2007; Allard and Mogilner, 2013; Inagaki and Katsuno, 2017; Beta et al, 2023). A variety of signaling and mechanical networks have been proposed to link the excitable and inhibitory components, but a general mechanism has not yet been established (Beta et al, 2023). Since ATP hydrolysis is an intrinsic property of the actin molecules incorporated in the filaments, stimulation of actin polymerization in SpTAs spontaneously leads to the F-actin disassembly at the rear. By directly linking the actin polymerization at the front to the depolymerization at the rear, treadmilling could be a versatile mechanism for F-actin assembly translocation.

In addition, translocation of F-actin assemblies to the cell periphery has been reported to induce the lamellipodia formation (Bretschneider et al, 2009; Zhang et al, 2018), as SpTAs did here (Fig. 3A–C). Since the polymerizing ends of SpTAs are located at the front (Fig. 6A), the arrival of SpTAs at the cell periphery aligns the polymerizing ends outward, thereby pushing the membrane to form filopodia and lamellipodia. Thus, SpTAs explains how the arrival of F-actin assemblies drive membrane protrusions (Fig. 6B).

### Protrusions trap SpTAs

The accumulation of SpTAs in protrusive regions (Fig. 4C–G) is a key property of self-propelled particles colliding with a boundary (Kaiser et al, 2012). When SpTAs encounter the membrane border, they further undergo lateral translocation, depending on the direction of F-actins relative to the membrane. This lateral movement traps outward-polymerizing F-actins and actin-binding proteins at protrusive regions (yellow arrows, Fig. 6C) and explains previous observations that different cell types confined to square adhesive islands preferentially extend filopodia and lamellipodia from their protrusive areas (Parker et al, 2002). This mechanism is distinct from the previously reported actin accumulation mechanism via curvature-sensitive actin nucleation (Saarikangas et al, 2015; Sadhu et al, 2021). The former accumulates F-actins by trapping them at protrusive regions, while the latter accumulates F-actins by promoting actin polymerization there. The modeling data of F-actin accumulation (Fig. 5E,F) also support this notion, as our model do not include the curvature-sensitive actin nucleation. The curvature-sensitive actin nucleation is mediated by the curved proteins, so it would sense nanoscopic membrane curvatures. On the other hand, the “trapping” mechanism of SpTA enables F-actin to accumulate in the protrusions of a broader size range, from local protrusions to the global cellular leading edge (Fig. 5E,F), as well as at different angles (Fig. 4F,G).

### SpTAs in spontaneous cell protrusion and morphogenesis

The F-actin accumulation at cell protrusions through SpTA translocation (yellow arrow, Fig. 6E) constitutes a positive feedback interaction with the F-actin-driven cell protrusion (red box). This interaction confers robustness to the protrusions (red arrow) and leads to their further growth (gray arrows, Fig. 6C). Since translocation of SpTAs into the protrusion (yellow arrows, Fig. 6C) results in their lateral decrease, the self-propelled motion of SpTAs also suppresses lateral protrusive activities. During spontaneous polarization of glioma cells, nascent protrusions were integrated into a large leading edge through their lateral movement and expansion (Fig. 6D), accompanied by a relative decrease in SpTAs at the rear (Fig. 5D; Movie EV13). These processes would work in concert with other cytoskeletal dynamics, including actomyosin contraction at the rear (Vassilev et al, 2017; Hadjitheodorou et al, 2021) to spontaneously polarize cells and initiate cell migration. Spontaneous polarization would also be supported by membrane tension generated by actin-based protrusions (Tsujita et al, 2015; De Belly et al, 2023) as well as by membrane deformation through curved membrane-bound proteins with adhesion (Sadhu et al, 2021).

F-actins undergo outward polymerization not only at the filopodia and lamellipodia but also in various cell protrusions, such as growth cones and dendritic spines of neurons (Suter and Forscher, 2000; Frost et al, 2010), microvilli (Gov, 2006; Meenderink et al, 2019), cell-cell adhesion sites (Efimova and Svitkina, 2018) and microridge (Lam et al, 2015) of epithelial cells, hair cell stereocilia (Rzadzinska et al, 2004), cancer cell invadopodia (Rottner and Schaks, 2019), and phagocytic cups (Rottner and Schaks, 2019). Our modeling data showed that SpTAs self-accumulate at small protrusive and large convex regions (Fig. 5E,F), as well as protrusions with different apex angles (Fig. 4F). Different actin-binding proteins and physical interactions could control the mode of protrusion, either extension or expansion, by altering the shape of the F-actin assembly (Swaney and Li, 2016; Carlier and Shekhar, 2017; Huber et al, 2018; Rottner and Schaks, 2019). Future research is needed to examine the potential roles of SpTAs in the formation of various types of actin-based cell protrusions.

### SpTAs in environmental cue-driven cell morphogenesis

Because actin polymerization and disassembly are regulated by signaling pathways (Pollard and Borisy, 2003; Bernstein and Bamburg, 2010; Wang et al, 2015; Carlier and Shekhar, 2017; Rottner and Schaks, 2019; Goode et al, 2023), the formation, translocation, and disappearance of SpTAs are subject to the regulation by extracellular chemical cues and cell signaling (blue arrows, Fig. 6E). Inhibition of formins or VASP reduced the SpTA translocation velocity (Fig. 2E), indicating that SpTAs are under the regulation of these molecules. Formins are located at the plasma membrane, and a recent study reported that formins attached to intracellular vesicles promote actin polymerization in the cytoplasm (Frank et al, 2023). These actin regulators would maintain the polymerizing ends of SpTAs along the plasma membrane and in the cytoplasm. Additionally, SpTAs could mediate cellular responses to extracellular topographic cues that deform the plasma membrane from outside of the cell (green arrow, Fig. 6E), as they accumulate at the protrusive and convex regions. This would allow migrating cells to navigate flexibly through mechanical barriers and tissue pores in three-dimensional environments (Weiner et al, 2007; Yamada and Sixt, 2019; Hadjitheodorou et al, 2023). SpTA regulation by environmental cues that tune spontaneous cell morphogenesis are important issues for future analyses.

## Methods

**Table:** Reagents and tools table

Reagent/resource	Reference or source	Identifier or catalog number
**Experimental models**		
U251	A gift from Drs. Masatoshi Takeichi (Riken) and Shintaro Suzuki (Kwansei Gakuin Univ)	Vassilev et al, 2017
U-251 Shootin1b-KO	This paper	N/A
COS7	Riken BRC	Cat# RCB0539
HEK293T	ATCC	Cat# CRL_3216
**Recombinant DNA**		
pFN21A-HaloTag-actin	Minegishi et al, 2018	N/A
pEGFP-N1-LifeAct	This paper	N/A
pmcherry-N1-LifeAct	This paper	N/A
pSpCas9(BB)-2A-Puro (PX459) V2.0	Addgene	Cat# 62988
pmNeonGreen-Fascin	This paper	N/A
pcDNA6.2-GW/miR-VASP RNAi	This paper	N/A
**Antibodies**		
Rabbit polyclonal anti-shootin1	Toriyama et al, 2006	N/A
Rabbit polyclonal anti-shootin1b	Higashiguchi et al, 2016	N/A
Rabbit polyclonal anti-VASP	Proteintech	Cat# 13472-1-AP
Goat polyclonal anti-NCAM-L1	Santa Cruz Biotechnology	Cat# sc-1508
Mouse monoclonal anti-cortactin	Millipore	Cat# 05-180
Mouse monoclonal anti-actin	Millipore	Cat# MAB1501R
Rabbit polyclonal anti-ARPC2	Millipore	Cat# 07-227
Alexa 594 conjugated donkey anti-rabbit	Jackson ImmunoResearch Labs	Cat# 711-585-152
Alexa 488 conjugated goat anti-rabbit	Thermo Fisher Scientific	Cat# A-11008
Alexa 488 conjugated goat anti-mouse	Thermo Fisher Scientific	Cat# A-11029
HRP conjugated donkey anti-rabbit	Cytiva	Cat# NA934
HRP conjugated goat anti-mouse	Thermo Fisher Scientific	Cat# AP308P
**Oligonucleotides and other sequence-based reagents**	**Sequence (5’-3’)**	**Source**
Shootin1b-Exon1 F	CACCGGCAGCTCATTACCAGTCTGA	This paper
Shootin1b-Exon1 R	AAACTCAGACTGGTAATGAGCTGCC	This paper
LifeAct F	TCGAGATGGGTGTCGCAGATTTGATCAAGAAATTCGAAAGCATCTCAAAGGAAGAAGGG	This paper
LifeAct R	GATCCCTTCTTCCTTTGAGATGCTTTCGAATTTCTTGATCAAATCTGCGACACCCATC	This paper
Fascin Fw	AGAGGATCTGAGCCCGGGCGGATCCATGACCGCCAACGGCACAGCCGA	This paper
Fascin Rv	CGAATTCCTGCAGCCCGGGGGATCCCTAGTACTCCCAGAGCGAGGCGG	This paper
The targeting sequence of VASP, corresponding to nucleotides 1068-1088 in the coding region of human VASP	GAAGGAATTGCAGAAAGTGAA	This paper
**Chemicals, enzymes and other reagents**		
Alexa 594 phalloidin	Thermo Fisher Scientific	Cat# A12381
DAPI	Thermo Fisher Scientific	Cat# D1306
Poly-D-lysine	Sigma	Cat# P6407-5MG
Laminin	Wako	Cat# 120-05751
HaloTag TMR ligand (5 mM)	Promega	Cat# G8251
LIPIDURE (MPC-polymer)	NOF	Cat# CM5206
3-phenylaminopropyltrimethoxysilane	Shin-Etsu	Cat# LS-4500
**Software**		
ImageJ software	National Institutes of Health	https://imagej.nih.gov/ij/index.html; RRID: SCR_003070
GraphPad Prism7	GraphPad Software	https://www.graphpad.com/; RRID: SCR_002798
MATLAB Version R2021a	Mathworks	https://www.mathworks.com/products/new_products/release2021a.html; RRID: SCR_001622
ZEN software	Carl Zeiss	https://www.zeiss.com/microscopy/int/products/microscope-software/zen.html; RRID: SCR_013672
Metamorph	Molecular Devices	https://www.moleculardevices.com/products/cellular-imaging-systems/acquisition-and-analysis-software/metamorph-microscopy; RRID:SCR_002368
Las X	Leica	https://www.leica-microsystems.com/products/microscope-software/p/leica-las-x-ls/; RRID:SCR_013673
**Other**		
Source data (Mendeley data)	This paper	https://data.mendeley.com/datasets/ymmcbh92v6/3
The MATLAB codes used for mathematical modeling	This paper	https://github.com/KioYagami/actin-wave/tree/master

### Cell culture and transfection

A subline of glioma U251 cells (Vassilev et al, 2017) and COS7 cells were seeded on glass-bottom dishes (Matsunami, #D11130H) coated with 100 μg/ml poly-D-lysine (Sigma) and 5 μg/ml laminin (Lonza), and cultured in DMEM high glucose medium (Nacalai, #08458-16) supplemented with 10% fetal bovine serum (Japan Bio Serum) at 37 °C and 5% CO_2_. HEK293T cells (ATCC) were cultured in DMEM high glucose medium (Nacalai, #08458-16) supplemented with 10% fetal bovine serum (Japan Bio Serum) as described previously (Baba et al, 2018). They were transfected with vectors using Polyethylenimine MAX (PEI MAX, Polysciences) following the manufacturer’s protocol. No authentication was done for cell lines. All cell lines were found to be free of mycoplasma contamination.

### Generation of shootin1b KO U251 cells by CRISPR-Cas9

The guide oligonucleotide sequence (forward: 5´-CACCGGCAGCTCATTACCAGTCTGA-3´; reverse: 5´-AAACTCAGACTGGTAATGAGCTGCC-3´), corresponding to nucleotides 30–49 in the coding region of human shootin1b, were selected by an online CRISPR Cas9 Design tool (http://crispr.mit.edu/). The pSpCas9 (BB)-2A-Puro (pX459) V2.0 plasmid (Addgene, #62988) was linearized using by BbsI-HF (New England Biolabs, #R3539S), then the guide oligonucleotides were annealed and inserted into the vector using Ligation high Ver.2 (TOYOBO, #LGK-201). U251 cells were transfected with the plasmid, cultured individually in 96-well plates, and screened using 4 μg/ml puromycin. Shootin1b depletion in KO cells was confirmed by immunoblot analyses (Appendix Fig. S3D).

### Micropatterning of laminin on the substrate for cell adhesion

Micropatterns of laminin for cell adhesion were produced on a glass surface following our previously established procedure (Okano et al, 2013), with modifications (Appendix Fig. S4). The glass-bottom dish (Iwaki, #3961-035) was cleaned using ultrasonic waves for 5 min with special grade ethanol, followed by rinsing with water and additional 5-min ultrasonic cleaning with pure water. After the cleaning, the dish was air-dried in a clean room at room temperature. 3-Phenylaminopropyltrimethoxysilane (2%, Shin-Etsu, #LS-4500) was dissolved in a 2% solution of acetic acid in water and then poured onto the glass-bottom dish, followed by incubation for 30 min. After being washed twice with water, the dish was dried at 70 °C for 1 h. This process resulted in the covalent bonding of the 3-phenylaminopropyl group with the glass surface, which made it hydrophobic. Any residues resulting from the reaction were eliminated by ultrasonically cleaning with special grade ethanol for 5 min.

The hydrophobic glass surface was coated with 100 µL of ethanol containing 0.2% of MPC (2-methacryloyloxyethyl phosphorylcholine) polymer (NOF, #Lipiduro-CM5206). After the reaction for 10–15 s, the coating solution was removed and left to dry overnight at room temperature in the dark. This resulted in the formation of an MPC polymer film on the glass (Appendix Fig. S4). Then, the dish was incubated with 2 ml H_2_O overnight.

The cell non-adhesive MPC-polymer layer was removed by a programmed raster scanning of a femtosecond laser (center wavelength 800 nm, pulse width 130 fs, pulse energy 100 nJ, transmission frequency 5 kHz, Solstice, Spectra-Physics) focused with an objective lens 20 × 0.46 NA (Olympus, UMPlanFl) at a speed of 400 µm/s on the glass surface. The raster scanning patterns are shown (Appendix Figs. S4 and S5F). Then, the cell-adhesive patterns of laminin were produced by coating the exposed glass surface with poly-D-lysine (PDL) and laminin. The cell-adhesive patterns were confirmed by coating with green fluorescent-HiLyte 488-Laminin (Cytoskeleton, #LMN02-A) (Appendix Fig. S4).

### Immunocytochemistry

Immunocytochemistry was performed as described previously (Katsuno et al, 2015). Cultured cells were fixed with 3.7% formaldehyde in Krebs buffer (118 mM NaCl, 4.7 mM KCl, 1.2 mM KH_2_PO_4_, 1.2 mM MgSO_4_, 4.2 mM NaHCO_3_, 2 mM CaCl_2_, 10 mM glucose, 400 mM sucrose, 10 mM HEPES pH 7.0) for 20 min on ice and 10 min at room temperature, followed by treatment for 15 min with 0.05% Triton X-100 in PBS on ice and 10% fetal bovine serum in PBS for 1 h at room temperature. The cells were incubated with a primary antibody diluted in PBS containing 10% fetal bovine serum overnight at 4 °C. They were washed with PBS, and then incubated with secondary antibody diluted in PBS for 1 h at room temperature. After washing with PBS, the cells were stained with Alexa Fluor 594 conjugated phalloidin (1:100, Thermo Fisher Scientific) and DAPI (1:1000, Thermo Fisher Scientific) for 30 min at room temperature. The fluorescence and phase-contrast images of U251 cells were acquired a fluorescence microscope (Carl Zeiss, Axioplan2) equipped with an objective lens 40 × 0.75 NA (Carl Zeiss, plan-Neofluar), a charge-coupled device camera (Carl Zeiss, AxioCam MRm) and imaging software (Carl Zeiss, ZEN), and a TIRF microscope (Olympus, IX81) equipped with a CMOS camera (Hamamatsu, ORCA Flash4.0LT), an objective lens 100 × 1.49 NA (Olympus, UAPON), and MetaMorph software.

### Immunoblot

Cultured U251 or HEK293T cells were lysed with RIPA buffer (50 mM Tris-HCl, pH 8.0), 1 mM EDTA, 150 mM NaCl, 1% Triton X-100, 0.1% SDS, 0.1% sodium deoxycholate, 1 mM DTT, 1 mM PMSF, and 0.01 mM leupeptin), and incubated for 10 min at 4 °C. Subsequently, the cell lysate was centrifuged at 15,000 rpm for 15 min at 4 °C. The supernatant was mixed with an equal volume of 2× SDS sample buffer (131 mM Tris-HCl, pH 6.8, 21% glycerol, 4% SDS, 0.05% bromophenol blue and 5% β-mercaptoethanol). The mixture was incubated for 5 min at 95 °C, followed by SDS-polyacrylamide gel electrophoresis. Immunoblotting was performed as described previously (Minegishi et al, 2018).

### Antibodies

The following primary antibodies were used: anti-shootin1 (rabbit, 1:400) (Higashiguchi et al, 2016); anti-shootin1b (rabbit, 1:2000) (Higashiguchi et al, 2016); anti-NCAM-L1 (mouse, 1:1000, Santa Cruz Biotechnology, #sc-514360); anti-cortactin (mouse, 1:1000, Millipore, #05-180); anti-actin (mouse, 1:1000, Millipore, #MAB1501R); anti-ARPC2 (rabbit, 1:1000, Millipore, #07-227); anti-VASP (rabbit, 1:5000, Proteintech, # 13472-1-AP). The following secondary antibodies were used: anti-rabbit (Alexa 594 conjugated donkey, 1:1000, Jackson ImmunoResearch Labs, #711-585-152); anti-rabbit (Alexa 488 conjugated goat, 1:1000, Thermo Fisher Scientific, #A-11008); anti-mouse (Alexa 488 conjugated goat, 1:1000, Thermo Fisher Scientific, #A-11029); anti-rabbit (HRP conjugated donkey, 1:2000, GE Healthcare, #NA934); anti-mouse (HRP conjugated goat, 1:5000, Thermo Fisher Scientific, #AP308P).

### DNA construction

To generate pEGFP-N1-LifeAct vector, the annealed LifeAct (forward: 5´-TCGAGATGGGTGTCGCAGATTTGATCAAGAAATTCGAAAGCATCTCAAAGGAAGAAGGG-3´; reverse: 5´-GATCCCTTCTTCCTTTGAGATGCTTTCGAATTTCTTGATCAAATCTGCGACACCCATC-3´) was fused to N-terminal of EGFP-N1 vectors (Clontech). To generate pLifeAct-mCherry-N1 vector, the annealed LifeAct was fused to N-terminal of mCherry-N1 vectors (Takara, #632523). Generation of pFN21A-HaloTag-actin was described previously (Minegishi et al, 2018). Full-length cDNA of human Fascin was obtained by PCR amplification of U251 cell cDNA with the primers Fascin Fw (5′-AGAGGATCTGAGCCCGGGCGGATCCATGACCGCCAACGGCACAGCCGA-3′) and Fascin Rv (5′-CGAATTCCTGCAGCCCGGGGGATCCCTAGTACTCCCAGAGCGAGGCGG-3′), and subcloned into pmNeonGreen-C1 vector (Allele Biotechnology) using InFusion HD (Takara). To generate a VASP microRNA expressing vector, we used a Block-iT Pol II miR RNAi expression vector kit (Thermo Fisher Scientific). The targeting sequence of VASP (5′-GAAGGAATTGCAGAAAGTGAA-3′, corresponding to nucleotides 1068-1088 in the coding region of human VASP) was cloned and inserted into the pcDNA6.2-GW/miR expression vector. To confirm the reduction of human VASP expression, immunoblot was performed using lysates of HEK293T cells (Appendix Fig. S3A).

### TIRF and epifluorescence microscopy

U251 cells were cultured on glass-bottom dishes coated with PDL and laminin. Actin dynamics in close proximity to the ventral plasma membrane were visualized using the F-actin marker EGFP-LifeAct or LifeAct-mCherry under TIRF microscopy. In addition, to differentially visualize SpTAs translocating in close proximity to the ventral plasma membrane and those detached from the membrane, we combined TIRF microscopy, which visualizes the region in close proximity to the ventral plasma membrane, and epifluorescence microscopy, which visualizes the entire cell thickness (Fig. 1E). U251 cells expressing LifeAct-mCherry and EGFP-LifeAct were cultured on glass-bottom dishes coated with PDL and laminin. Prior to observation, the cells were incubated in DMEM/F-12 Ham medium (Sigma) supplemented with 10% FBS (Japan Bio Serum). The TIRF laser was used to observe fluorescence images of LifeAct-mCherry, while the epifluorescence laser was used to observe fluorescence images of EGFP-LifeAct. For Appendix Fig. S1D, the TIRF laser was used to observe fluorescence images of Lifeact-mCherry and mNeonGreen-fascin. The observations were made with a TIRF microscope (Olympus, IX81) equipped with a CMOS camera (Hamamatsu, ORCA Flash4.0LT) or electron-multiplying CCD camera (Andor, Ixon Du888), an objective lens 100×1.49 NA or 150 × 1.45 NA (Olympus, UAPON), and MetaMorph software. Images were acquired every 10 or 60 s.

### Fluorescent speckle imaging of SpTA by TIRF microscopy

Actin dynamics within SpTAs were examined by labelling F-actins with EGFP-LifeAct, and monitoring actin molecules in SpTAs by speckle imaging of HaloTag-actin (Minegishi et al, 2021). U251 cells expressing HaloTag-actin and EGFP-LifeAct were cultured on glass-bottom dishes coated with PDL and laminin. Prior to observation, the cells were treated with 0.05 μM HaloTag TMR ligand (Promega) in the culture medium and incubated for 1 h at 37 °C. The ligand was washed with PBS, and the cells were then incubated in DMEM/F-12 Ham medium (Sigma, #D8062) supplemented with 10% FBS (Japan Bio Serum). The fluorescent speckles of HaloTag-actin and EGFP-LifeAct were observed using a TIRF microscope (Olympus, IX81) equipped with a CMOS camera (Hamamatsu, ORCA Flash4.0LT), an objective lens 150 × 1.45 NA (Olympus, UAPON), and MetaMorph software. Fluorescence images were acquired every 10 s. Actin flow velocity was calculated by tracing the speckles of HaloTag-actin in SpTAs using ImageJ (Fiji version). Actin polymerization rate was calculated as the sum of SpTA migration velocity, visualized by EGFP-LifeAct, and HaloTag-actin flow velocity, as reported (Minegishi et al, 2021).

### 3D imaging by confocal deconvolution microscopy

U251 cells expressing HaloTag-actin and EGFP-LifeAct were cultured in 2D (on glass-bottom dishes coated with PDL and laminin) or 3D (75% Matrigel) environment. Before observation, the cells were incubated with 0.05 μM HaloTag TMR ligand (Promega) in the culture medium and incubated for 1 h at 37 °C. The ligand was washed with PBS, and the cells were then incubated in DMEM/F-12 Ham medium (Sigma) supplemented with 10% FBS (Japan Bio Serum). The fluorescent speckles of HaloTag-actin and EGFP-LifeAct were observed using a confocal microscope (Leica, Stellaris 8) equipped with an objective lens 100×1.40 NA (Leica, HC PL APO CS2) and Las X software. Deconvolution of 3D image stacks were obtained by Las X software.

### Mathematical modelling

To investigate whether SpTAs mediate the accumulation of F-actins at the protrusions of cells confined to adhesive islands (Fig. 4C), we constructed a mathematical model that describes the movement of intracellular SpTAs. Our model is based on quantitative data obtained from experimental observations: we focused on filopodium-type SpTAs because it is difficult to observe individual F-actins in lamellipodium-type SpTAs, and filopodium-type and lamellipodium-type SpTAs are interchangeable (Fig. 1B). For simplicity, the present model describes the translocation of the SpTAs, not a detailed mechanism of SpTA translocation (Katsuno et al, 2015). The model depicts the processes of SpTA emergence and disappearance, as well as changes in the direction of SpTA translocation.

#### Emergence and disappearance of SpTAs

Our experimental data indicate that SpTAs emerge widely in the cytoplasm, move in random directions, and then disappear (Movie EV1). In our model, the emergence locations and translocation directions of SpTAs are randomly chosen at the start of the modeling. The model data are updated every 1 s (Δ*t* = 1 s), generating one SpTA in the cell with a probability of 10%. The lifetimes of SpTAs in WT and shootin1b KO cells, quantified by live cell imaging, were modeled using gamma distributions (Appendix Fig. S5A,B).

The length (*L*) and translocation velocity (*V*) of SpTAs are also derived from experimental data (Fig. 2F; Appendix Fig. S5E); model uses their average values for simplicity. SpTAs located at the cell periphery move laterally along the cell membrane (Fig. 3A). The velocity of the lateral movement was modelled as cos*θ* of the mean velocity of SpTAs (Fig. 2F), where *θ* is the angle of the direction of SpTA movement with respect to the cell membrane (Fig. 3D).

The local concentration of actin subunits increases immediately after the disappearance (complete disassembly) of SpTA (Fig. 3C). Since actin polymerization depends on the local concentration of actin subunits, our model assumes that an SpTA reemerges at the location where the preceding SpTA disappeared and translocates in a random direction.

#### Change in the direction of SpTA translocation

SpTAs frequently change the direction of their translocation (Movie EV1). The change in direction was quantified every 50 s from live cell imaging data (Appendix Fig. S5D). As the angular distribution of these changes was symmetric with a zero peak, we modelled it using a Gaussian distribution (Appendix Fig. S5D).

#### Model parameters estimated from experimental data

- Translocation velocity of SpTA (Fig. 2F): 2.0 µm/min (WT), 1.5 µm/min (KO#1)
- SpTA length (Appendix Fig. S5E): 4.1 µm
- Parameters of the gamma distribution representing lifetime variation; estimated from quantitative data (Appendix Fig. S5A,B): *α* = 3.0, *β* = 119 (WT), *α* = 2.4, *β* = 106 (KO#1)
- Parameters of the Gaussian distribution representing SpTA directional change; quantified from experimental data (Appendix Fig. S5D): *μ* = −0.61, *σ* = 11.

### Analyses of F-actin accumulation and lamellipodial formation

Accumulation of F-actins at the cell periphery was determined by dividing the average fluorescence intensity of F-actins within a 2 μm-wide region from the cell periphery (green region, Appendix Fig. S5G) by the average intensity of F-actins in the whole cell region (blue region). Accumulation of F-actins at the cell protrusions was determined by dividing the average intensity of F-actins at the corner regions (yellow region: 2 μm-wide and 5 μm-long regions in both sides of the corner, Appendix Fig. S5G) by the average intensity of F-actins in the peripheral region (green region). Accumulation of F-actins at the nascent lamellipodia was determined by dividing the average fluorescence intensity of F-actins in protrusive regions (green regions, Appendix Fig. S6B) by the average intensity of F-actins in the whole cell region (blue region). Accumulation of F-actins at the integrated lamellipodium was determined by dividing the average intensity of F-actins at the convex leading edge (yellow region: 4 μm-wide, Appendix Fig. S6C) by the average intensity of F-actins in the rear region (green region: 4 μm-wide). Lamellipodial coverage rate was calculated by dividing the total length of lamellipodia (yellow line, Appendix Fig. S6A) by the perimeter of the cell (green line). The fluorescence intensity of F-actins was analyzed using ImageJ (Fiji version). Lamellipodia were defined as regions of the cell periphery where the fluorescence intensity exceeded the mean intensity + SD of the whole cell body. The cell edge closest to the nucleus was designated as 0°. The frequency of lameripodia localization along the cell periphery was represented as a polar histogram, divided into 10° intervals. The polar histogram of the lamellipodia localization was generated using MATLAB (Fig. 5C).

### Quantification and statistical analyses

Statistical analyses were performed using Excel (Microsoft 365) or GraphPad Prism 7 (GraphPad Software). For samples with more than 7 data points, the D′Agostino–Pearson normality test was used to determine whether the data followed a normal distribution. In cases where the number of data points was between 3 and 7, the Shapiro‒Wilk test was used for the normality test. We also tested the equality of variation with the F test for two independent groups that followed normal distributions. Significance tests were performed as follows: (1) two-tailed unpaired Student′s *t* test to compare normally distributed data with equal variance from two independent groups; (2) two-tailed unpaired Welch′s *t* test to compare normally distributed data with unequal variance from two independent groups; (3) two-tailed Mann–Whitney *U* test to compare nonnormally distributed data from two independent groups. The statistical information and number of samples for each experiment are indicated in the Figure legends. For detailed statistical results including the test statistics and exact *P* values, see the statistical source data associated with each Figure. All data are shown as the mean ± SEM. Statistical significance was defined as ***P < 0.01; **P < 0.02; *P < 0.05; ns, not significant. All experiments were performed at least three times and reliably reproduced. The investigators were blinded to the experimental groups for each analysis.

## Supplementary information

Appendix

Peer Review File

Movie EV1

Movie EV2

Movie EV3

Movie EV4

Movie EV5

Movie EV6

Movie EV7

Movie EV8

Movie EV9

Movie EV10

Movie EV11

Movie EV12

Movie EV13

Source data Fig. 1

Source data Fig. 2

Source data Fig. 3

Source data Fig. 4

Source data Fig. 5

Figure Source Data for Appendix Fig. S1

Figure Source Data for Appendix Fig. S2

Figure Source Data for Appendix Fig. S3

Figure Source Data for Appendix Fig. S4

Figure Source Data for Appendix Fig. S5

Figure Source Data for Appendix Fig. S6

Figure Source Data for Appendix Fig. S7

## Data Availability

The source data of this paper have been deposited at Mendeley Data (https://data.mendeley.com/datasets/ymmcbh92v6/3) and are publicly available as of the date of publication. The MATLAB codes used for mathematical modeling are available at GitHub (https://github.com/KioYagami/actin-wave/tree/master). The source data of this paper are collected in the following database record: biostudies:S-SCDT-10_1038-S44319-026-00804-6.
