# Supplementary material for: Spontaneous membrane protrusion and cell morphogenesis via self-propelled actin filaments
Source: EMBO Rep. 2026 Jun 25;27(14):3964–81. doi: 10.1038/s44319-026-00804-6 (PMC13400641; doi:10.1038/s44319-026-00804-6)
Supplement: Supplementary file 1 — Appendix [file 44319_2026_804_MOESM1_ESM.pdf]

Appendix for

Spontaneous membrane protrusion and cell morphogenesis via self-propelled actin filaments

Table of content:

Appendix Results and Discussion (page 2)

Appendix Figure S1 (page 3)

Appendix Figure S2 (page 4)

Appendix Figure S3 (page 6)

Appendix Figure S4 (page 8)

Appendix Figure S5 (page 9)

Appendix Figure S6 (page 11)

Appendix Figure S7 (page 13)

Appendix Results and Discussion

This section describes additional properties of SpTA motility that were not covered in the main text.

1) In addition to the SpTA translocations that were altered by the plasma membrane, we observed those that were hampered by other structures. For example, appendix Fig. S7A shows that the direction of SpTA translocation (arrowheads) changed when it collided with other actin filaments enriched in the leading edge (arrow).
2) Neither the de novo formation nor the lifetime of SpTAs were significantly altered by the drug treatment (Cytochalasin B, CK666, SMIFH2) or VASP knockdown (appendix Fig. S7B and C). The cells before and after the drug treatment are shown in Appendix Fig. S7D.
3) The Arp2/3 complex inhibitor CK666 reduced the translocation velocity and length of filopodium-type SpTAs (Fig. S3B and C). Although it is unclear how CK666 reduces the translocation velocity of filopodium-type SpTA, consistent with our notion that filopodium-type and SpTAs serve as precursors of filopodia, previous studies reported that CK666 and ARP2/3 knockdown also inhibit filopodia length (He et al, 2017; Sepúlveda-Ramírez et al, 2018).
4) Fascin localized in filopodium-type SpTAs (Appendix Fig. S1D) is also a constituent of microspikes (Vignjevic et al, 2006), namely F-actin bundles embedded in lamellipodia. Furthermore, the arrival of SpTAs at the cell periphery pushes the membrane to form and expand lamellipodia (Fig. 3B and C). These data suggest that filopodium-type SpTAs also serve as precursors of microspikes.

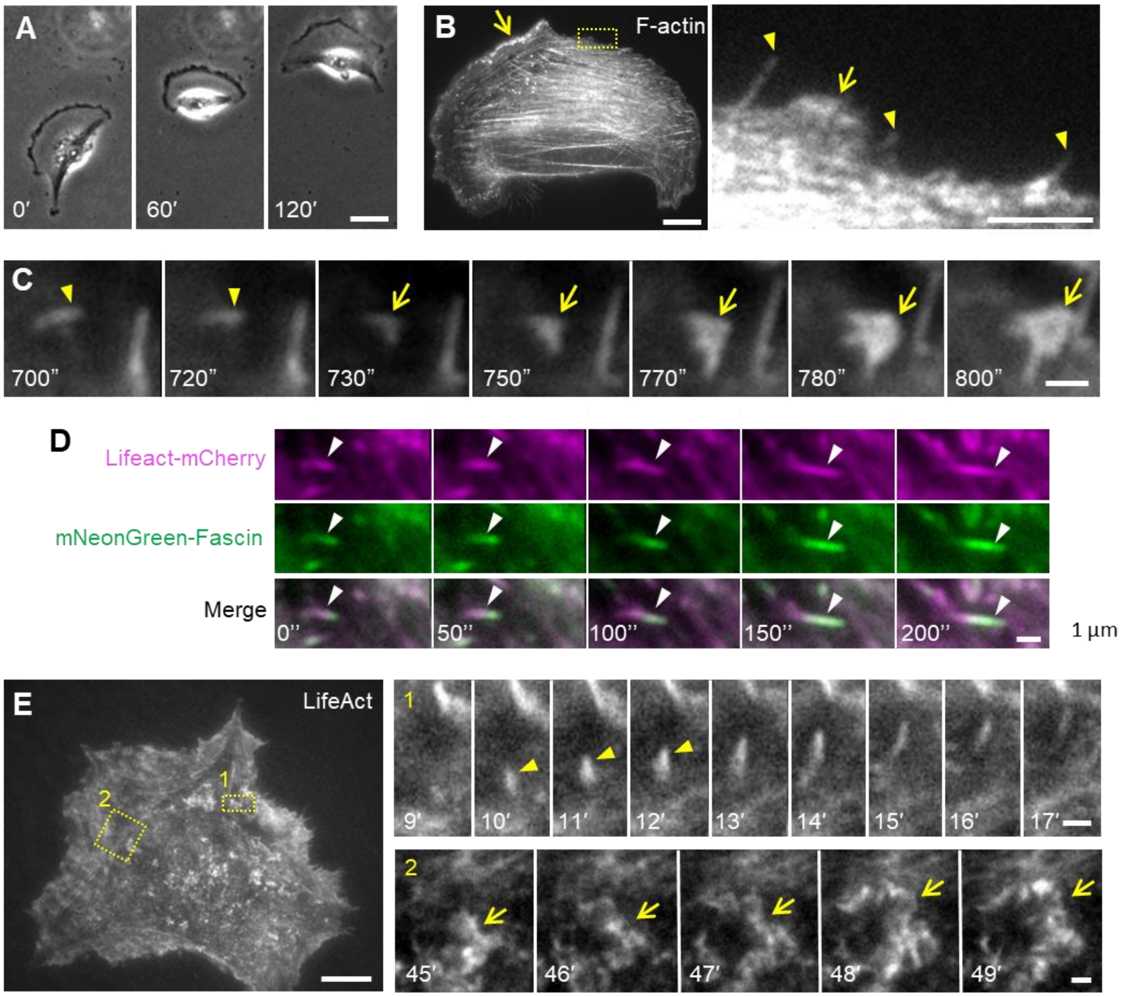

Appendix Figure S1. F-actin assemblies translocate widely in cells.

(A) Time-lapse phase-contrast images of a U251 cell. Scale bar, 20 μm. (B) A U251 cell stained with Alexa Fluor 594 conjugated phalloidin. An enlarged view of the rectangular region is shown to the right. Arrows and arrowheads indicate lamellipodia and filopodia, respectively. Scale bars, 20 μm (left); 5 μm (right). (C) Fluorescence time-lapse images of a lamellipodium-type F-actin assembly (arrows) expanded from a filopodium-type F-actin assembly (arrowheads) in a U251 cell in Fig. 1B. See Movie EV1. Scale bar, 2 μm. (D) Fluorescence time-lapse images of Lifeact-mCherry (magenta) and mNeonGreen-fascin (green) obtained by TIRF microscopy. Fascin is localized in the F-actin bundles (arrowhead). (E) A fluorescence image of a COS7 cell expressing LifeAct-mCherry obtained by TIRF microscopy: right panels indicate enlarged time-lapse images in rectangular regions 1 and 2. Arrows and arrowheads indicate F-actin bundles and meshworks, respectively. See Movie EV2. Scale bars, 20 μm (left); 2 μm (right).

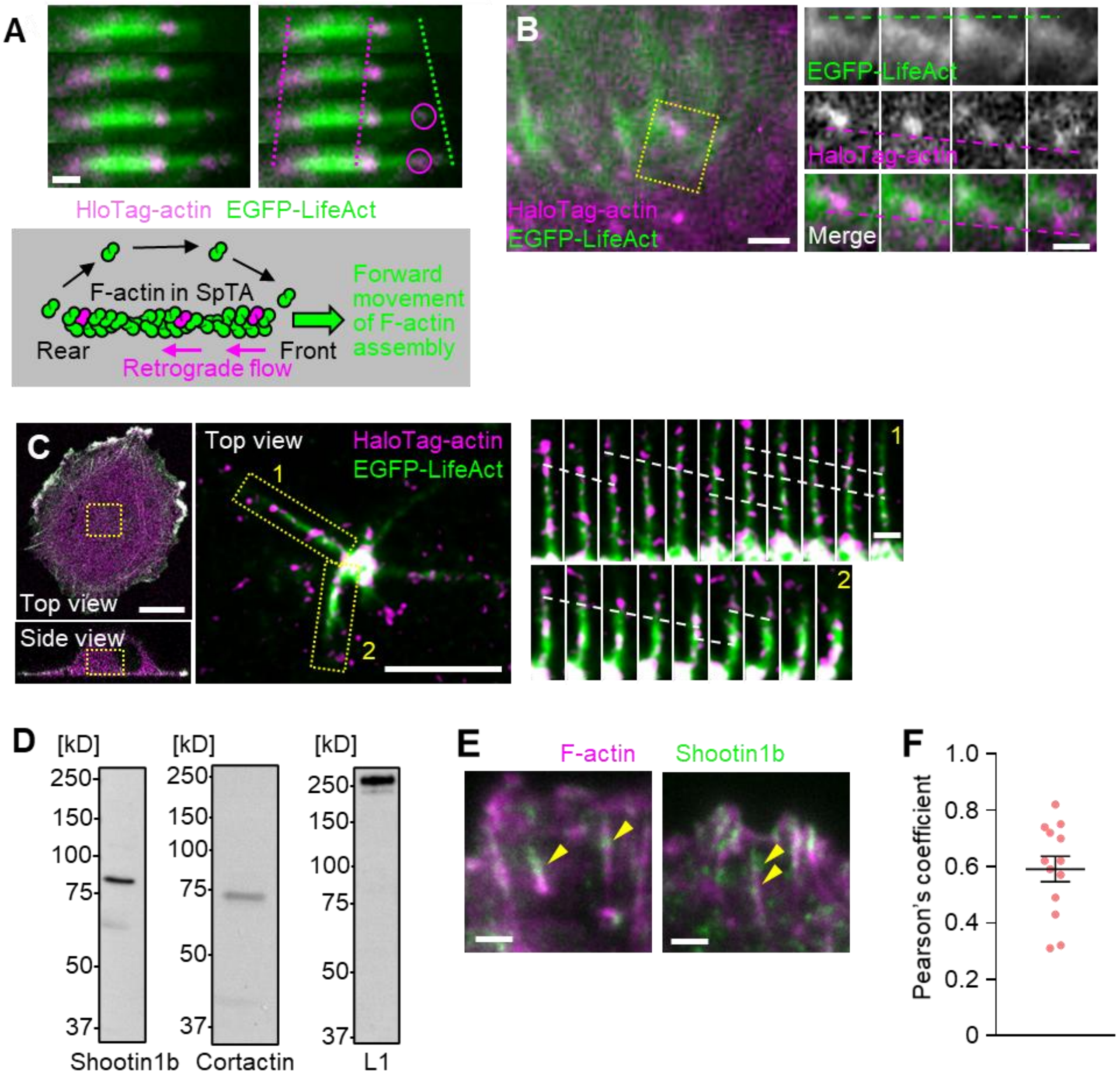

Appendix Figure S2.

(A) Fluorescence time-lapse images of a linear F-actin assembly in a U251 cell expressing LifeAct-mCherry (green) and HaloTag actin (magenta) at 10-sec intervals. The images are identical with (right) and without (left) labelling. F-actin assembly moved forward (green line). HaloTag actin molecules expressed at a low level were incorporated at the front of the F-actin assembly (magenta circles), by polymerization, and underwent retrograde flow (magenta lines). The lower panel shows a diagram describing actin polymerization at the front of F-actin assembly (green arrow) and the retrograde flow of HaloTag actin (magenta arrow) incorporated in SpTA. (B) A fluorescent speckle image of HaloTag-actin in a U251 cell obtained by TIRF microscopy; actin filaments were also monitored by EGFP-LifeAct. Time-lapse montages of a filopodium-type F-actin assembly in the rectangular region at 10-sec intervals are shown to the right. Green and magenta lines indicate the F-actin front and F-actin retrograde flow, respectively. Scale bar, 1 µm. (C) A fluorescent speckle image of HaloTag-actin in a U251 cell obtained by 3D imaging with confocal deconvolution microscopy; actin filaments were also monitored by EGFP-LifeAct. Time-lapse montages of a linear F-actin assembly in the rectangular regions at 10-sec intervals are shown to the right. Dotted

lines indicate F-actin retrograde flow. Scale bars, 20 µm (left); 5 µm (middle); 1 µm (right). (D) Immunoblot analyses of U251 cells with anti-shootin1b, anti-cortactin and anti-L1 antibodies. (E) Additional data of Fig. 2C. Fluorescence images of U251 cells stained with anti-shootin1b antibody (green) and Alexa Fluor 594 conjugated phalloidin (for actin filament, magenta) obtained by TIRF microscopy. Scale bars, 5 µm. (F) Quantification of shootin1b-F-actin colocalization using Pearson's correlation coefficient. The correlation coefficient exceeded 0.5 (0.59 ± 0.05), indicating colocalization of shootin1b and F-actin. N = 3 experiments, n = 12 cells. p = 0.0431. Data represent means ± SEM.

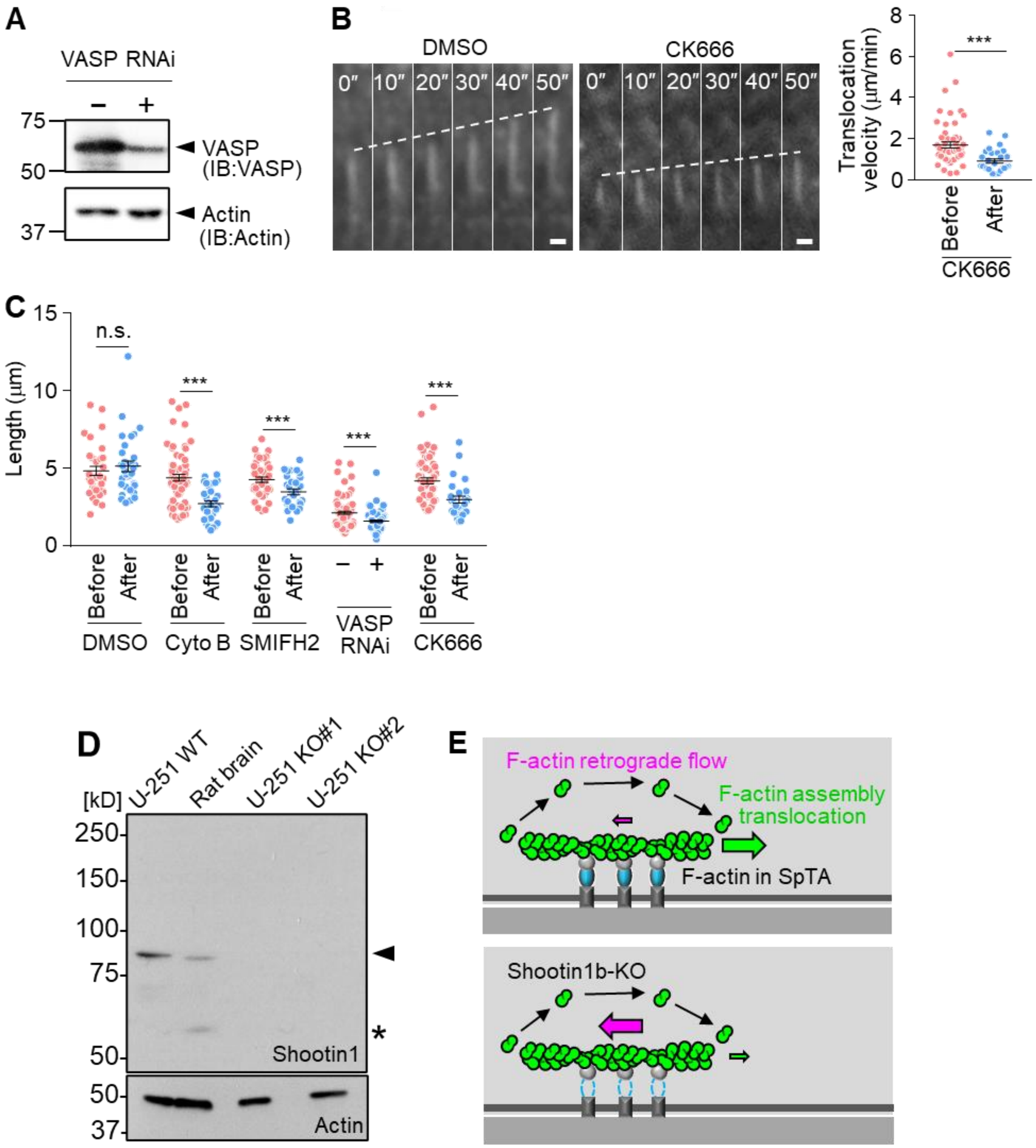

Appendix Figure S3.

(A) Immunoblot analysis of VASP in U251 cells expressing control vector (VASP RNAi -) or VASP knockdown vector (VASP RNAi +). Actin was used as a loading control. (B) Fluorescence time-lapse images of F-actin assemblies in U251 cells after the application of DMSO or 100 µM CK666. Right graph shows the translocation velocities of linear F-actin assemblies before and 10-30 mim after the application of 100 µM CK666 (N = 5 experiments; n = 7 cells; before application, n = 56 F-actin assemblies; after application, n = 28 F-actin assemblies; $p = 6.45 \times 10^{-5}$). (C) Length of linear F-actin assemblies before and 10-30 mim after the application of DMSO (control, N = 3 experiments; n = 3 cells; before application, n = 34 F-actin assemblies; after application, n = 32 F-actin assemblies), cytochalasin B (N = 5 experiments; n = 10 cells; before application,

n = 66 F-actin assemblies; after application, n = 32 F-actin assemblies), SMIFH2 (N = 4 experiments; n = 8 cells; before application, n = 73 F-actin assemblies; after application, n = 31 F-actin assemblies), CK666 (N = 7 cells; before application, n = 56 F-actin assemblies; after application, n = 28 F-actin assemblies) in U251 cells, and in U251 cells expressing control vector (VASP RNAi -, N = 3 experiments; n = 11 cells, n = 90 F-actin assemblies) and VASP knockdown vector (VASP RNAi +, N = 3 experiments; n = 11 cells, n = 81 F-actin assemblies). $p = 0.616$ (DMSO); $p = 8.9 \times 10^{-7}$ (cytochalasin B); $p = 0.00549$ (SMIFH); $p = 9.92 \times 10^{-6}$ (VASP RNAi); $p = 3.27 \times 10^{-5}$ (CK666). (D) Immunoblot analysis of shootin1b in WT U251 cells, postnatal day 1 rat brain, and shootin1b KO U251 cells (KO#1 and KO#2). The cell and tissue lysates were immunoblotted with anti-shootin1 and anti-actin antibodies. The arrowhead and asterisk indicate the bands corresponding to shootin1b and shootin1a, respectively. Shootin1a is a splicing variant of shootin1b expressed in neurons. Immunoblot with anti-actin antibody served as a loading control. (E) A diagram explaining the effects of shootin1b KO on the velocities of actin filament retrograde flow and F-actin assembly translocation. If actin filaments in F-actin assemblies are anchored to the plasma membrane and adhesive substrate through shootin1b (blue), this anchoring impedes the retrograde flow of treadmilling actin filaments. Therefore, shootin1b KO reduces the impedance on the flow, resulting in an increase in the flow velocity (magenta arrow). This in turn reduces F-actin assembly translocation velocity (green arrow). Actin polymerization rate is not affected by shootin1b KO (Fig. 2F). Data represent means ± SEM. ***$p < 0.01$; ns, not significant. Statistical analyses were performed using the two-tailed Mann–Whitney *U*-test (B and C).

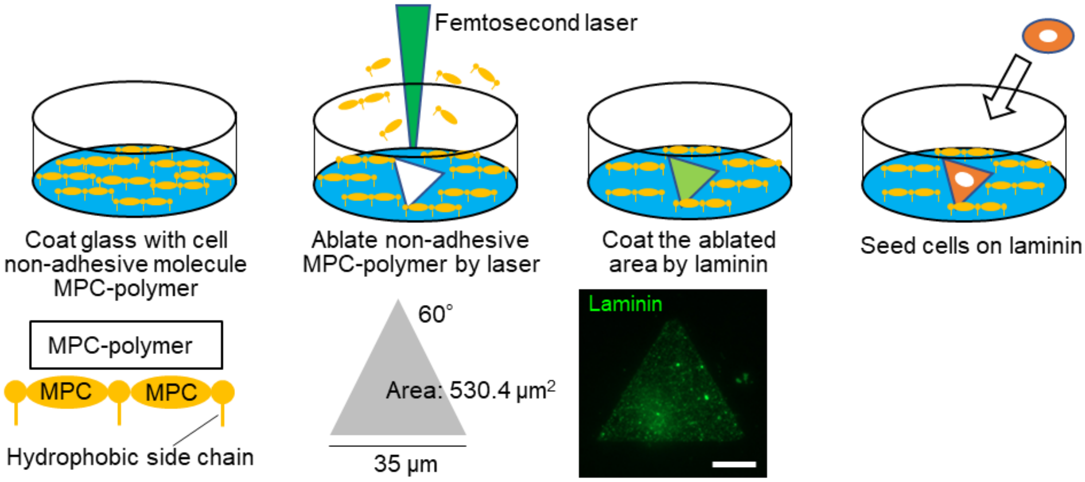

Appendix Figure S4. Preparation of a laminin-coated adhesive island.

A triangular pattern of adhesive laminin substrate with the edge length 35 µm and the area 530.4 μm^2 was created. The cell-adhesive patterns generated were confirmed by coating with green fluorescent-HiLyte 488-Laminin (for details see Materials and Methods). Scale bar 10 µm.

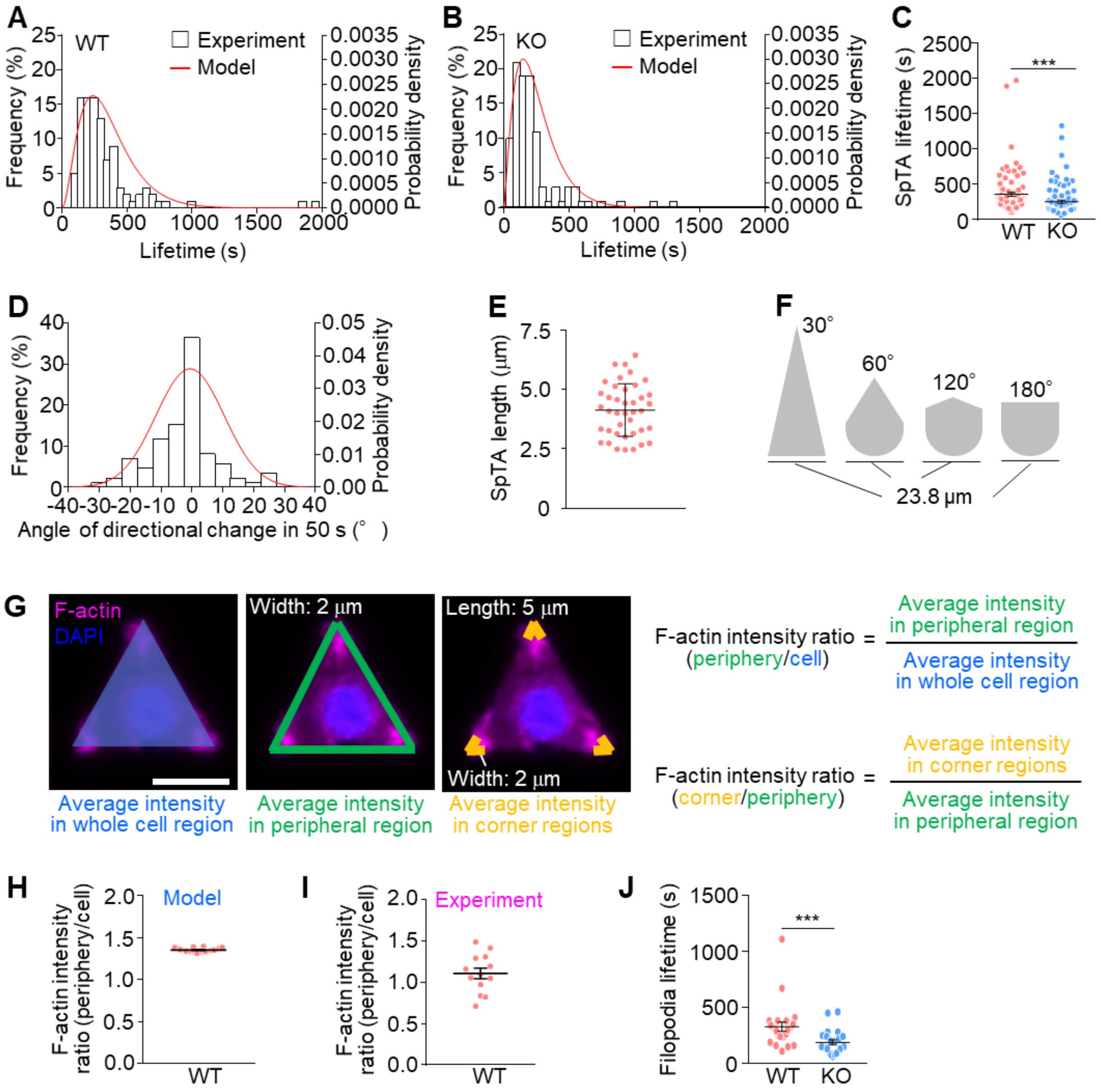

Appendix Figure S5.

(A and B) Distribution of the lifetime of filopodium-type SpTAs in WT (A) and shootin1b KO#1 U251 cells (B). White bars indicate the experimental data, while red curves show gamma distributions with order $\boldsymbol{\alpha}$ = 3.0 and $\boldsymbol{\beta}$ = 119 (A; WT), and with $\boldsymbol{\alpha}$ = 2.4 and $\boldsymbol{\beta}$ = 106 (B; shootin1b KO#1). The parameters were chosen to give the best fit to the experimental data by maximum likelihood estimation. WT and KO#1, N = 5 experiments; n = 9 cells, n = 100 SpTAs. (C) Lifetime of filopodium-type SpTAs in WT and shootin1b KO#1 U251 cells. WT and KO#1, N = 5 experiments; n = 9 cells, n = 100 SpTAs. $p < 0.0001$. (D) Distribution of the degree of the change in travelling direction of filopodium-type SpTAs in 50 sec. White bars indicate the experimental data, while red curves show Gaussian distributions with order mean; $\boldsymbol{\mu}$ = -0.61 and SD; $\boldsymbol{\sigma}$ = 11. The parameters were chosen to give the best fit to the experimental data by maximum likelihood estimation (N = 3 experiments; N = 8 cells, n = 86 SpTAs). (E) Length of filopodium-type SpTAs (N = 3 experiments; n = 4 cells, n = 40 SpTAs). (F) The pattern of adhesive laminin substrate with different corner angles, 30º, 60º, 120º or 180º, and the same width 23.8 μm and the same area 530.4

μm^2 used in the experiments in Fig. 4G. (G) The accumulation of actin filaments at the cell periphery was determined by dividing the average actin intensity in the peripheral region (green region; 2 μm width) by the average actin intensity in the whole cell region (blue region). Similarly, the accumulation at corners was determined by dividing the average of actin intensity in the corner regions (yellow regions; 2 μm width, 5 μm length) by the average actin intensity in the peripheral region (green region). Scale bar, 20 μm. (H and I) The accumulation rates of actin filaments at cell periphery in model data (H, N = 3 experiments; n = 20 model data) and U251 cells (N = 3 experiments; n = 13 cells). (J) Lifetime of filopodia in WT (N = 7 cells; n = 24 SpTAs) and shootin1b KO#1 (N= 3 experiments; n = 5 cells; n = 23 SpTAs) U251 cells. p = 0.005. Data represent means ± SEM. ***p < 0.01. Statistical analysis was performed using the two-tailed Mann–Whitney *U*-test (C and J).

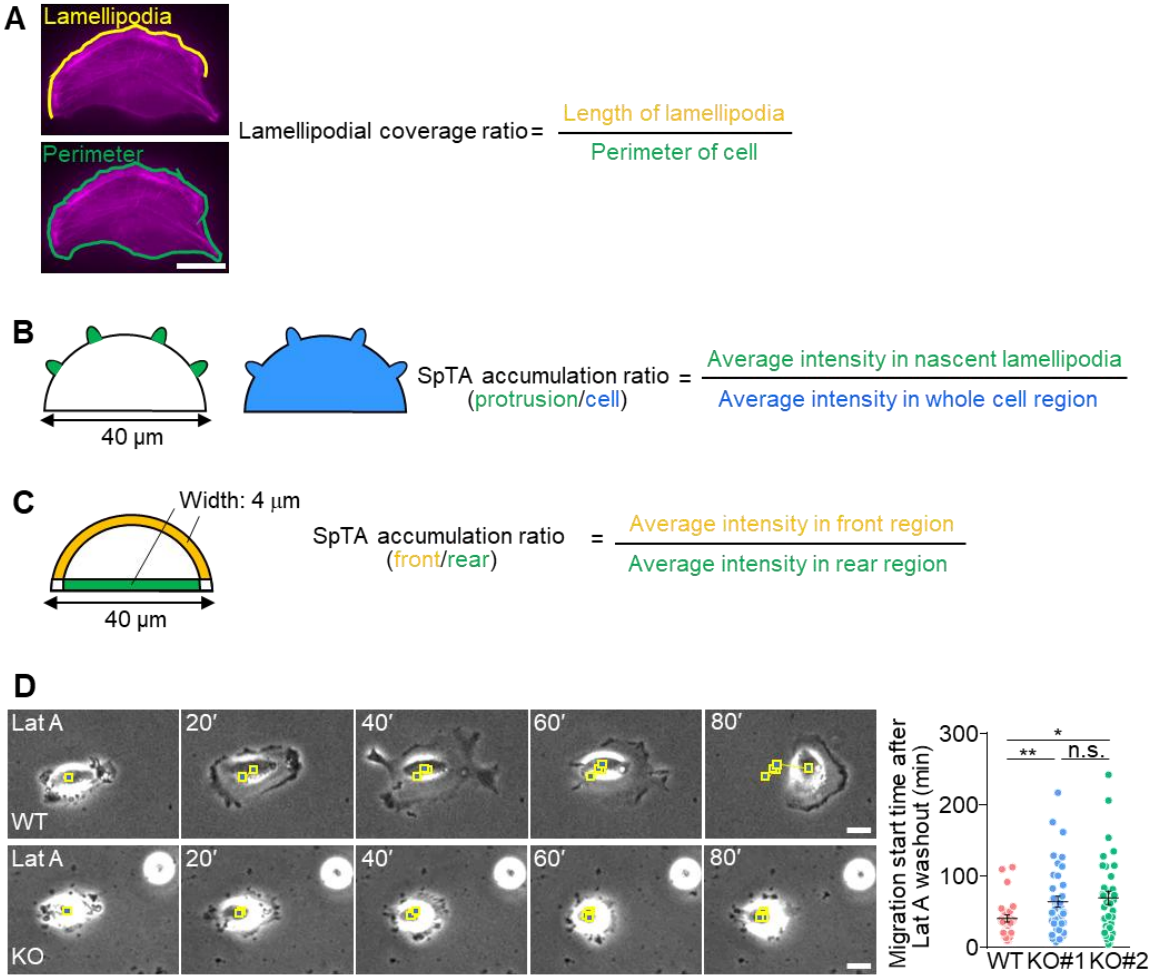

Appendix Figure S6.

(A) The lamellipodial coverage rate was determined by dividing the total length of lamellipodia (yellow line) by the perimeter of the cell (green line). Scale bar, 20 µm. The cell image is the reuse of Fig. 5C. (B) The accumulation of actin filaments at the nascent lamellipodia was determined by dividing the average fluorescence intensity of actin filaments in protrusive regions (green regions) by the average intensity of actin filaments in the whole cell region (blue region). (C) Accumulation of actin filaments at the integrated lamellipodium was determined by dividing the average intensity of actin filaments at the convex leading edge (yellow region; 4 µm-width) by the average intensity of actin filaments in the rear region (green region; 4 µm-width). (D) Time-lapse phase-contrast images of WT and shootin1b KO#1 U251 cells taken after the treatment with 100 nM Lat A and at the indicated times after the Lat A washout. Yellow boxes trace cell movement. The right graph indicates the time required to start cell migration after the Lat A washout. WT, N = 3 experiments, n = 28 cells; KO#1, N = 3 experiments, n = 40 cells; KO#2, N = 3 experiments, n = 35 cells. Scale bar, 20 µm. $p = 0.017$ (WT vs KO#1); $p = 0.039$ (WT vs KO#2); $p = 0.762$ (KO#1 vs KO#2). Data represent means ± SEM; **$p < 0.02$; *$p < 0.05$; ns, not significant. Statistical analyses were performed using the two-tailed Mann–Whitney *U*-test.

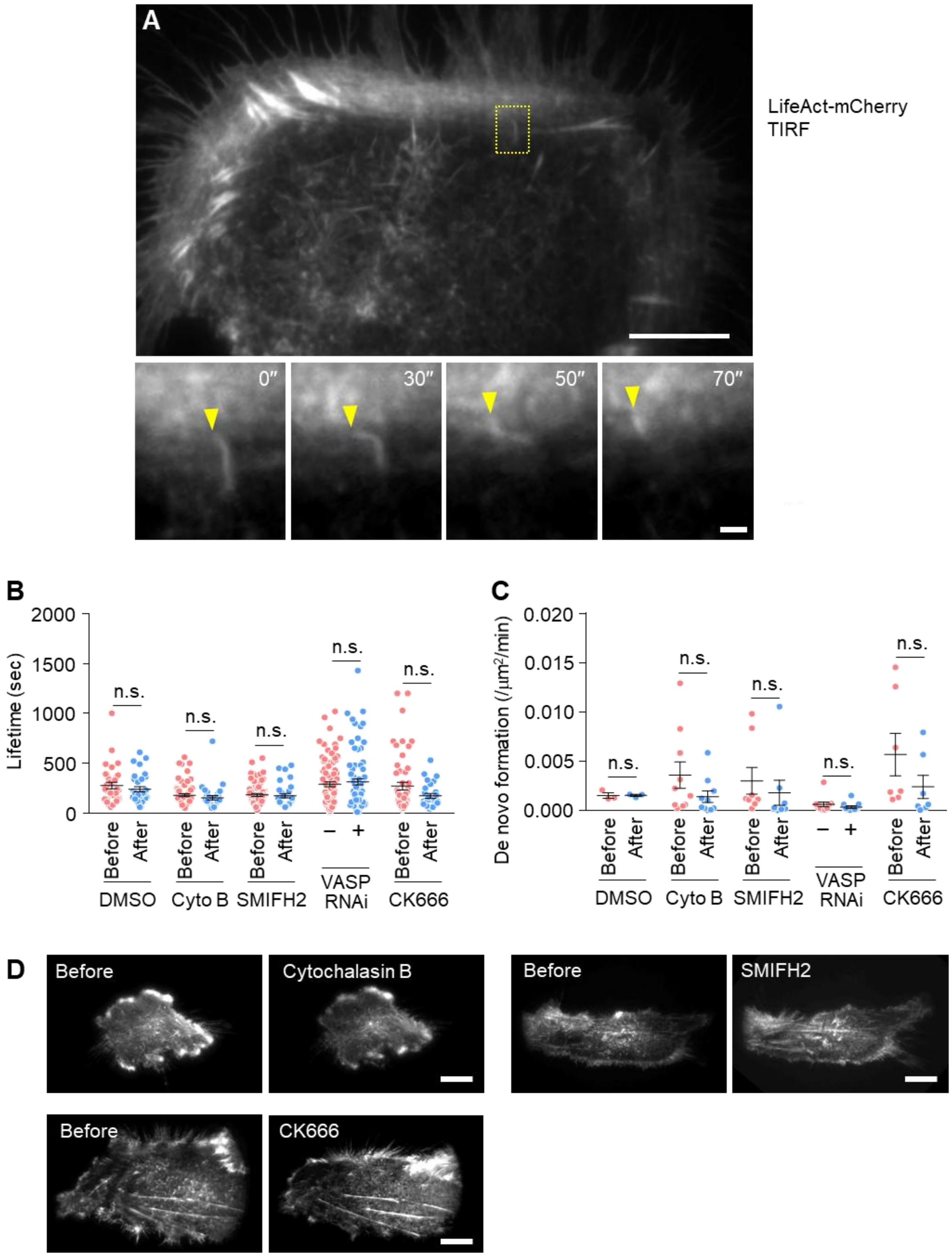

Appendix Figure S7.
(A) Fluorescence time-lapse images of filopodium-type SpTA in a U251 cell expressing LifeAct-mCherry obtained by TIRF microscopy. The lower panels indicate enlarged time-lapse images of the areas indicated by the yellow rectangle. Scale Bars, 20 μm (upper); 2 μm (lower). (B and C)

Lifetime (B) and de novo formation (C) of filopodium-type SPTAs before and 10-30 mim after the application of DMSO (DMSO), cytochalasin B, SMIFH2, CK666, in U251 cells, and in U251 cells expressing control vector (VASP RNAi -) and VASP knockdown vector (VASP RNAi +). DMSO in (B) (N = 3 experiments; n = 3 cells: before application, n = 34 F-actin assemblies; after application, n = 32 F-actin assemblies); cytochalasin B in (B) (N = 5 experiments; n = 10 cells: before application, n = 66 F-actin assemblies; after application, n = 32 F-actin assemblies, SMIFH2 in (B) (N = 4 experiments; n = 8 cells: before application, n = 73 F-actin assemblies; after application, n = 31 F-actin assemblies), VASP RNAi – in (B) (N = 3 experiments; n = 11 cells: n = 90 F-actin assemblies); VASP RNAi + in (B) (N = 3 experiments, n = 11 cells: n = 81 F-actin assemblies); CK666 in (B) (N = 5 experiments; n = 7 cells: before application, n = 56 F-actin assemblies; after application, n = 28 F-actin assemblies); DMSO in (C) (N = 3 experiments: n = 3 cells); cytochalasin B in (C) (N = 5 experiments: n = 10 cells); SMIFH2 in (C) (N = 4 experiments: n = 8 cells); VASP RNAi – in (C) (N = 3 experiments: n = 11 cells); VASP RNAi + in (C) (N = N = 3 experiments: n = 11 cells); CK666 in (C) (N = 4 experiments: 7 cells). DMSO in (B), p = 0.493; cytochalasin B in (B), p = 0.251; SMIFH2 in (B), p = 0.525; VASP RNAi in (B), p = 0.704; CK666 in (B); p = 0.295; DMSO in (C), p = 0.493; cytochalasin B in (B), p = 0.251; SMIFH2 in (B), p = 0.525; VASP RNAi in (B), p = 0.704; CK666 in (B); p = 0.295; DMSO in (C), p = 0.700; cytochalasin B in (C), p = 0.143; SMIFH2 in (C), p = 0.130; VASP RNAi in (C), p = 0.193; CK666 in (C); p = 0.0973. (D) Fluorescence images of U251 cells expressing LifeAct-mCherry before and after the application of 0.05 µM cytochalasin B, 5 µM SMIFH2, and 100 µM CK666. Images were obtained by TIRF microscopy. Scale Bars, 20 µm. Data represent means ± SEM. n.s., not significant. Statistical analyses were performed using the two-tailed Mann–Whitney *U*-test (B and C).
