# Supplementary material for: Spontaneous membrane protrusion and cell morphogenesis via self-propelled actin filaments
Source: EMBO Rep. 2026 Jun 25;27(14):3964–81. doi: 10.1038/s44319-026-00804-6 (PMC13400641; doi:10.1038/s44319-026-00804-6)
Supplement: Supplementary file 3 — Movie EV1 [file 44319_2026_804_MOESM3_ESM.zip › Movie EV1/Movie EV1 legend.docx]

**Movie EV1**

U251 cells expressing EGFP-LifeAct observed by TIRF microscopy. Left: linear F-actin bundles and F-actin meshworks emerged widely in the cytoplasm, moved in random directions, and disappeared (see Fig. 1A). Right: linear F-actin bundles changed the direction of their translocation randomly (yellow arrowheads); some emerged and separated (yellow and cyan arrowheads) from F-actin meshworks (arrow) or branched off (cyan arrowheads) from F-actin bundles (see Fig. 1B). Some and F-actin meshworks expanded from the filopodium-type assemblies (arrow, see Appendix Fig. S1C). Time interval: 10 sec. Scale bar: 20 µm (left), 10 µm (right).
