# Supplementary material for: Spontaneous membrane protrusion and cell morphogenesis via self-propelled actin filaments
Source: EMBO Rep. 2026 Jun 25;27(14):3964–81. doi: 10.1038/s44319-026-00804-6 (PMC13400641; doi:10.1038/s44319-026-00804-6)
Supplement: Supplementary file 4 — Movie EV2 [file 44319_2026_804_MOESM4_ESM.zip › Movie EV2/Movie EV2 legend.docx]

**Movie EV2**

F-actin assemblies moving on the ventral plasma membrane of a COS7 cell expressing LifeAct-mCherry observed by TIRF microscopy (see Appendix Fig. S1E). Time interval: 60 sec. Scale bar: 20 µm.
