# Supplementary material for: Spontaneous membrane protrusion and cell morphogenesis via self-propelled actin filaments
Source: EMBO Rep. 2026 Jun 25;27(14):3964–81. doi: 10.1038/s44319-026-00804-6 (PMC13400641; doi:10.1038/s44319-026-00804-6)
Supplement: Supplementary file 5 — Movie EV3 [file 44319_2026_804_MOESM5_ESM.zip › Movie EV3/Movie EV3 legend.docx]

**Movie EV3**

An F-actin assembly translocating through the cytoplasm without interacting with the plasma membrane (see Fig. 1D). A U251 cell expressing LifeAct-mCherry was observed by 3D imaging with confocal deconvolution microscopy. Time interval: 13 sec. Z-stack interval: 0.2 µm. Scale bar: 5 µm.
