# Supplementary material for: Spontaneous membrane protrusion and cell morphogenesis via self-propelled actin filaments
Source: EMBO Rep. 2026 Jun 25;27(14):3964–81. doi: 10.1038/s44319-026-00804-6 (PMC13400641; doi:10.1038/s44319-026-00804-6)
Supplement: Supplementary file 6 — Movie EV4 [file 44319_2026_804_MOESM6_ESM.zip › Movie EV4/Movie EV4 legend.docx]

**Movie EV4**

A U251 cell expressing LifeAct-mCherry observed by TIRF microscopy and EGFP-LifeAct observed by epifluorescence microscopy (see Fig. 1E and F). F-actin assemblies observed by epifluorescence microscopy and TIRF microscopy are indicated by green and magenta arrowheads, respectively. Time interval: 60 sec.
