# Supplementary material for: Spontaneous membrane protrusion and cell morphogenesis via self-propelled actin filaments
Source: EMBO Rep. 2026 Jun 25;27(14):3964–81. doi: 10.1038/s44319-026-00804-6 (PMC13400641; doi:10.1038/s44319-026-00804-6)
Supplement: Supplementary file 7 — Movie EV5 [file 44319_2026_804_MOESM7_ESM.zip › Movie EV5/Movie EV5 legend.docx]

**Movie EV5**

Fluorescent speckle dynamics of HaloTag-actin in an F-actin assembly; actin filaments were also monitored by EGFP-LifeAct. They were observed by TIRF microscopy. The F-actin assembly underwent directional assembly/disassembly and translocated in the direction of polymerization (see Fig. 2A). A Time interval: 10 sec. Scale bar: 1 µm.
