# Supplementary material for: Spontaneous membrane protrusion and cell morphogenesis via self-propelled actin filaments
Source: EMBO Rep. 2026 Jun 25;27(14):3964–81. doi: 10.1038/s44319-026-00804-6 (PMC13400641; doi:10.1038/s44319-026-00804-6)
Supplement: Supplementary file 8 — Movie EV6 [file 44319_2026_804_MOESM8_ESM.zip › Movie EV6/Movie EV6 legend.docx]

**Movie EV6**

Filopodia formation by linear F-actin bundles (arrowheads) (see Fig. 3A). A U251 cell expressing EGFP-LifeAct was observed by epifluorescence microscopy. Time interval: 10 sec. Scale bar: 5 µm.
