# Supplementary material for: Spontaneous membrane protrusion and cell morphogenesis via self-propelled actin filaments
Source: EMBO Rep. 2026 Jun 25;27(14):3964–81. doi: 10.1038/s44319-026-00804-6 (PMC13400641; doi:10.1038/s44319-026-00804-6)
Supplement: Supplementary file 9 — Movie EV7 [file 44319_2026_804_MOESM9_ESM.zip › Movie EV7/Movie EV7 legend.docx]

**Movie EV7**

Lamellipodium formation by a F-actin meshwork (yellow arrow) (see Fig. 3B). A U251 cell expressing LifeAct-mCherry was observed by TIRF microscopy. The F-actin meshwork merged with the pre-existing lamellipodia (cyan arrow) through lateral movement. Time interval: 60 sec. Scale bar: 5 µm.
