# Supplementary material for: Spontaneous membrane protrusion and cell morphogenesis via self-propelled actin filaments
Source: EMBO Rep. 2026 Jun 25;27(14):3964–81. doi: 10.1038/s44319-026-00804-6 (PMC13400641; doi:10.1038/s44319-026-00804-6)
Supplement: Supplementary file 10 — Movie EV8 [file 44319_2026_804_MOESM10_ESM.zip › Movie EV8/Movie EV8 legend.docx]

**Movie EV8**

Linear F-actin bundles entering into pre-existing lamellipodium (left) and filopodium (asterisk, right). The F-actin assembly entry resulted in local actin filament accumulation and lamellipodial expansion (arrow) (See Fig. 3C). U251 cells expressing EGFP-LifeAct were observed by TIRF microscopy. Time interval: 10 sec. Scale bars: 1 µm.
