# Supplementary material for: Spontaneous membrane protrusion and cell morphogenesis via self-propelled actin filaments
Source: EMBO Rep. 2026 Jun 25;27(14):3964–81. doi: 10.1038/s44319-026-00804-6 (PMC13400641; doi:10.1038/s44319-026-00804-6)
Supplement: Supplementary file 11 — Movie EV9 [file 44319_2026_804_MOESM11_ESM.zip › Movie EV9/Movie EV9 legend.docx]

**Movie EV9**

Linear F-actin bundles travelling along the lateral membranes (see Fig. 3E). U251 cells expressing LifeAct-mCherry were observed by epifluorescence microscopy. Time interval: 10 sec. Scale bars: 5 µm.
