# Supplementary material for: Spontaneous membrane protrusion and cell morphogenesis via self-propelled actin filaments
Source: EMBO Rep. 2026 Jun 25;27(14):3964–81. doi: 10.1038/s44319-026-00804-6 (PMC13400641; doi:10.1038/s44319-026-00804-6)
Supplement: Supplementary file 12 — Movie EV10 [file 44319_2026_804_MOESM12_ESM.zip › Movie EV10/Movie EV10 legend.docx]

**Movie EV10**

Filopodium-type SpTAs (arrowheads) and a lamellipodium-type SpTA (arrow) accumulating at protrusive regions (asterisks) (see Fig. 4A and B). U251 cells expressing EGFP-LifeAct were observed by epifluorescence microscopy. Time interval: 10 sec. Scale bars: 5 µm (left), 20 µm (right).
