# Supplementary material for: Spontaneous membrane protrusion and cell morphogenesis via self-propelled actin filaments
Source: EMBO Rep. 2026 Jun 25;27(14):3964–81. doi: 10.1038/s44319-026-00804-6 (PMC13400641; doi:10.1038/s44319-026-00804-6)
Supplement: Supplementary file 13 — Movie EV11 [file 44319_2026_804_MOESM13_ESM.zip › Movie EV11/Movie EV11 legend.docx]

**Movie EV11**

SpTA accumulation at the corners of a triangular U251 cell (see Fig. 4D). A U251 cell expressing LifeAct-mCherry was cultured on a triangular adhesive island and observed by epifluorescence microscopy. SpTAs travelled along the lateral edge, resulting in local accumulation of actin filaments at the corners upon arrival (arrowheads). Time interval: 300 sec. Scale bar: 10 µm.
