# Supplementary material for: Spontaneous membrane protrusion and cell morphogenesis via self-propelled actin filaments
Source: EMBO Rep. 2026 Jun 25;27(14):3964–81. doi: 10.1038/s44319-026-00804-6 (PMC13400641; doi:10.1038/s44319-026-00804-6)
Supplement: Supplementary file 14 — Movie EV12 [file 44319_2026_804_MOESM14_ESM.zip › Movie EV12/Movie EV12 legend.docx]

**Movie EV12**

Mathematical model data showing SpTA accumulation in WT and shootin1b KO triangular cells (see Fig. 4E and H). Time interval: 10 sec. Scale bars: 5 µm.
