# Supplementary material for: Spontaneous membrane protrusion and cell morphogenesis via self-propelled actin filaments
Source: EMBO Rep. 2026 Jun 25;27(14):3964–81. doi: 10.1038/s44319-026-00804-6 (PMC13400641; doi:10.1038/s44319-026-00804-6)
Supplement: Supplementary file 15 — Movie EV13 [file 44319_2026_804_MOESM15_ESM.zip › Movie EV13/Movie EV13 legend.docx]

**Movie EV13**

Fluorescence time-lapse Videos of WT and shootin1b KO#1 U251 cells expressing LifeAct-mCherry and observed by epifluorescence microscopy (see Fig. 5D and G). The images were taken before and after the treatment with 100 nM latrunculin A, and after the Lat A washout. Time interval: 60 sec. Scale bars: 20 µm.
