# Supplementary figures and images for: Spontaneous membrane protrusion and cell morphogenesis via self-propelled actin filaments
Source: EMBO Rep. 2026 Jun 25;27(14):3964–81. doi: 10.1038/s44319-026-00804-6 (PMC13400641; doi:10.1038/s44319-026-00804-6)

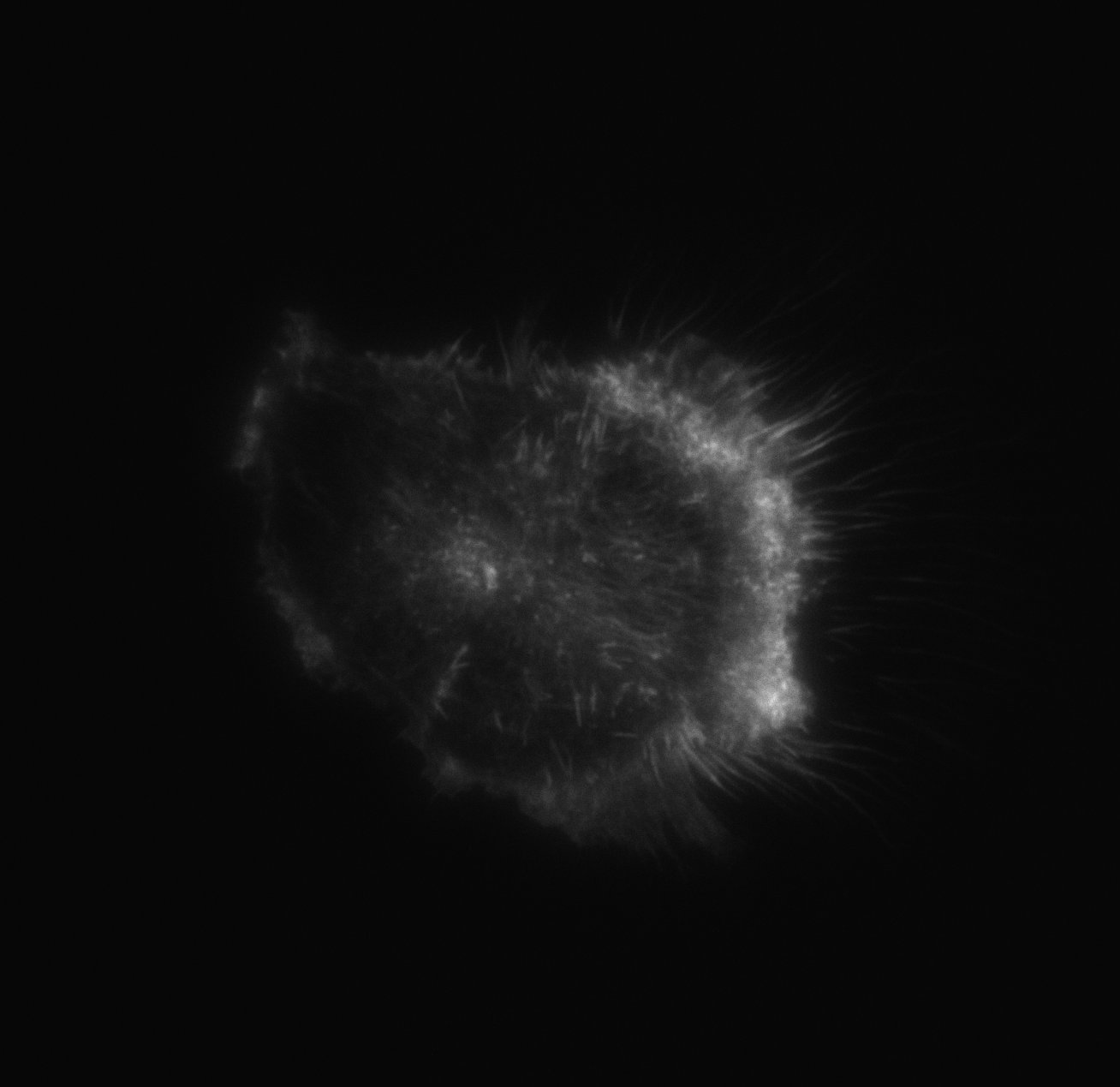

Supplement: Supplementary file 16 — Source data Fig. 1 [file 44319_2026_804_MOESM16_ESM.zip › Fig. 1/1A/Lifeact.tif]

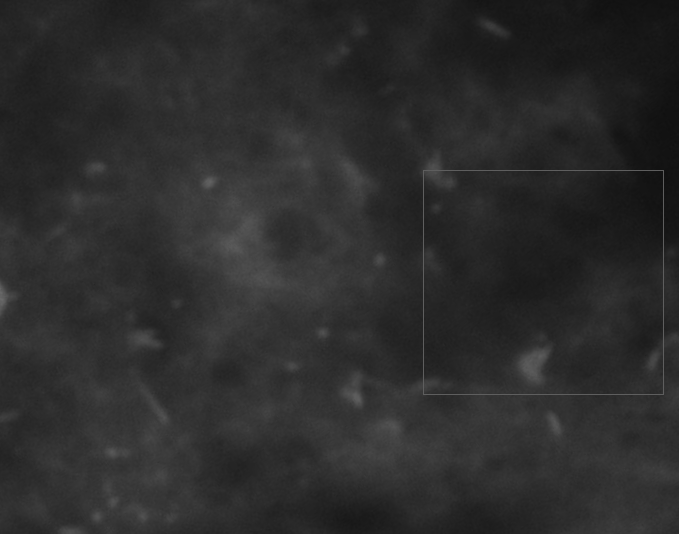

Supplement: Supplementary file 16 — Source data Fig. 1 [file 44319_2026_804_MOESM16_ESM.zip › Fig. 1/1B/Fig1B.tif]

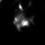

Supplement: Supplementary file 16 — Source data Fig. 1 [file 44319_2026_804_MOESM16_ESM.zip › Fig. 1/1C/Dorsal.tif]

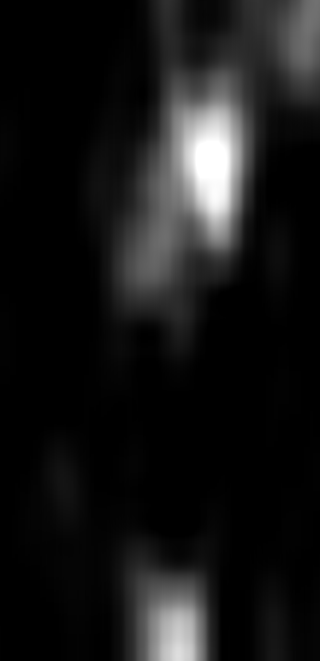

Supplement: Supplementary file 16 — Source data Fig. 1 [file 44319_2026_804_MOESM16_ESM.zip › Fig. 1/1D/Side view.tif]

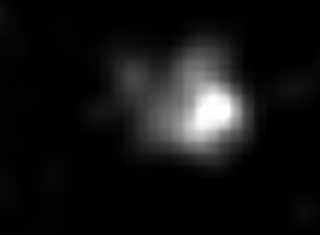

Supplement: Supplementary file 16 — Source data Fig. 1 [file 44319_2026_804_MOESM16_ESM.zip › Fig. 1/1D/Top view.tif]

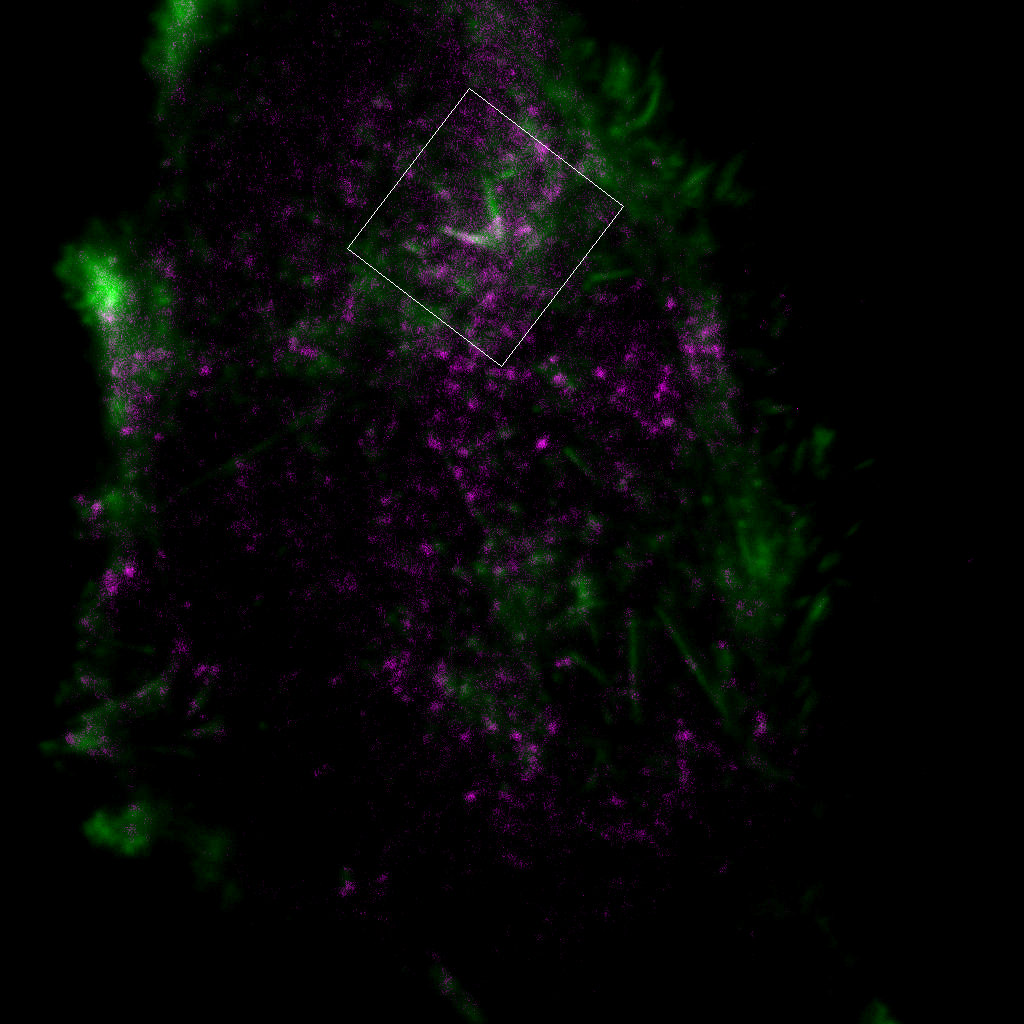

Supplement: Supplementary file 17 — Source data Fig. 2 [file 44319_2026_804_MOESM17_ESM.zip › Fig. 2/2A/Left untrimmed image.tif]

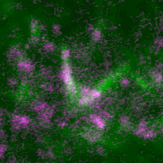

Supplement: Supplementary file 17 — Source data Fig. 2 [file 44319_2026_804_MOESM17_ESM.zip › Fig. 2/2A/Left.tif]

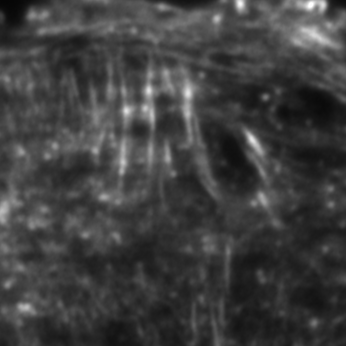

Supplement: Supplementary file 17 — Source data Fig. 2 [file 44319_2026_804_MOESM17_ESM.zip › Fig. 2/2C/F-actin.tif]

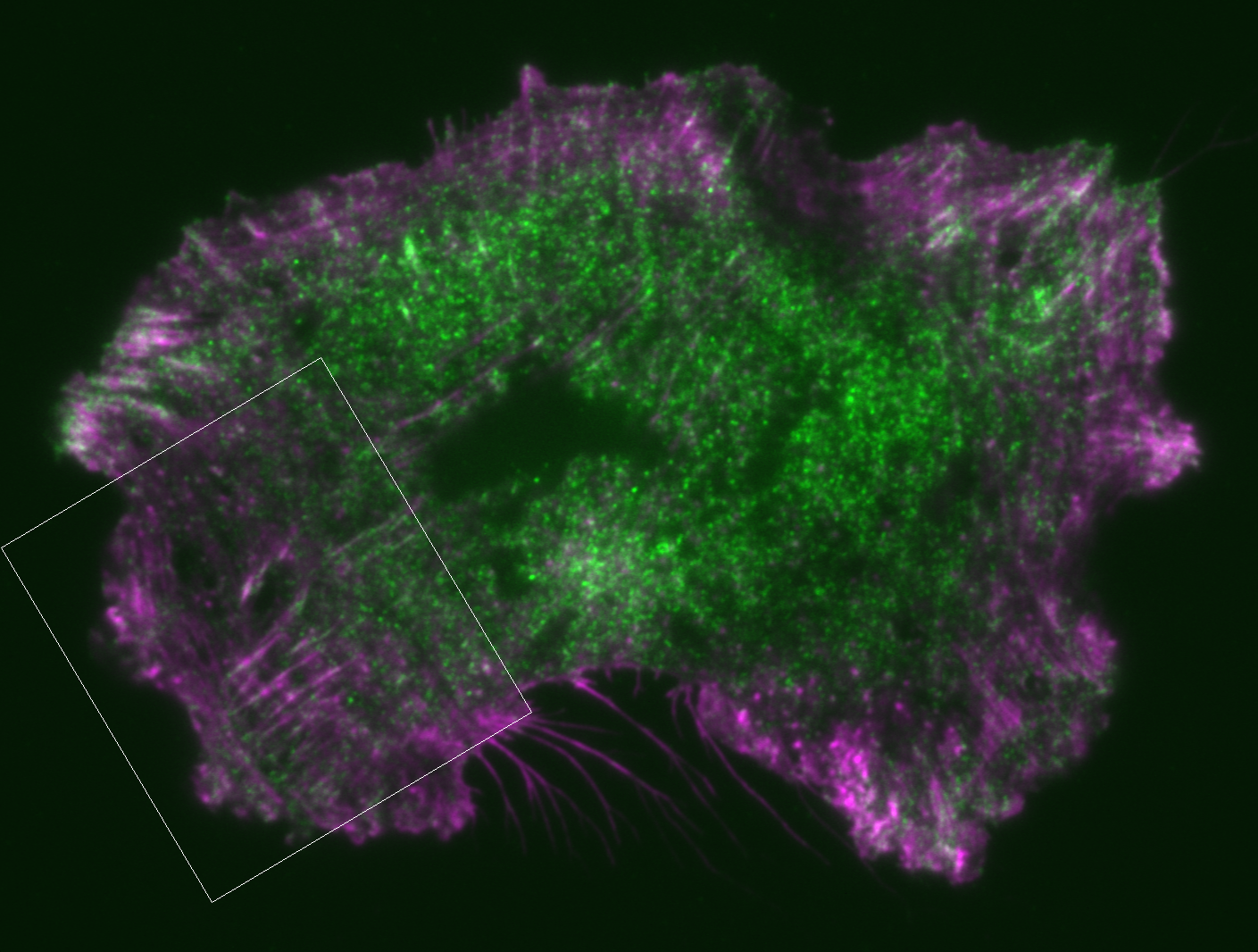

Supplement: Supplementary file 17 — Source data Fig. 2 [file 44319_2026_804_MOESM17_ESM.zip › Fig. 2/2C/Left.tif]

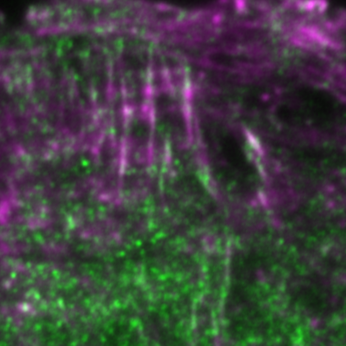

Supplement: Supplementary file 17 — Source data Fig. 2 [file 44319_2026_804_MOESM17_ESM.zip › Fig. 2/2C/Merge.tif]

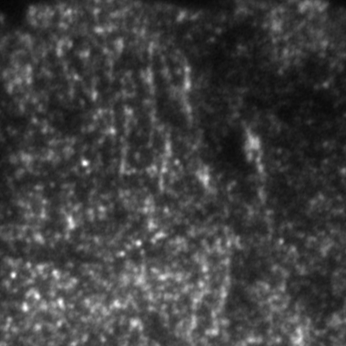

Supplement: Supplementary file 17 — Source data Fig. 2 [file 44319_2026_804_MOESM17_ESM.zip › Fig. 2/2C/Shootin1b.tif]

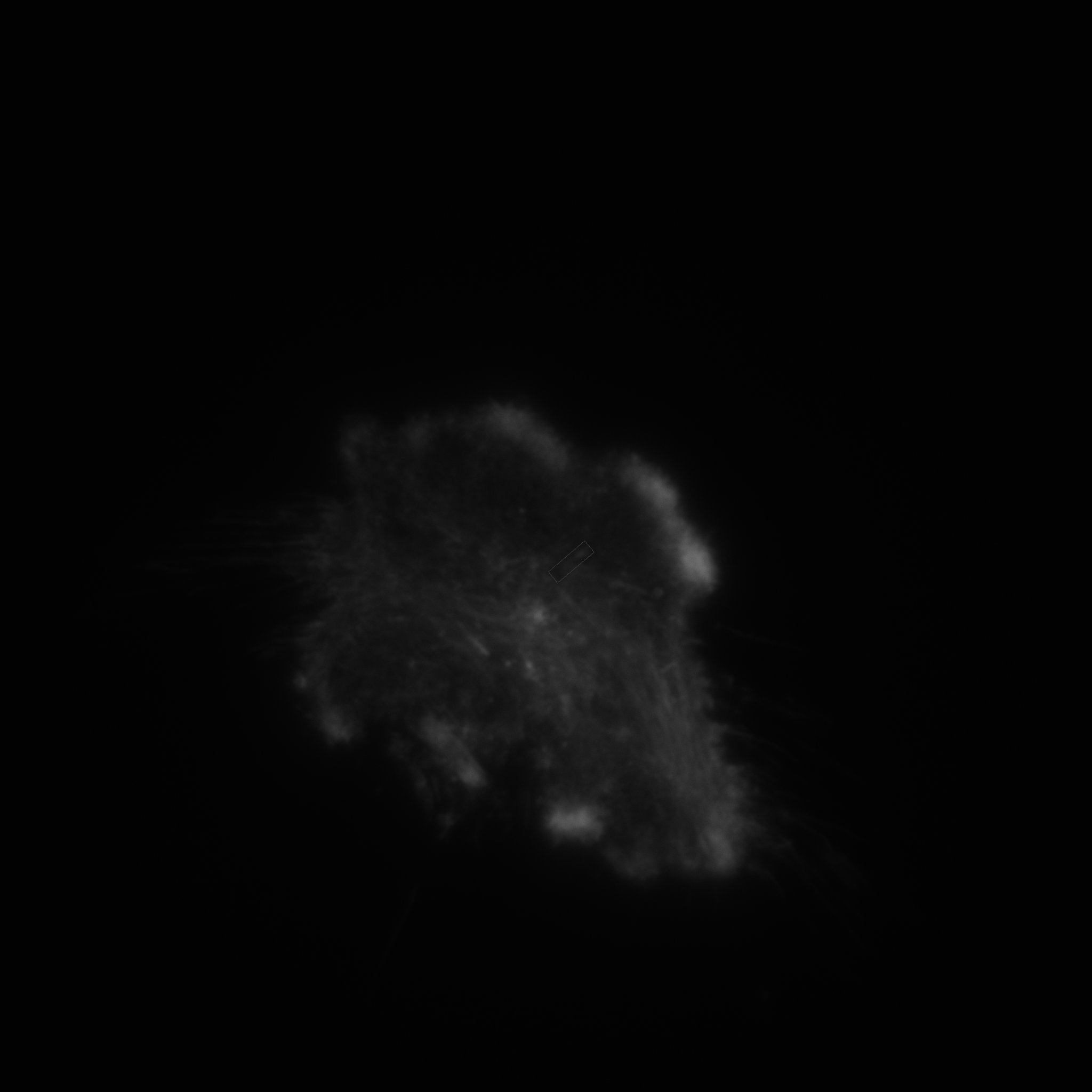

Supplement: Supplementary file 17 — Source data Fig. 2 [file 44319_2026_804_MOESM17_ESM.zip › Fig. 2/2D/Cytochalasin B/CyroB untrimmed image.tif]

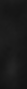

Supplement: Supplementary file 17 — Source data Fig. 2 [file 44319_2026_804_MOESM17_ESM.zip › Fig. 2/2D/Cytochalasin B/Cyto B.tif]

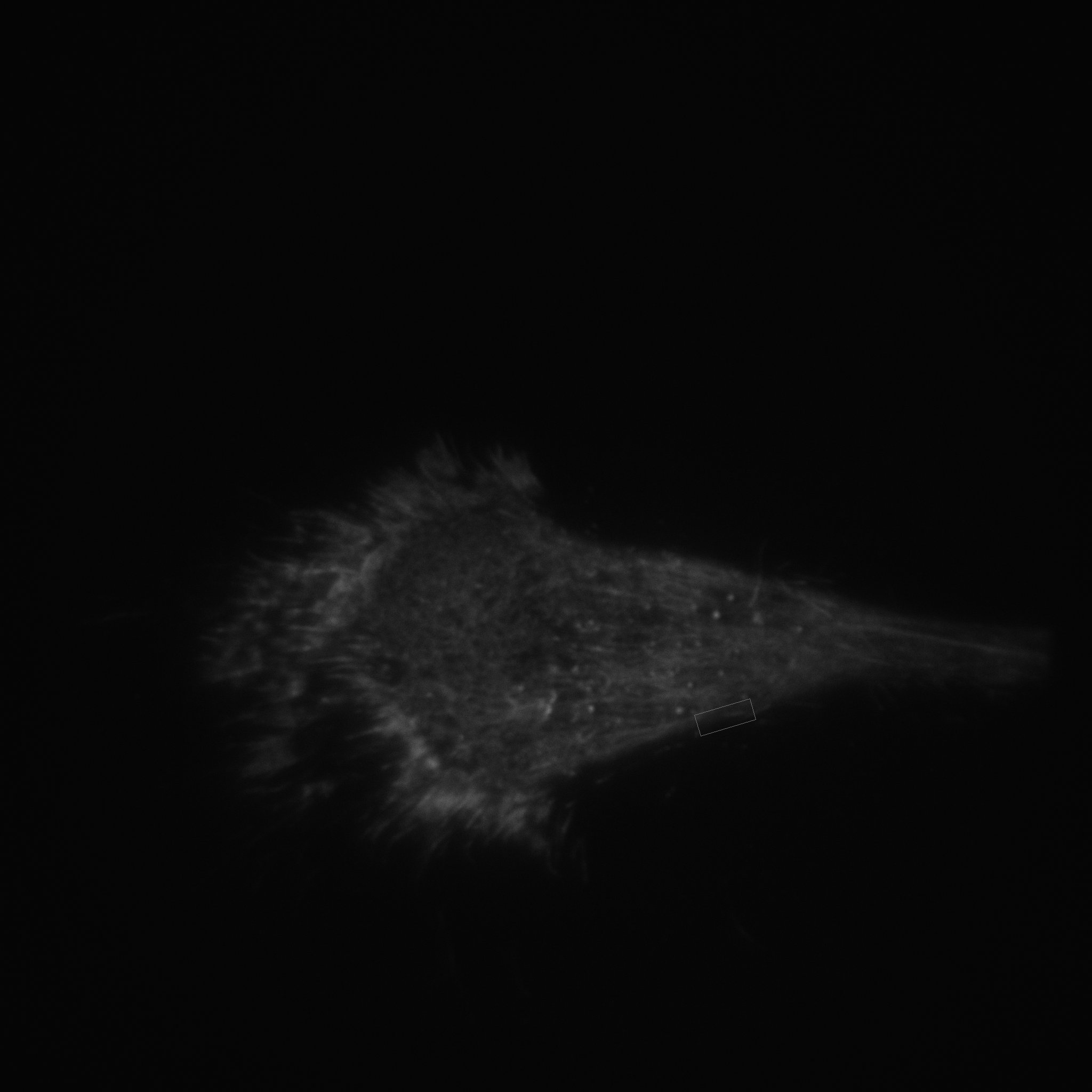

Supplement: Supplementary file 17 — Source data Fig. 2 [file 44319_2026_804_MOESM17_ESM.zip › Fig. 2/2D/DMSO/DMSO untrimmed image.tif]

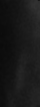

Supplement: Supplementary file 17 — Source data Fig. 2 [file 44319_2026_804_MOESM17_ESM.zip › Fig. 2/2D/DMSO/DMSO.tif]

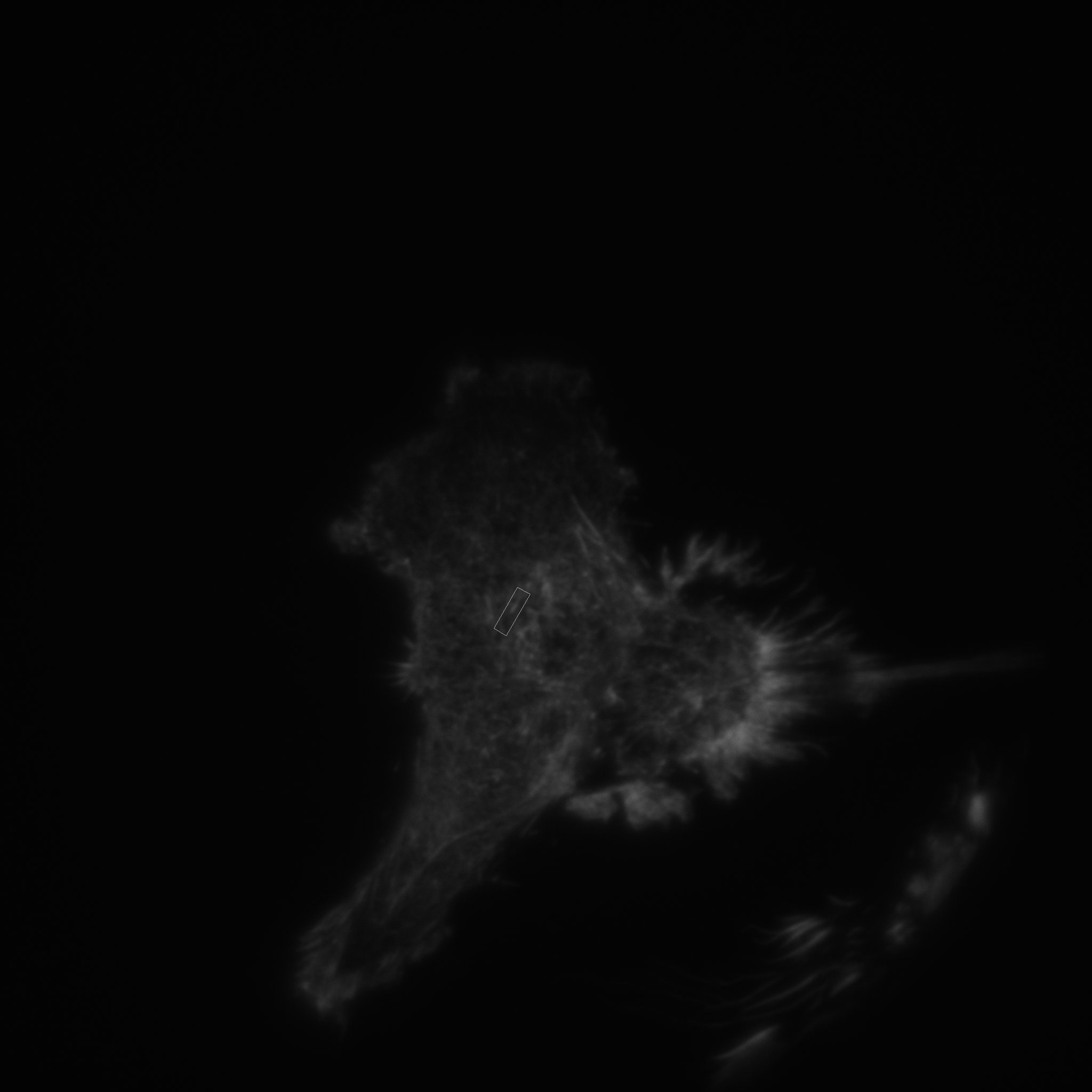

Supplement: Supplementary file 17 — Source data Fig. 2 [file 44319_2026_804_MOESM17_ESM.zip › Fig. 2/2D/SMIFH2/SMIFH2 untrimmed image.tif]

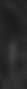

Supplement: Supplementary file 17 — Source data Fig. 2 [file 44319_2026_804_MOESM17_ESM.zip › Fig. 2/2D/SMIFH2/SMIFH2.tif]

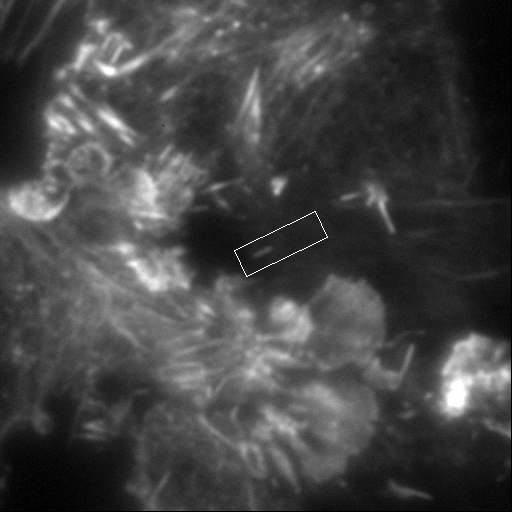

Supplement: Supplementary file 17 — Source data Fig. 2 [file 44319_2026_804_MOESM17_ESM.zip › Fig. 2/2D/VASP RNAi/VASP RNAi ROI.tif]

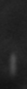

Supplement: Supplementary file 17 — Source data Fig. 2 [file 44319_2026_804_MOESM17_ESM.zip › Fig. 2/2D/VASP RNAi/VASP RNAi.tif]

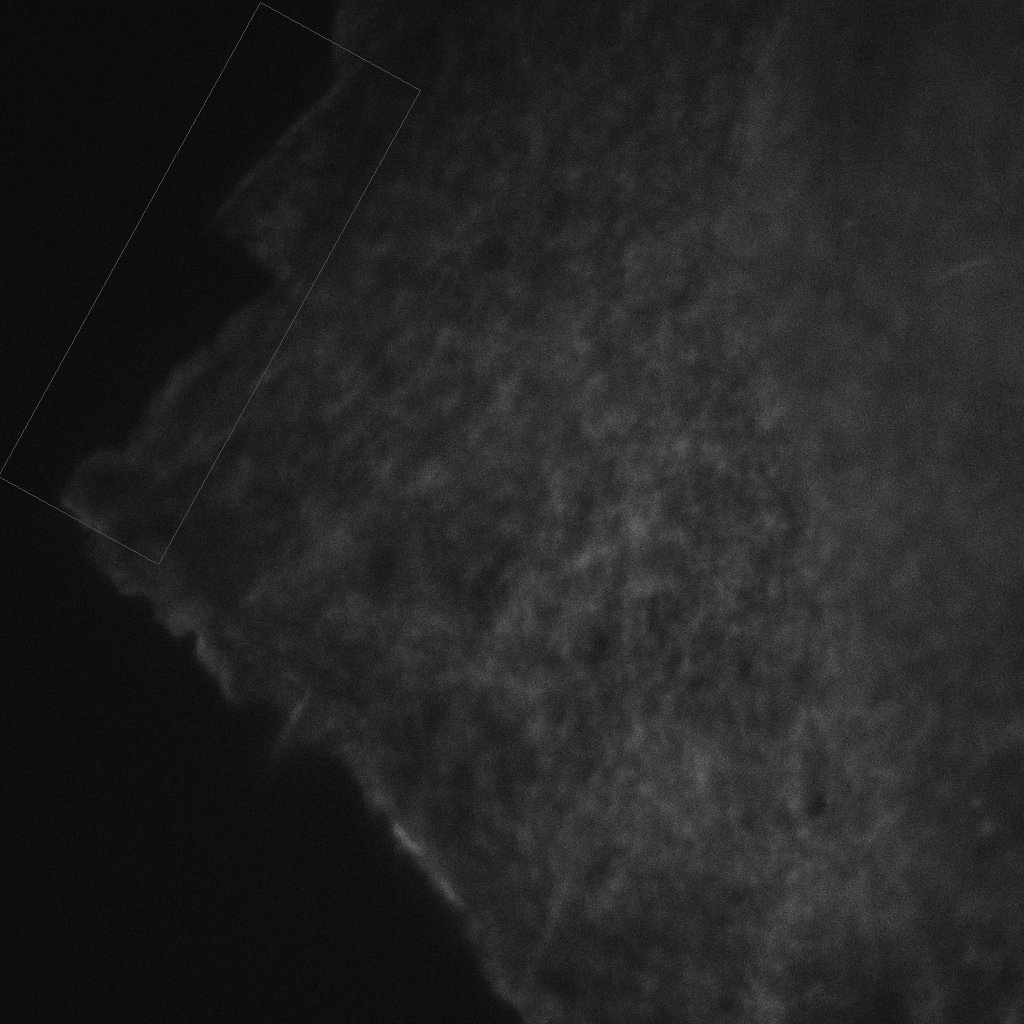

Supplement: Supplementary file 18 — Source data Fig. 3 [file 44319_2026_804_MOESM18_ESM.zip › Fig. 3/3E/3E.tif]

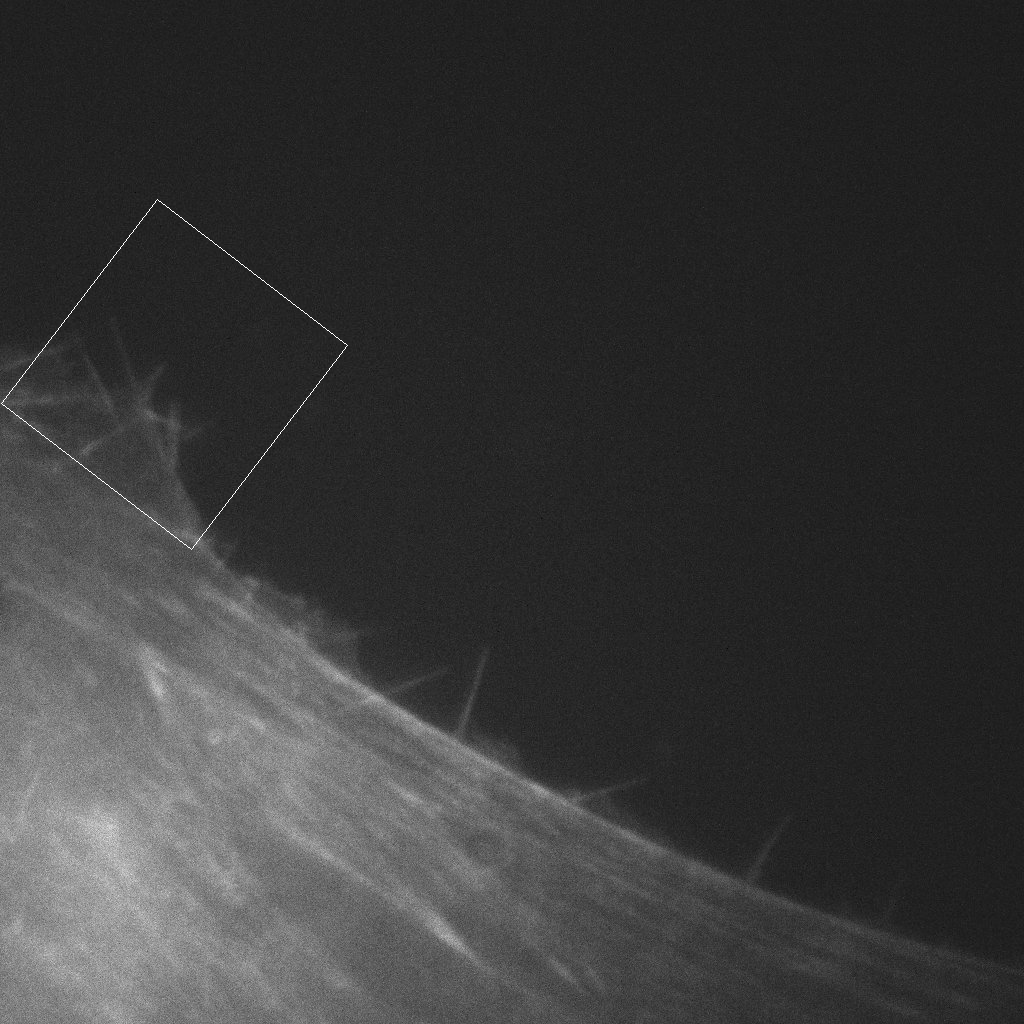

Supplement: Supplementary file 19 — Source data Fig. 4 [file 44319_2026_804_MOESM19_ESM.zip › Fig. 4/4A/4A.tif]

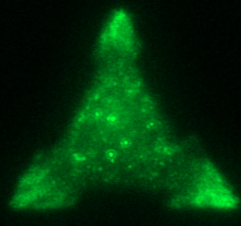

Supplement: Supplementary file 19 — Source data Fig. 4 [file 44319_2026_804_MOESM19_ESM.zip › Fig. 4/4C/ARPC2.tif]

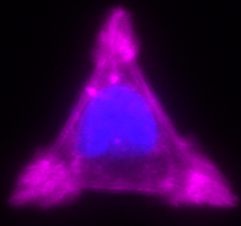

Supplement: Supplementary file 19 — Source data Fig. 4 [file 44319_2026_804_MOESM19_ESM.zip › Fig. 4/4C/F-actin DAPI.tif]

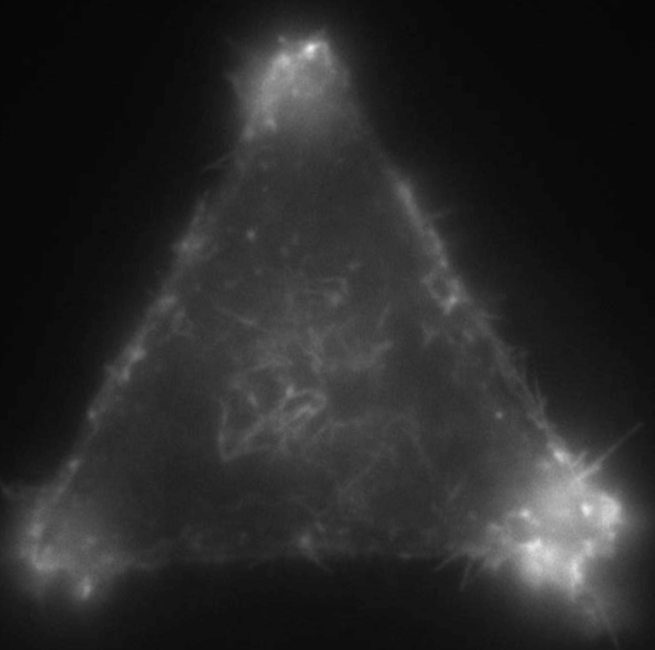

Supplement: Supplementary file 19 — Source data Fig. 4 [file 44319_2026_804_MOESM19_ESM.zip › Fig. 4/4D/4D.tif]

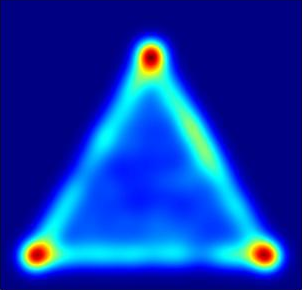

Supplement: Supplementary file 19 — Source data Fig. 4 [file 44319_2026_804_MOESM19_ESM.zip › Fig. 4/4E/4E.tif]

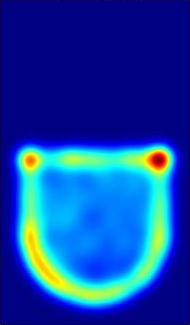

Supplement: Supplementary file 19 — Source data Fig. 4 [file 44319_2026_804_MOESM19_ESM.zip › Fig. 4/4F/4F.tif]

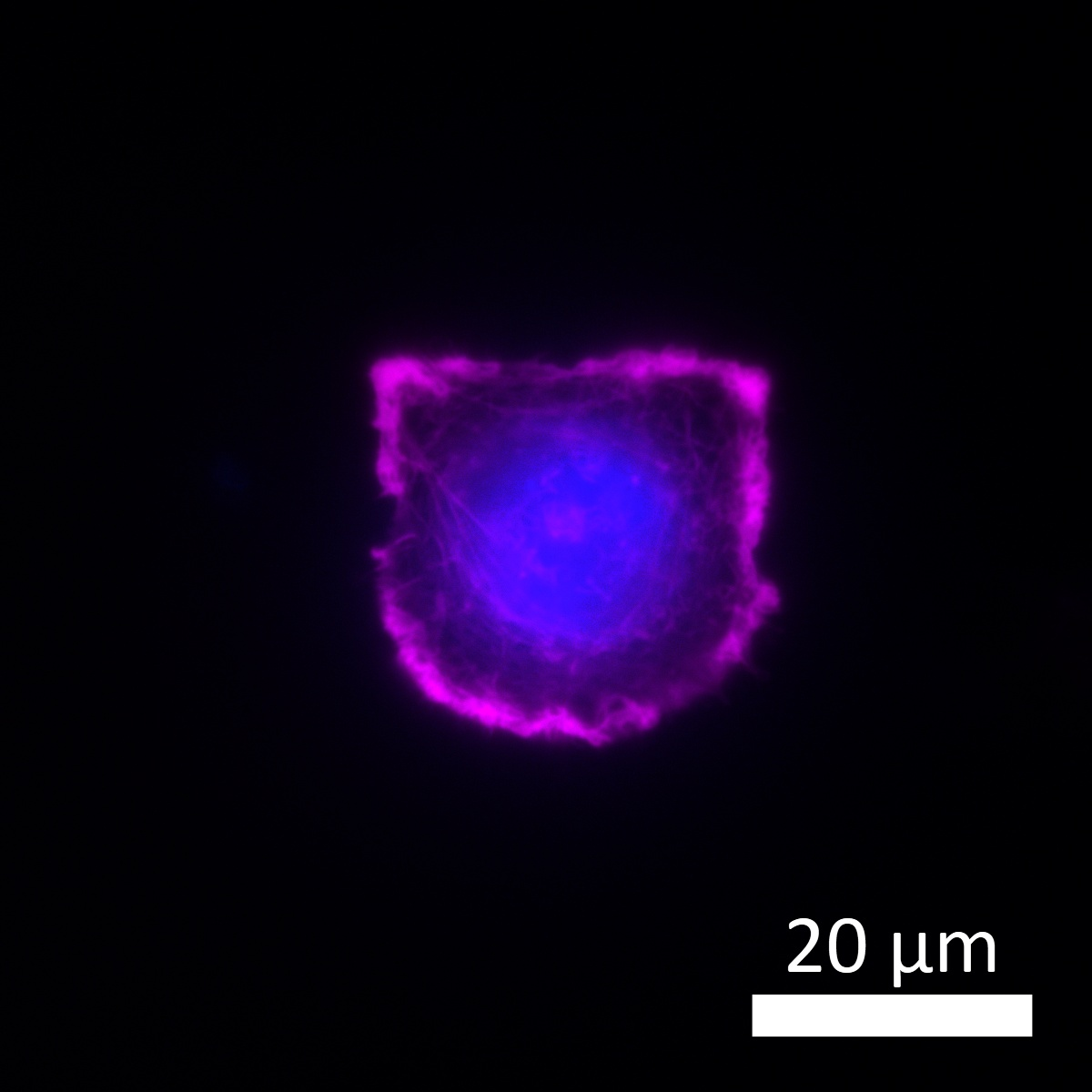

Supplement: Supplementary file 19 — Source data Fig. 4 [file 44319_2026_804_MOESM19_ESM.zip › Fig. 4/4G/4G.tif]

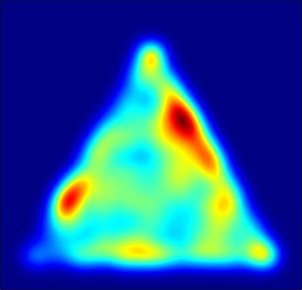

Supplement: Supplementary file 19 — Source data Fig. 4 [file 44319_2026_804_MOESM19_ESM.zip › Fig. 4/4H/4H.tif]

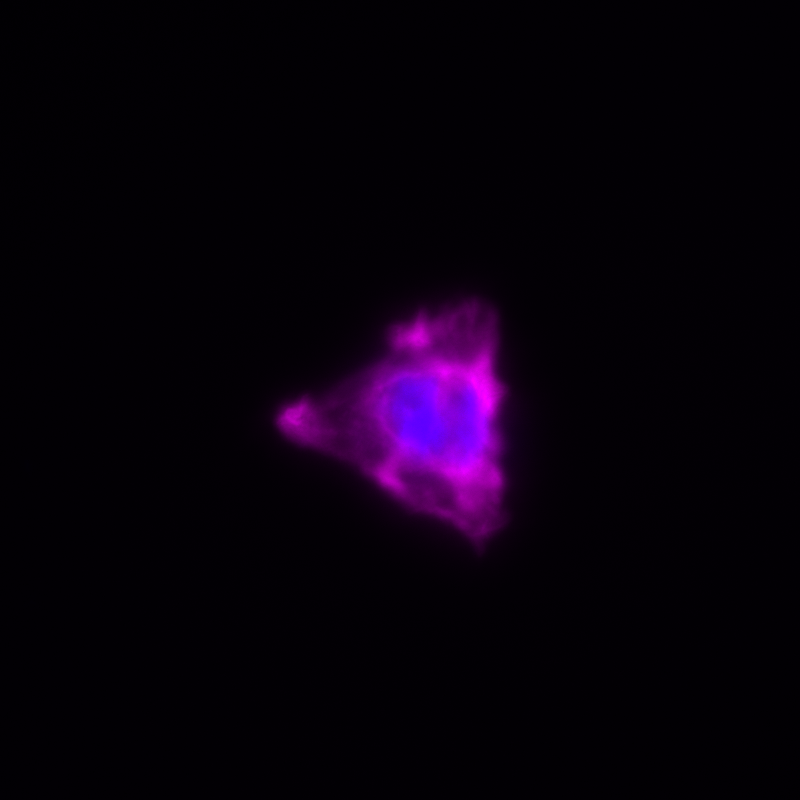

Supplement: Supplementary file 19 — Source data Fig. 4 [file 44319_2026_804_MOESM19_ESM.zip › Fig. 4/4I/F-actin DAPI.tif]

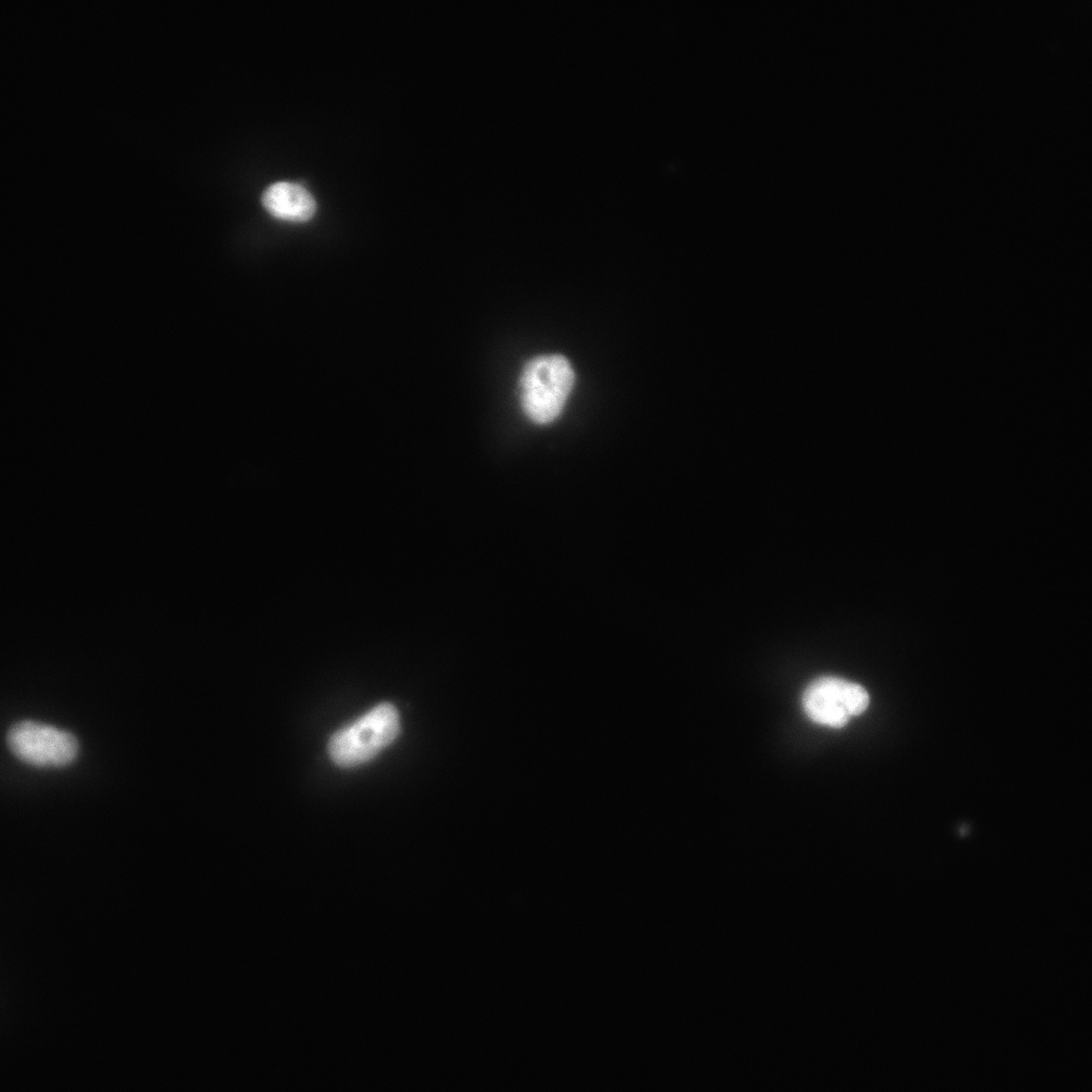

Supplement: Supplementary file 20 — Source data Fig. 5 [file 44319_2026_804_MOESM20_ESM.zip › Fig. 5/5A/KO1.tif]

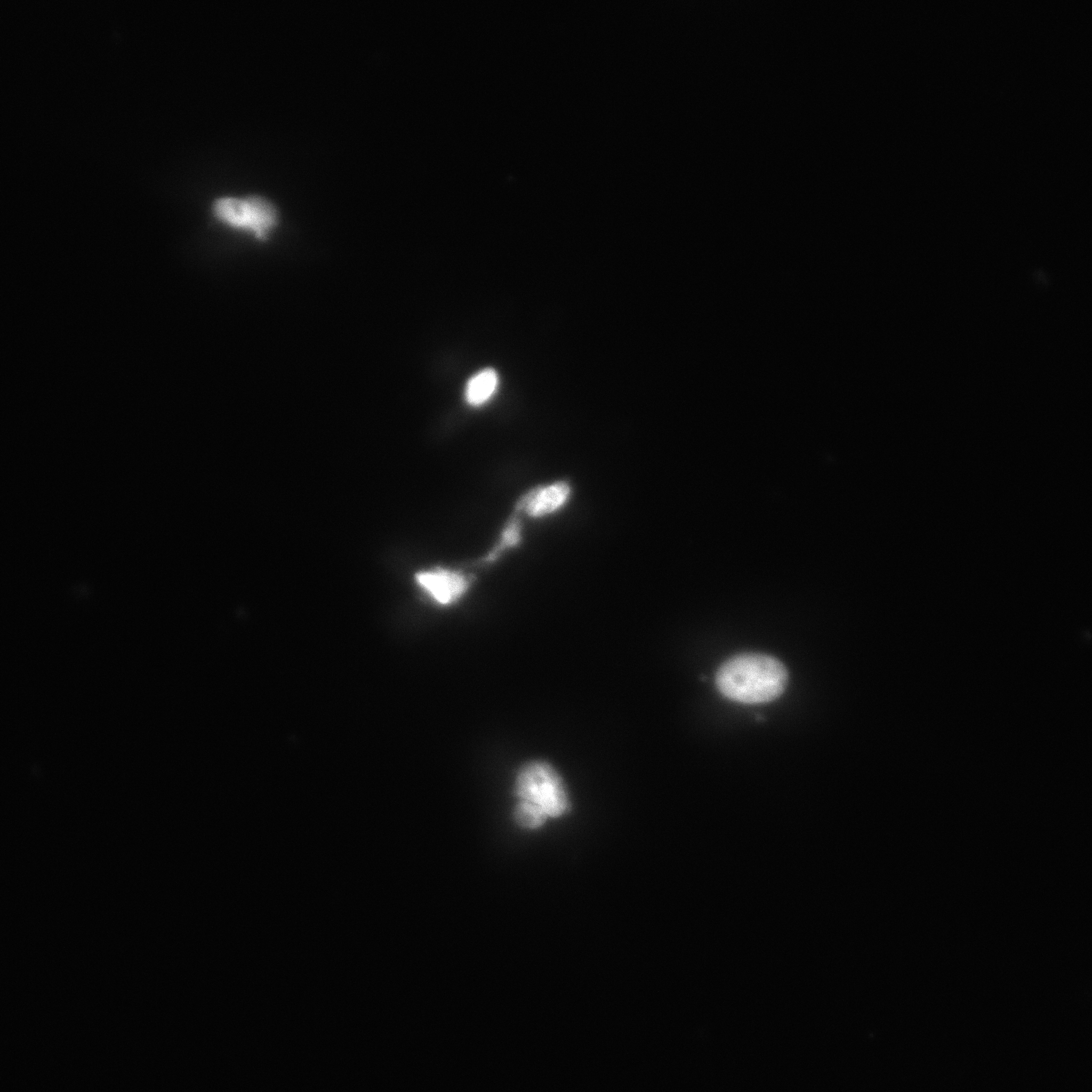

Supplement: Supplementary file 20 — Source data Fig. 5 [file 44319_2026_804_MOESM20_ESM.zip › Fig. 5/5A/KO2.tif]

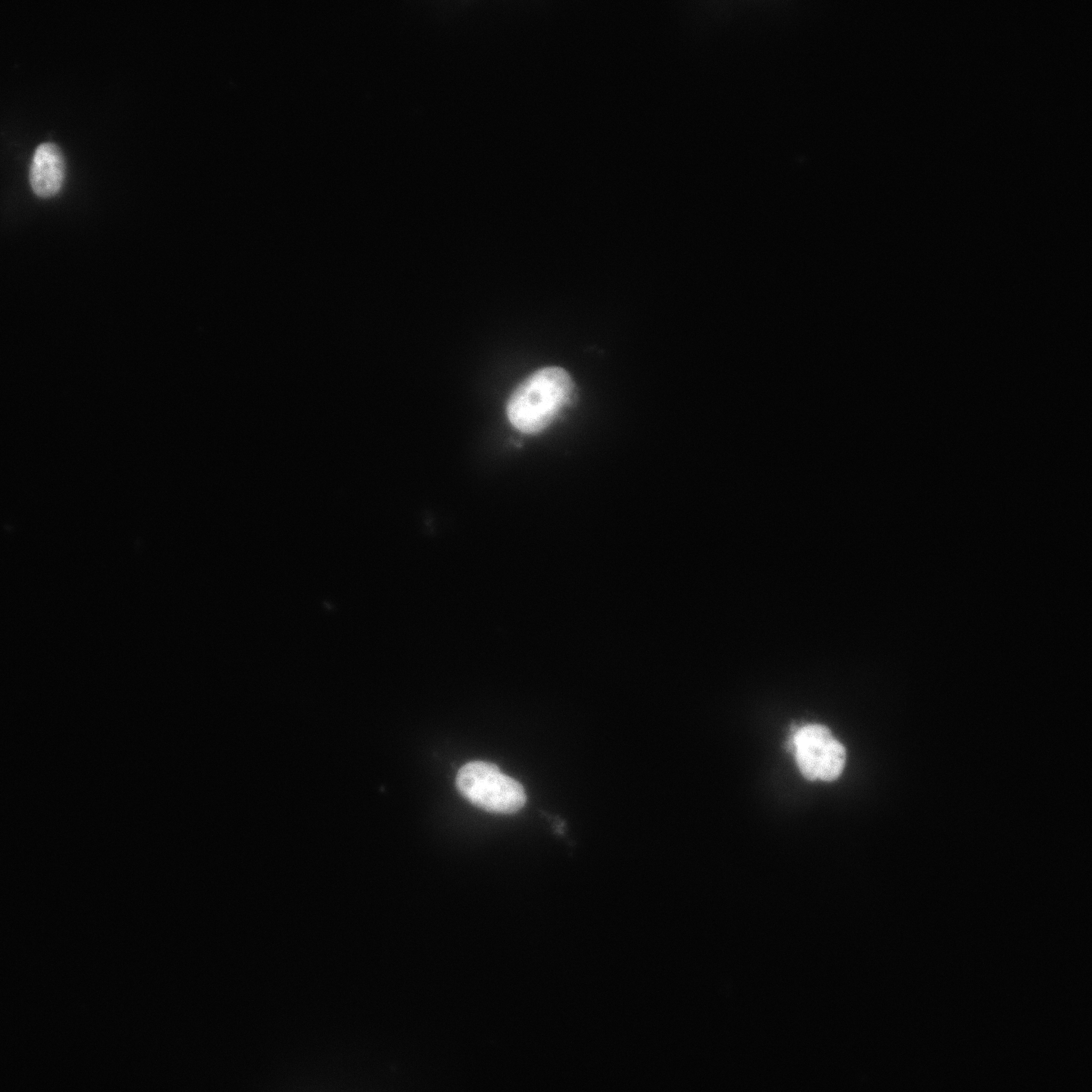

Supplement: Supplementary file 20 — Source data Fig. 5 [file 44319_2026_804_MOESM20_ESM.zip › Fig. 5/5A/WT.tif]

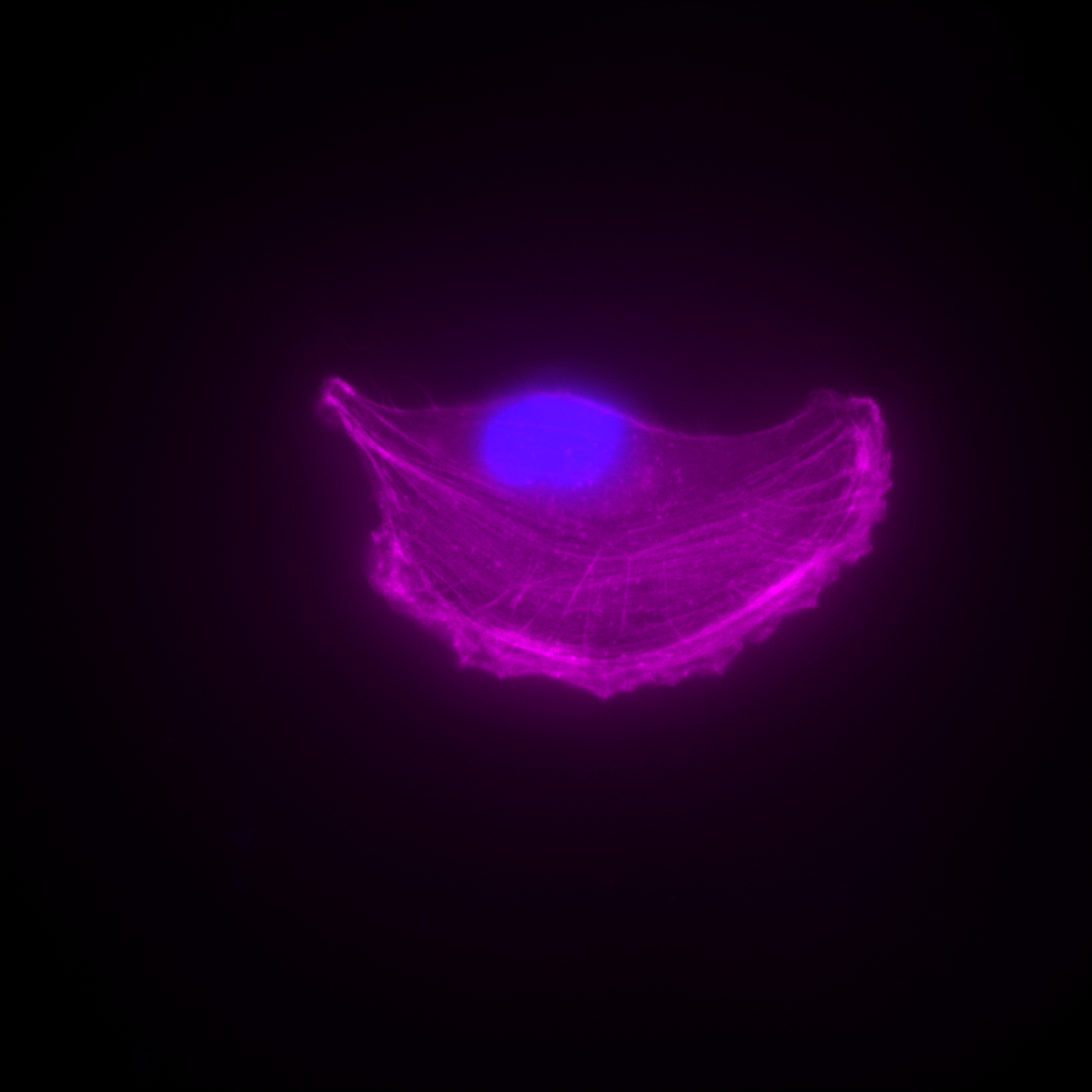

Supplement: Supplementary file 20 — Source data Fig. 5 [file 44319_2026_804_MOESM20_ESM.zip › Fig. 5/5C/F-actin DAPI.tif]

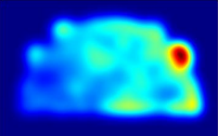

Supplement: Supplementary file 20 — Source data Fig. 5 [file 44319_2026_804_MOESM20_ESM.zip › Fig. 5/5E/KO.png]

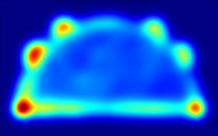

Supplement: Supplementary file 20 — Source data Fig. 5 [file 44319_2026_804_MOESM20_ESM.zip › Fig. 5/5E/WT.png]

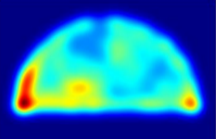

Supplement: Supplementary file 20 — Source data Fig. 5 [file 44319_2026_804_MOESM20_ESM.zip › Fig. 5/5F/KO.png]

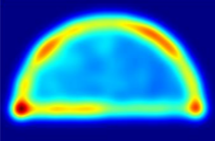

Supplement: Supplementary file 20 — Source data Fig. 5 [file 44319_2026_804_MOESM20_ESM.zip › Fig. 5/5F/WT.png]

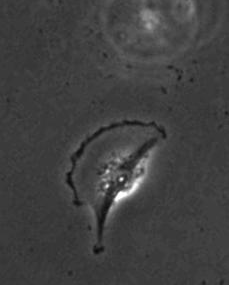

Supplement: Supplementary file 21 — Figure Source Data for Appendix Fig. S1 [file 44319_2026_804_MOESM21_ESM.zip › Appendix Fig. S1/S1A/S1A.tif]

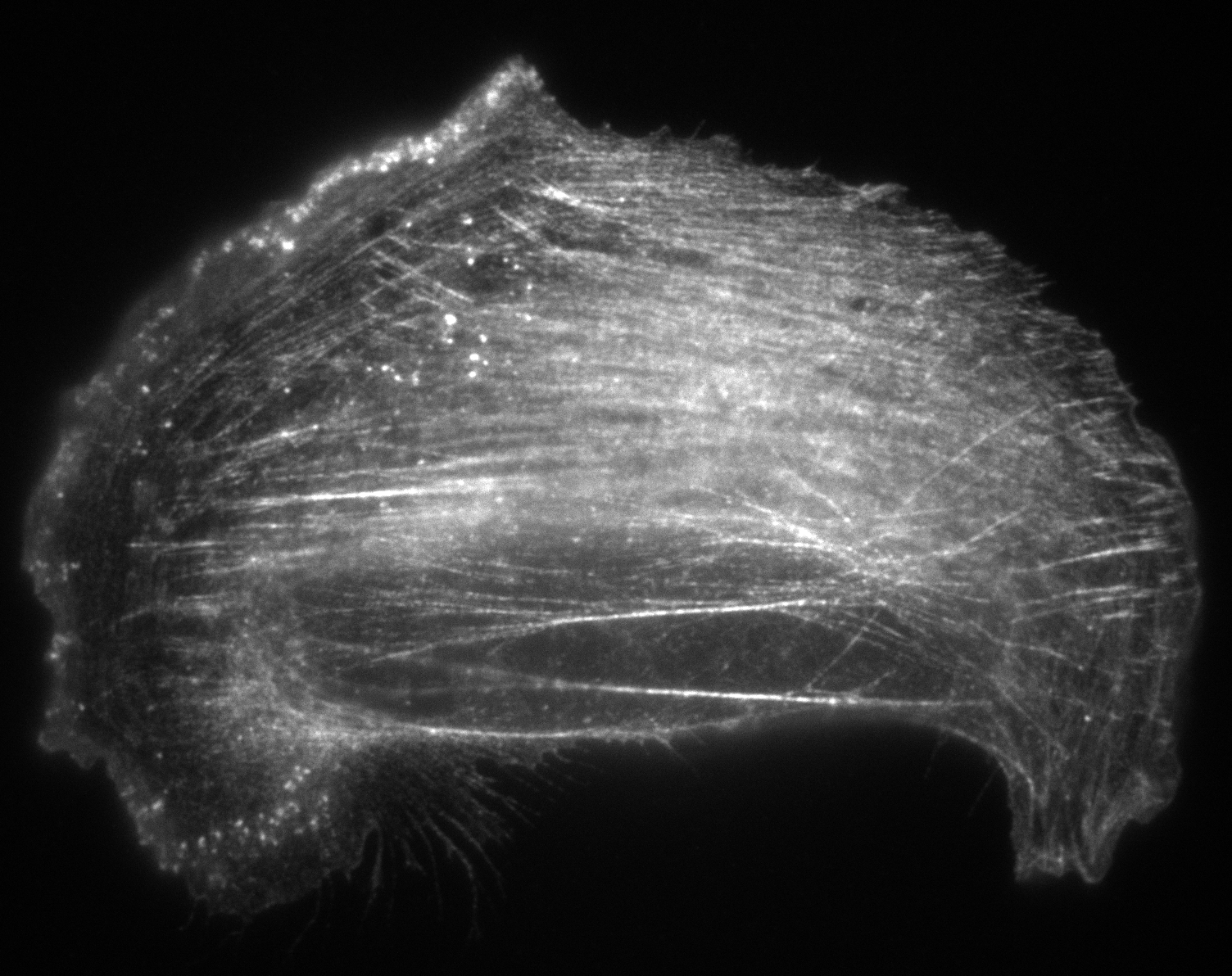

Supplement: Supplementary file 21 — Figure Source Data for Appendix Fig. S1 [file 44319_2026_804_MOESM21_ESM.zip › Appendix Fig. S1/S1B/S1B.tif]

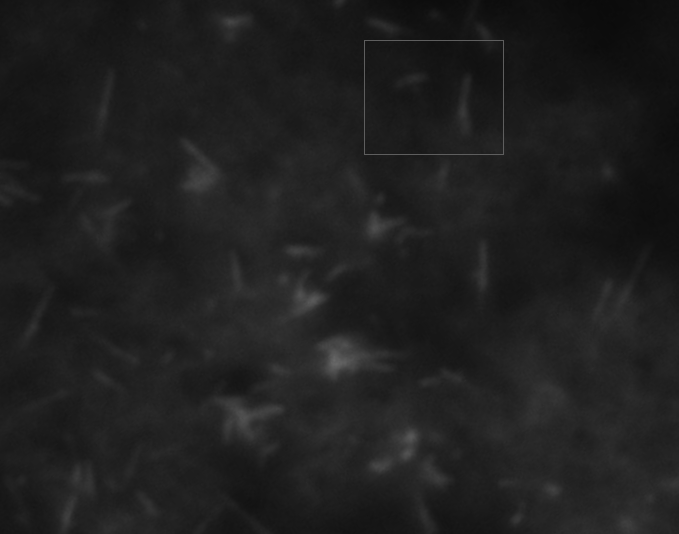

Supplement: Supplementary file 21 — Figure Source Data for Appendix Fig. S1 [file 44319_2026_804_MOESM21_ESM.zip › Appendix Fig. S1/S1C/Source Data for Appendix Fig S1C.tif]

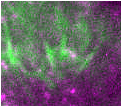

Supplement: Supplementary file 22 — Figure Source Data for Appendix Fig. S2 [file 44319_2026_804_MOESM22_ESM.zip › Appendix Fig. S2/S2B/Left.tif]

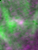

Supplement: Supplementary file 22 — Figure Source Data for Appendix Fig. S2 [file 44319_2026_804_MOESM22_ESM.zip › Appendix Fig. S2/S2B/Merge.tif]

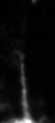

Supplement: Supplementary file 22 — Figure Source Data for Appendix Fig. S2 [file 44319_2026_804_MOESM22_ESM.zip › Appendix Fig. S2/S2C/1.tif]

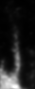

Supplement: Supplementary file 22 — Figure Source Data for Appendix Fig. S2 [file 44319_2026_804_MOESM22_ESM.zip › Appendix Fig. S2/S2C/2.tif]

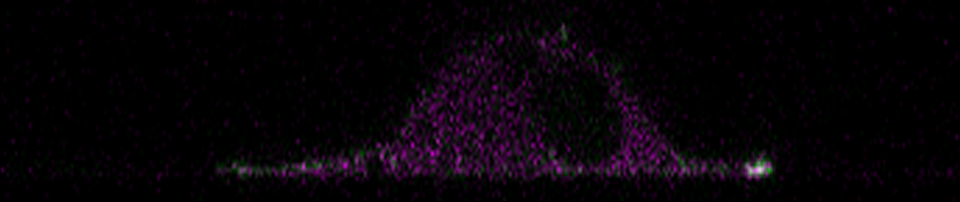

Supplement: Supplementary file 22 — Figure Source Data for Appendix Fig. S2 [file 44319_2026_804_MOESM22_ESM.zip › Appendix Fig. S2/S2C/Side view view.tif]

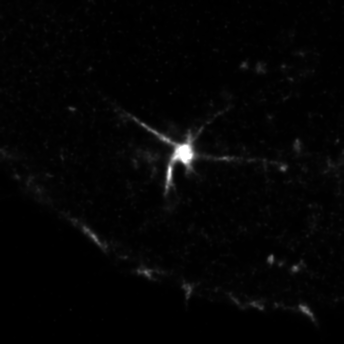

Supplement: Supplementary file 22 — Figure Source Data for Appendix Fig. S2 [file 44319_2026_804_MOESM22_ESM.zip › Appendix Fig. S2/S2C/Top view (right).tif]

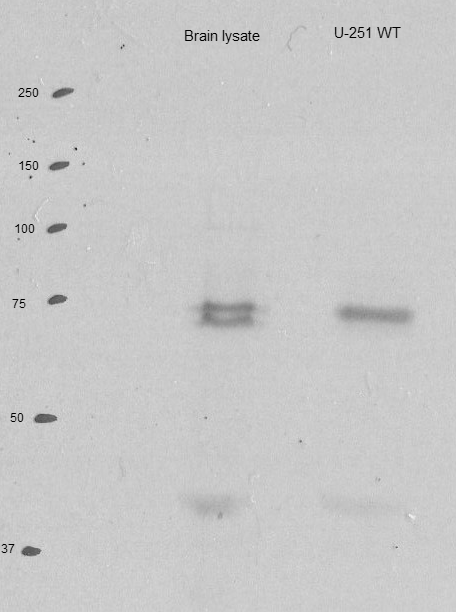

Supplement: Supplementary file 22 — Figure Source Data for Appendix Fig. S2 [file 44319_2026_804_MOESM22_ESM.zip › Appendix Fig. S2/S2D/Cortactin.tif]

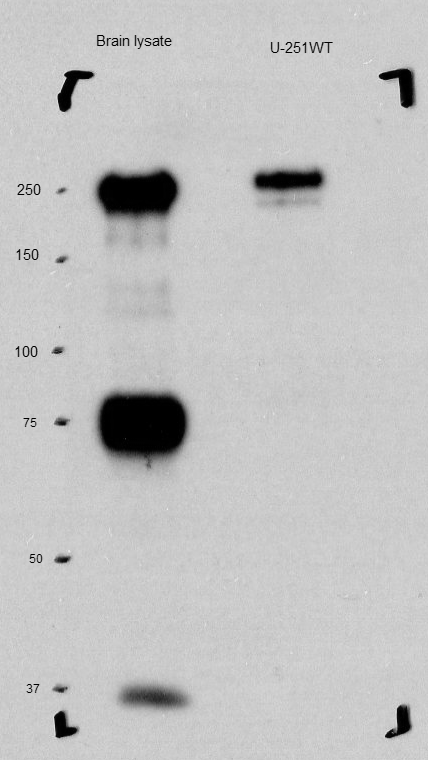

Supplement: Supplementary file 22 — Figure Source Data for Appendix Fig. S2 [file 44319_2026_804_MOESM22_ESM.zip › Appendix Fig. S2/S2D/L1.tif]

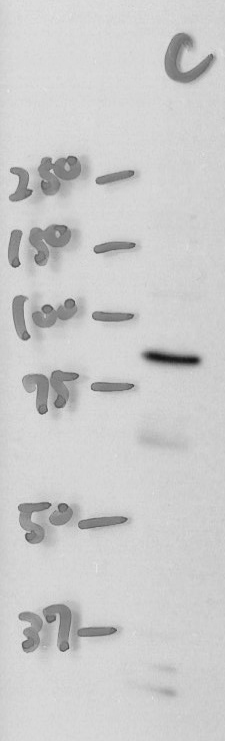

Supplement: Supplementary file 22 — Figure Source Data for Appendix Fig. S2 [file 44319_2026_804_MOESM22_ESM.zip › Appendix Fig. S2/S2D/Shootin1b.tif]

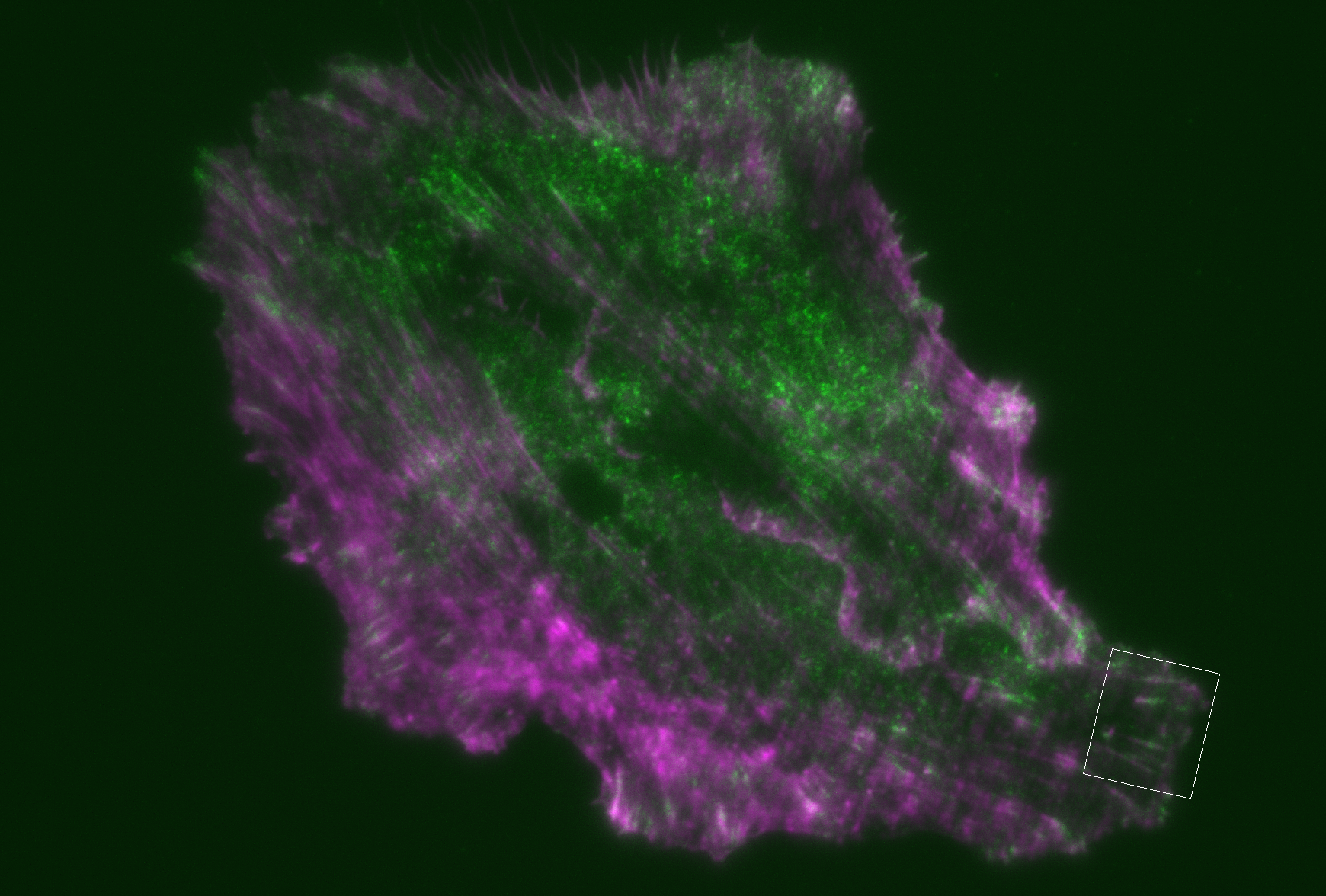

Supplement: Supplementary file 22 — Figure Source Data for Appendix Fig. S2 [file 44319_2026_804_MOESM22_ESM.zip › Appendix Fig. S2/S2E/Left untrimmed image.tif]

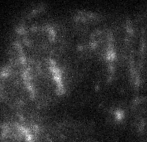

Supplement: Supplementary file 22 — Figure Source Data for Appendix Fig. S2 [file 44319_2026_804_MOESM22_ESM.zip › Appendix Fig. S2/S2E/Left.tif]

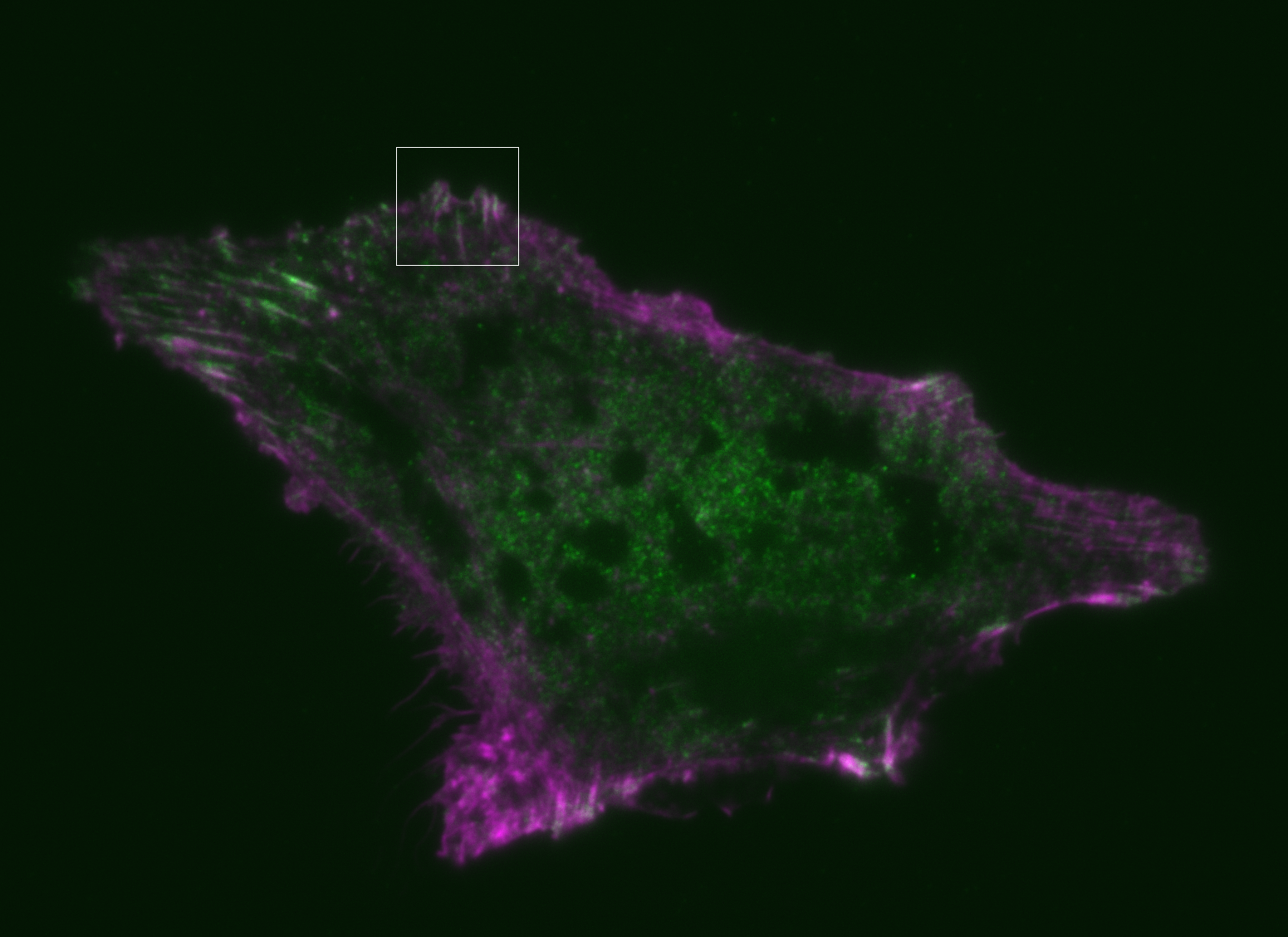

Supplement: Supplementary file 22 — Figure Source Data for Appendix Fig. S2 [file 44319_2026_804_MOESM22_ESM.zip › Appendix Fig. S2/S2E/Right untrimmed image.tif]

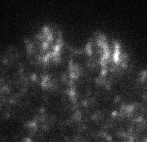

Supplement: Supplementary file 22 — Figure Source Data for Appendix Fig. S2 [file 44319_2026_804_MOESM22_ESM.zip › Appendix Fig. S2/S2E/Right.tif]

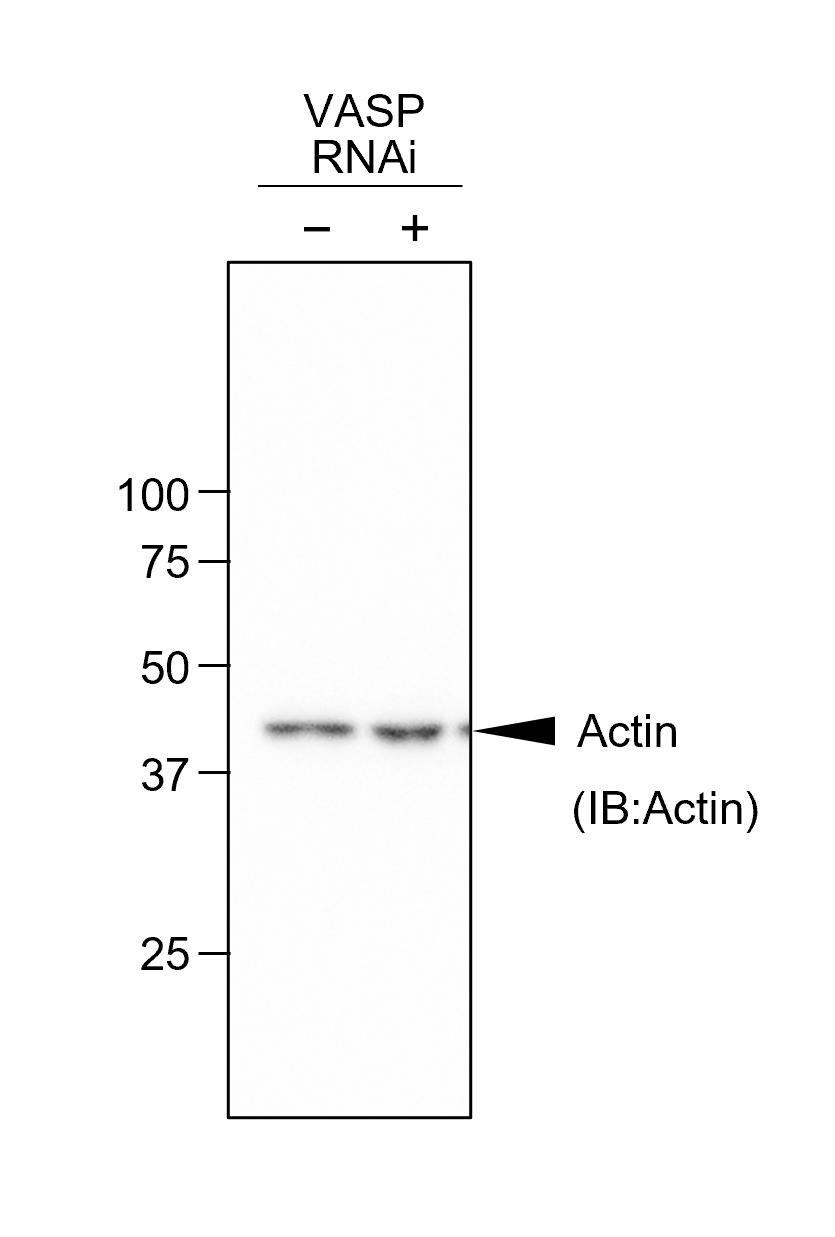

Supplement: Supplementary file 23 — Figure Source Data for Appendix Fig. S3 [file 44319_2026_804_MOESM23_ESM.zip › Appendix Fig. S3/S3A/Actin.png]

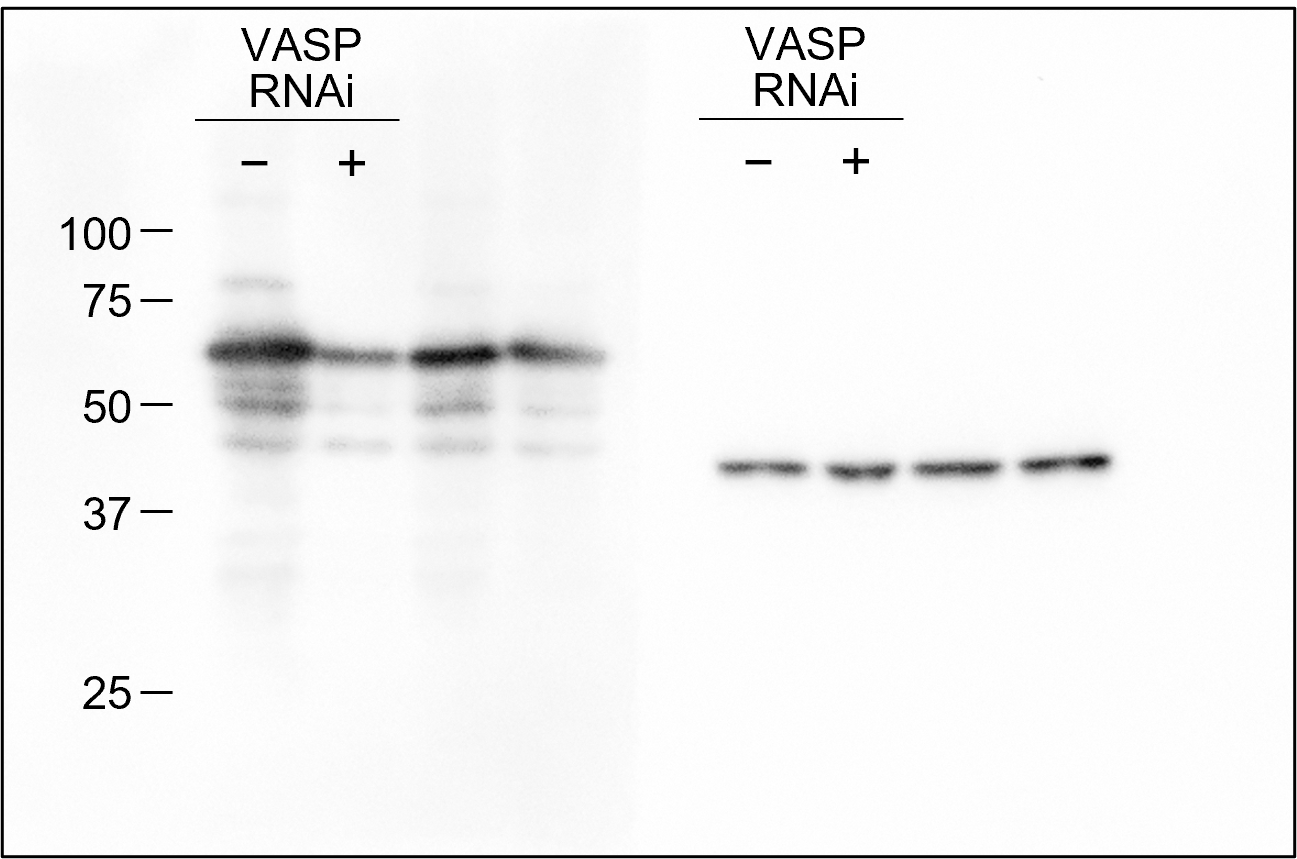

Supplement: Supplementary file 23 — Figure Source Data for Appendix Fig. S3 [file 44319_2026_804_MOESM23_ESM.zip › Appendix Fig. S3/S3A/Untrimmed image.png]

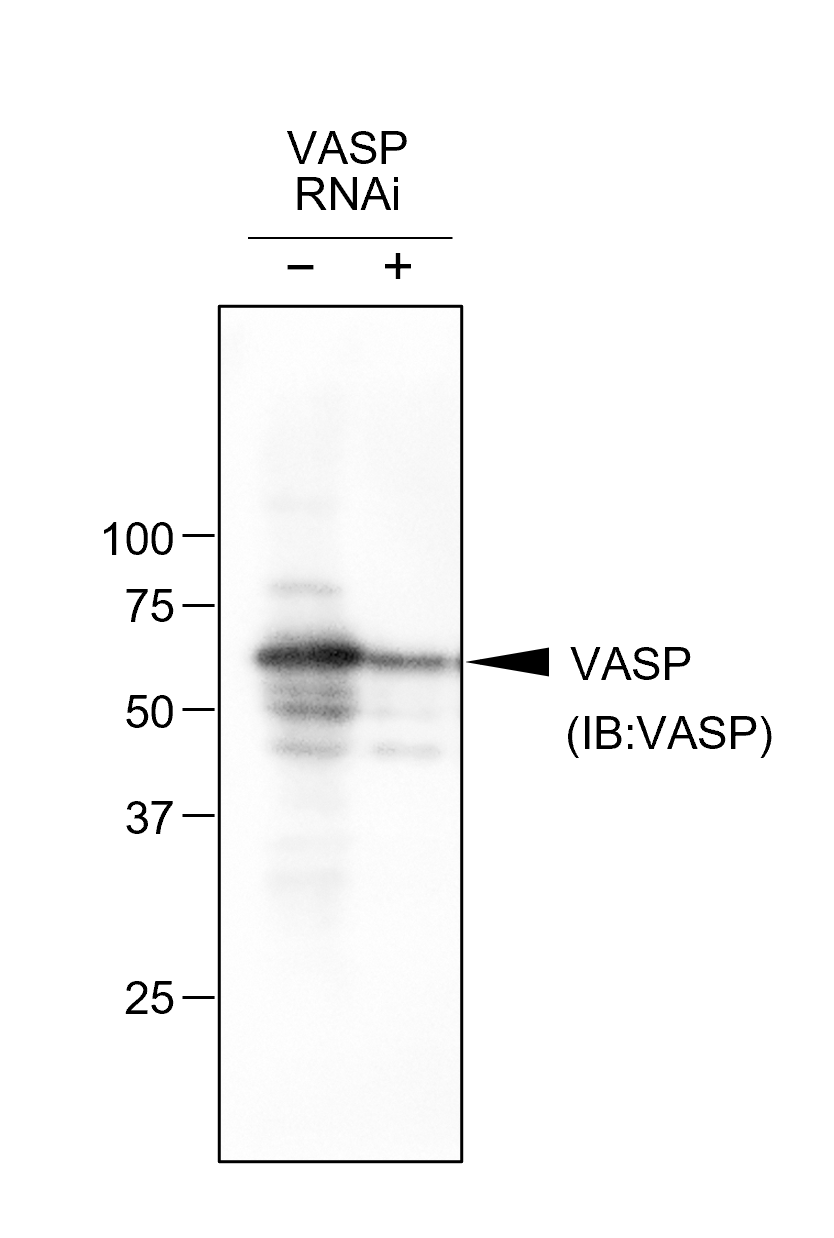

Supplement: Supplementary file 23 — Figure Source Data for Appendix Fig. S3 [file 44319_2026_804_MOESM23_ESM.zip › Appendix Fig. S3/S3A/VASP.png]

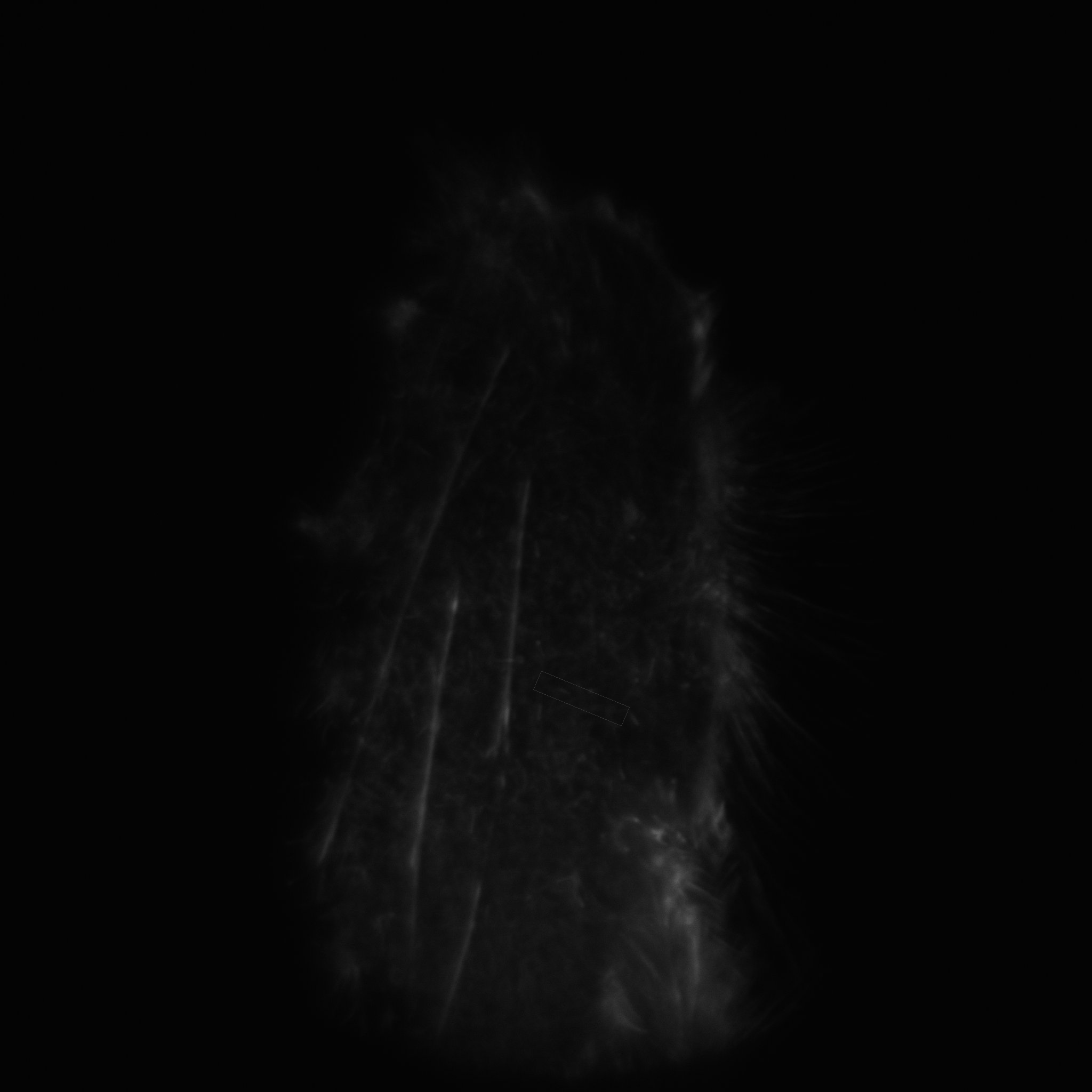

Supplement: Supplementary file 23 — Figure Source Data for Appendix Fig. S3 [file 44319_2026_804_MOESM23_ESM.zip › Appendix Fig. S3/S3B/CK666 untrimmed image.tif]

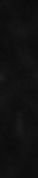

Supplement: Supplementary file 23 — Figure Source Data for Appendix Fig. S3 [file 44319_2026_804_MOESM23_ESM.zip › Appendix Fig. S3/S3B/CK666.tif]

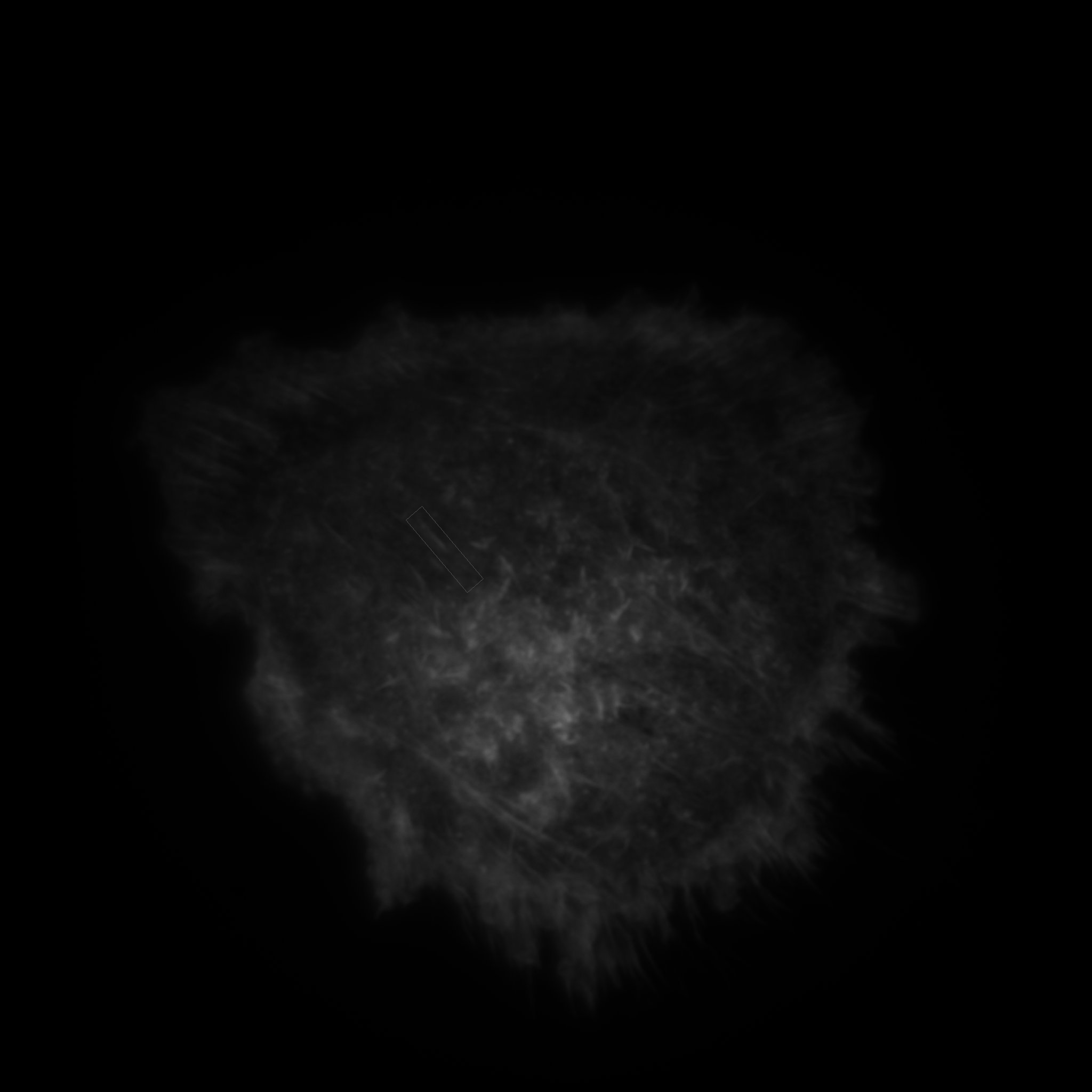

Supplement: Supplementary file 23 — Figure Source Data for Appendix Fig. S3 [file 44319_2026_804_MOESM23_ESM.zip › Appendix Fig. S3/S3B/DMSO untrimmed image.tif]

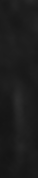

Supplement: Supplementary file 23 — Figure Source Data for Appendix Fig. S3 [file 44319_2026_804_MOESM23_ESM.zip › Appendix Fig. S3/S3B/DMSO.tif]

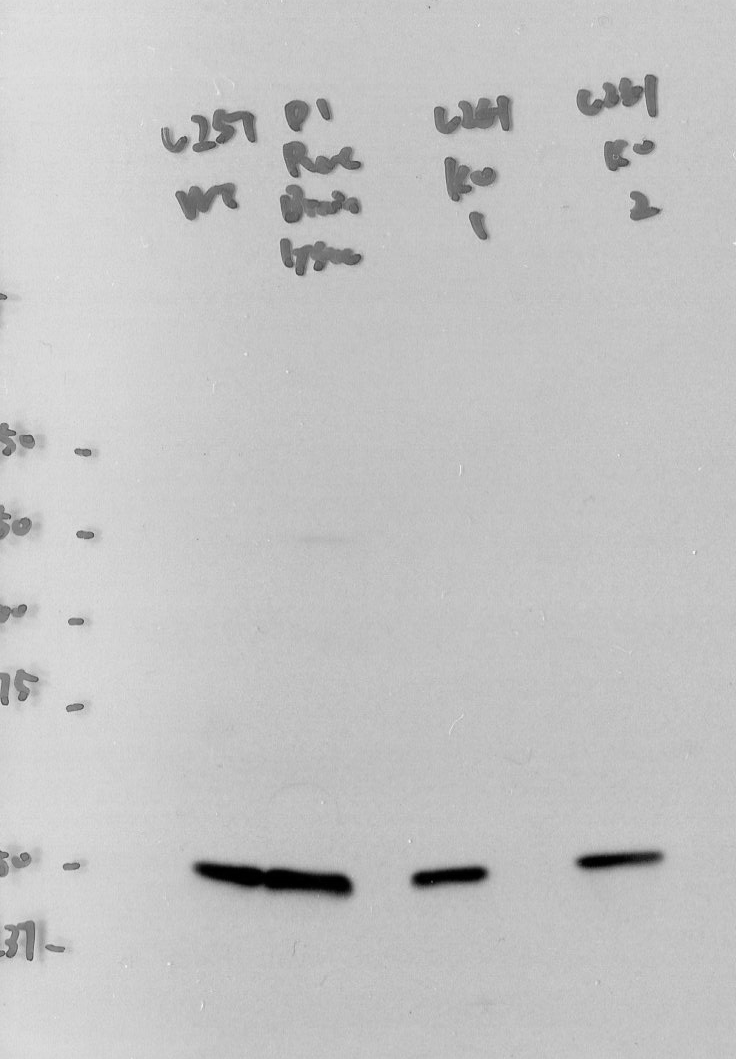

Supplement: Supplementary file 23 — Figure Source Data for Appendix Fig. S3 [file 44319_2026_804_MOESM23_ESM.zip › Appendix Fig. S3/S3D/Actin.tif]

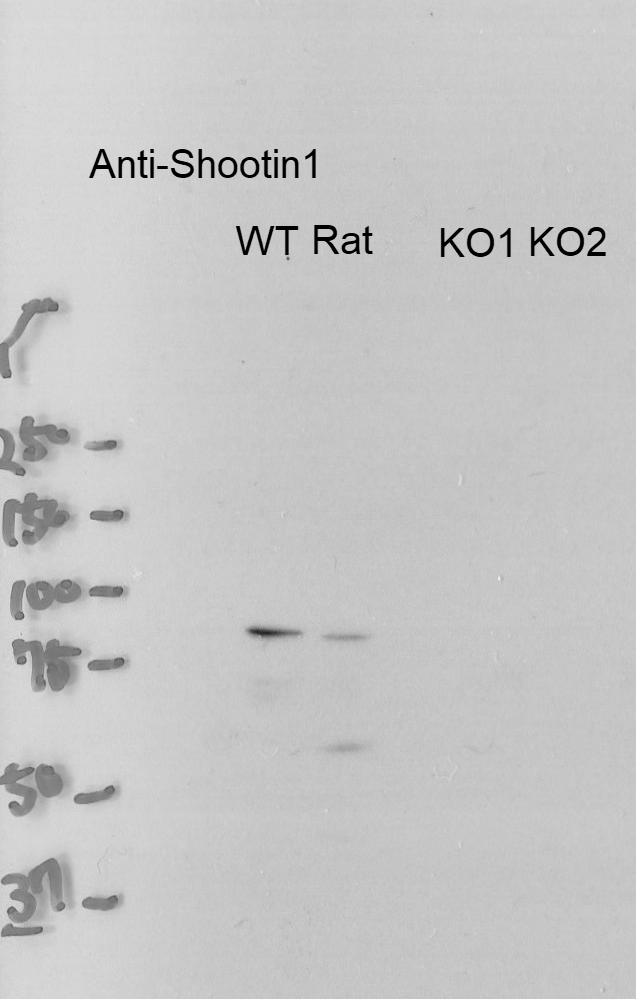

Supplement: Supplementary file 23 — Figure Source Data for Appendix Fig. S3 [file 44319_2026_804_MOESM23_ESM.zip › Appendix Fig. S3/S3D/Shootin1b.tif]

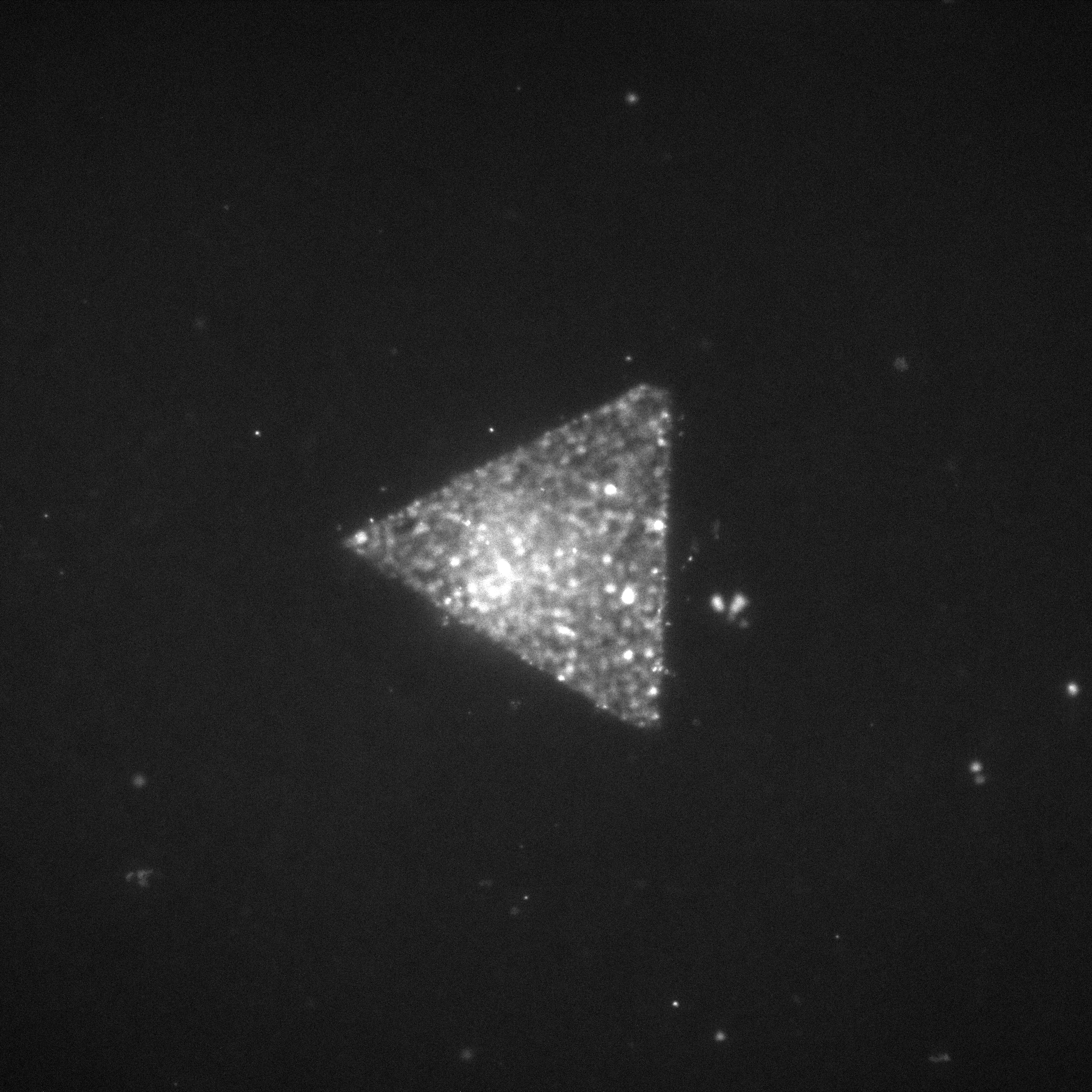

Supplement: Supplementary file 24 — Figure Source Data for Appendix Fig. S4 [file 44319_2026_804_MOESM24_ESM.zip › Appendix Fig. S4/Laminin.tif]

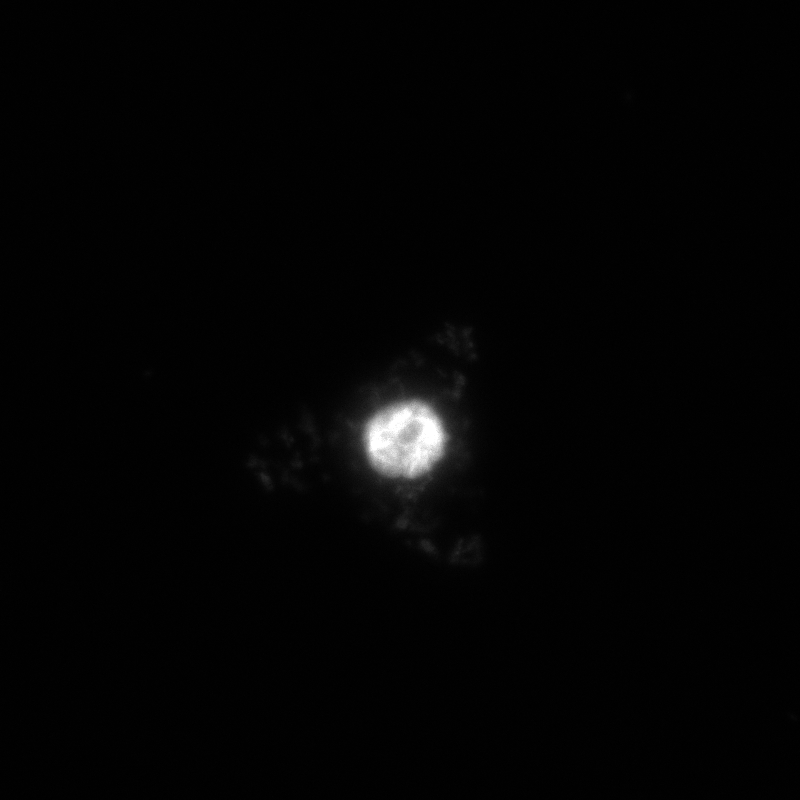

Supplement: Supplementary file 25 — Figure Source Data for Appendix Fig. S5 [file 44319_2026_804_MOESM25_ESM.zip › Appendix Fig. S5/S5G.tif]

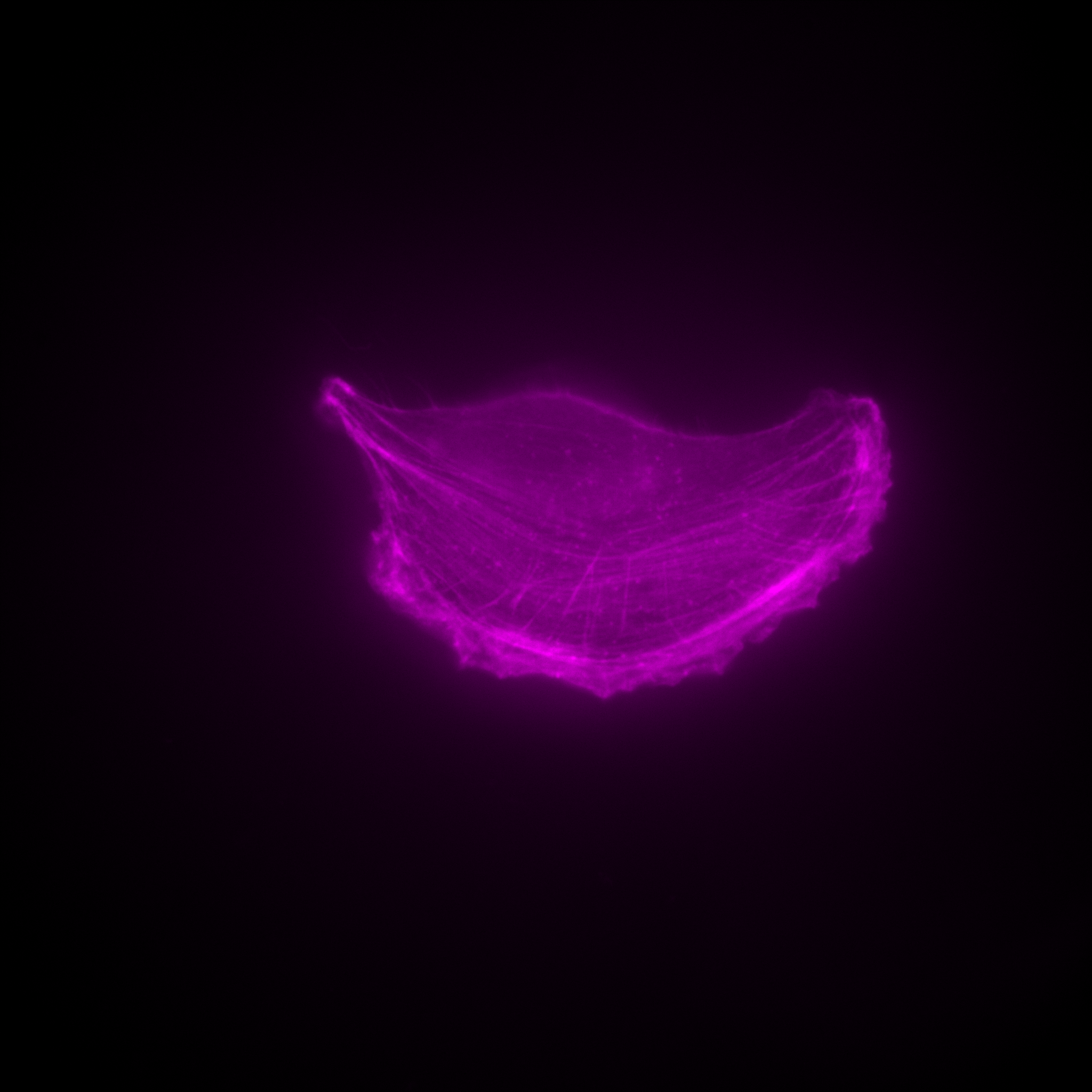

Supplement: Supplementary file 26 — Figure Source Data for Appendix Fig. S6 [file 44319_2026_804_MOESM26_ESM.zip › Appendix Fig. S6/S6A/F-actin.tif]

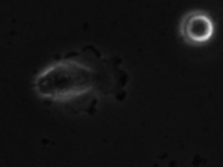

Supplement: Supplementary file 26 — Figure Source Data for Appendix Fig. S6 [file 44319_2026_804_MOESM26_ESM.zip › Appendix Fig. S6/S6D/KO.tif]

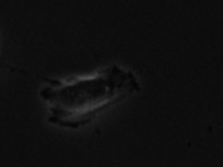

Supplement: Supplementary file 26 — Figure Source Data for Appendix Fig. S6 [file 44319_2026_804_MOESM26_ESM.zip › Appendix Fig. S6/S6D/WT.tif]

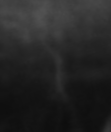

Supplement: Supplementary file 27 — Figure Source Data for Appendix Fig. S7 [file 44319_2026_804_MOESM27_ESM.zip › Appendix Fig. S7/S7A/Lower.tif]

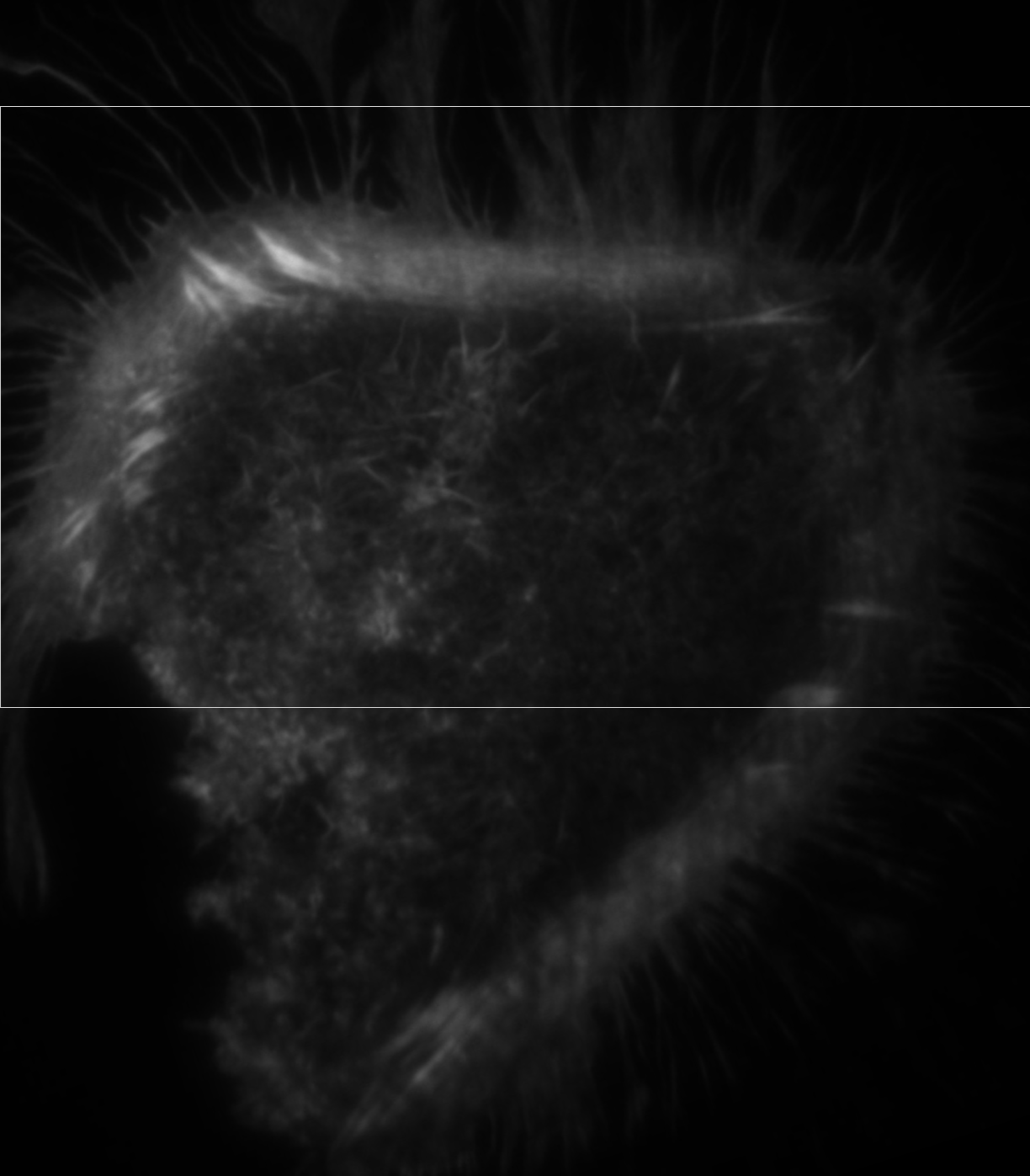

Supplement: Supplementary file 27 — Figure Source Data for Appendix Fig. S7 [file 44319_2026_804_MOESM27_ESM.zip › Appendix Fig. S7/S7A/Upper.tif]

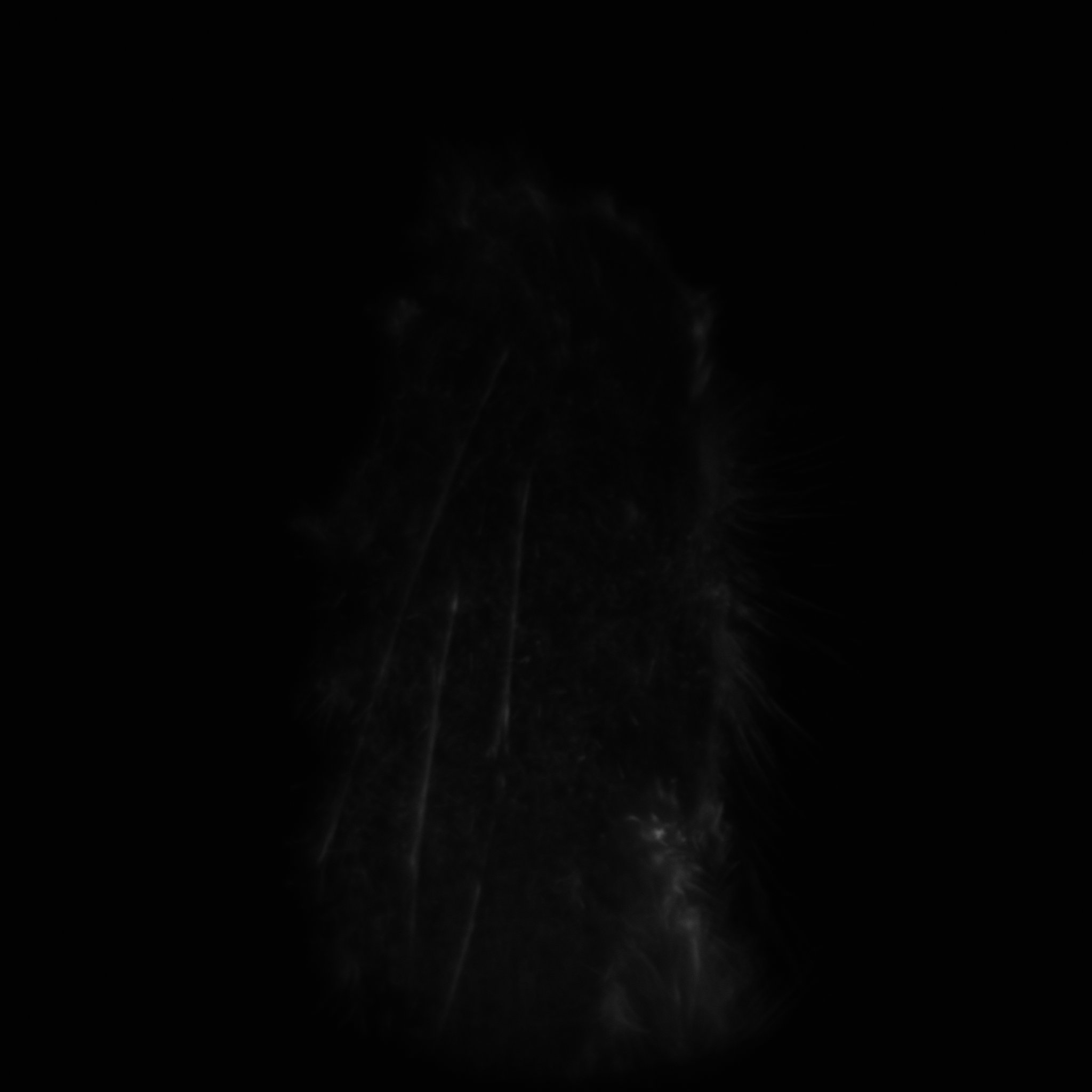

Supplement: Supplementary file 27 — Figure Source Data for Appendix Fig. S7 [file 44319_2026_804_MOESM27_ESM.zip › Appendix Fig. S7/S7D/CK666 after.tif]

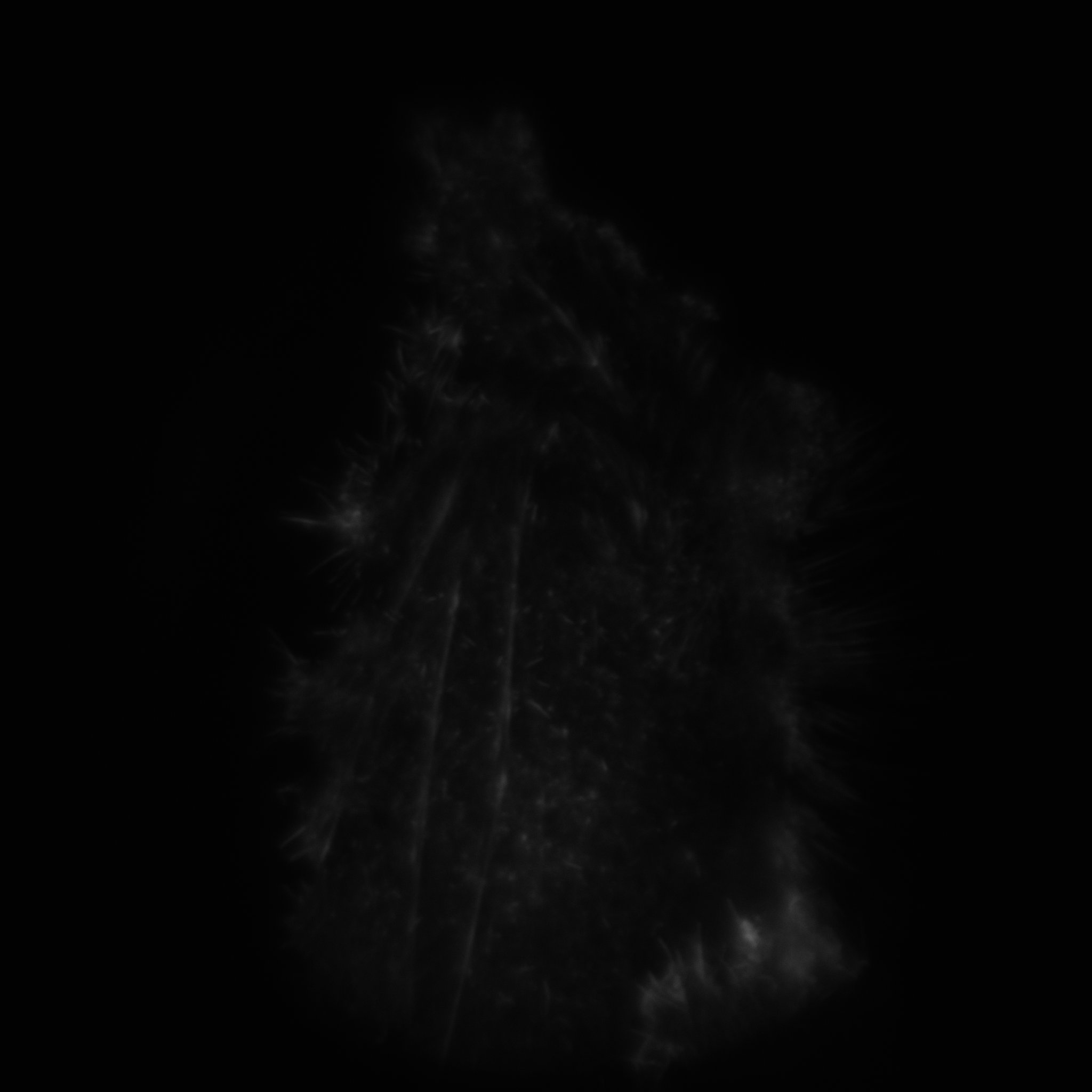

Supplement: Supplementary file 27 — Figure Source Data for Appendix Fig. S7 [file 44319_2026_804_MOESM27_ESM.zip › Appendix Fig. S7/S7D/CK666 before.tif]

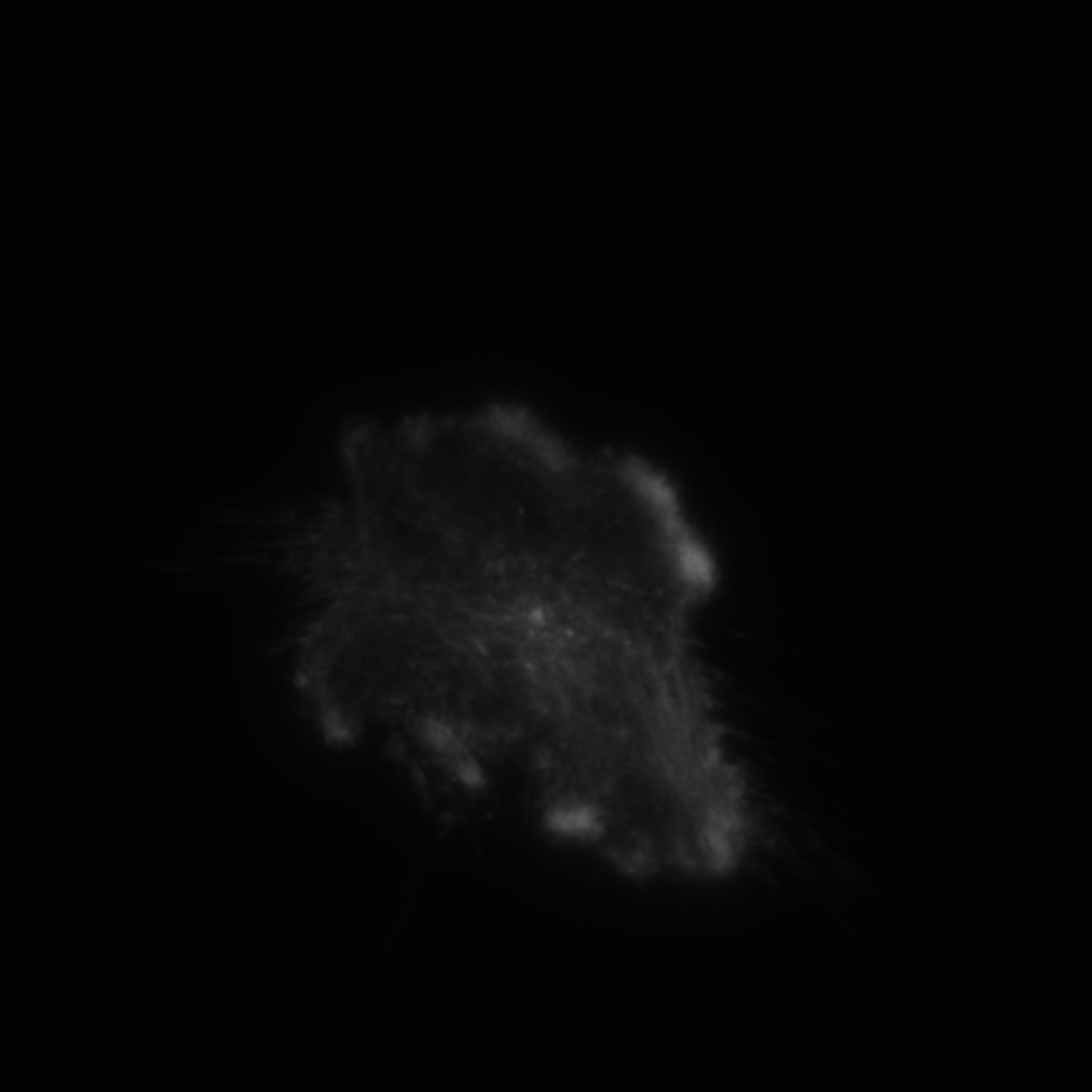

Supplement: Supplementary file 27 — Figure Source Data for Appendix Fig. S7 [file 44319_2026_804_MOESM27_ESM.zip › Appendix Fig. S7/S7D/Cytochalasin B after.tif]

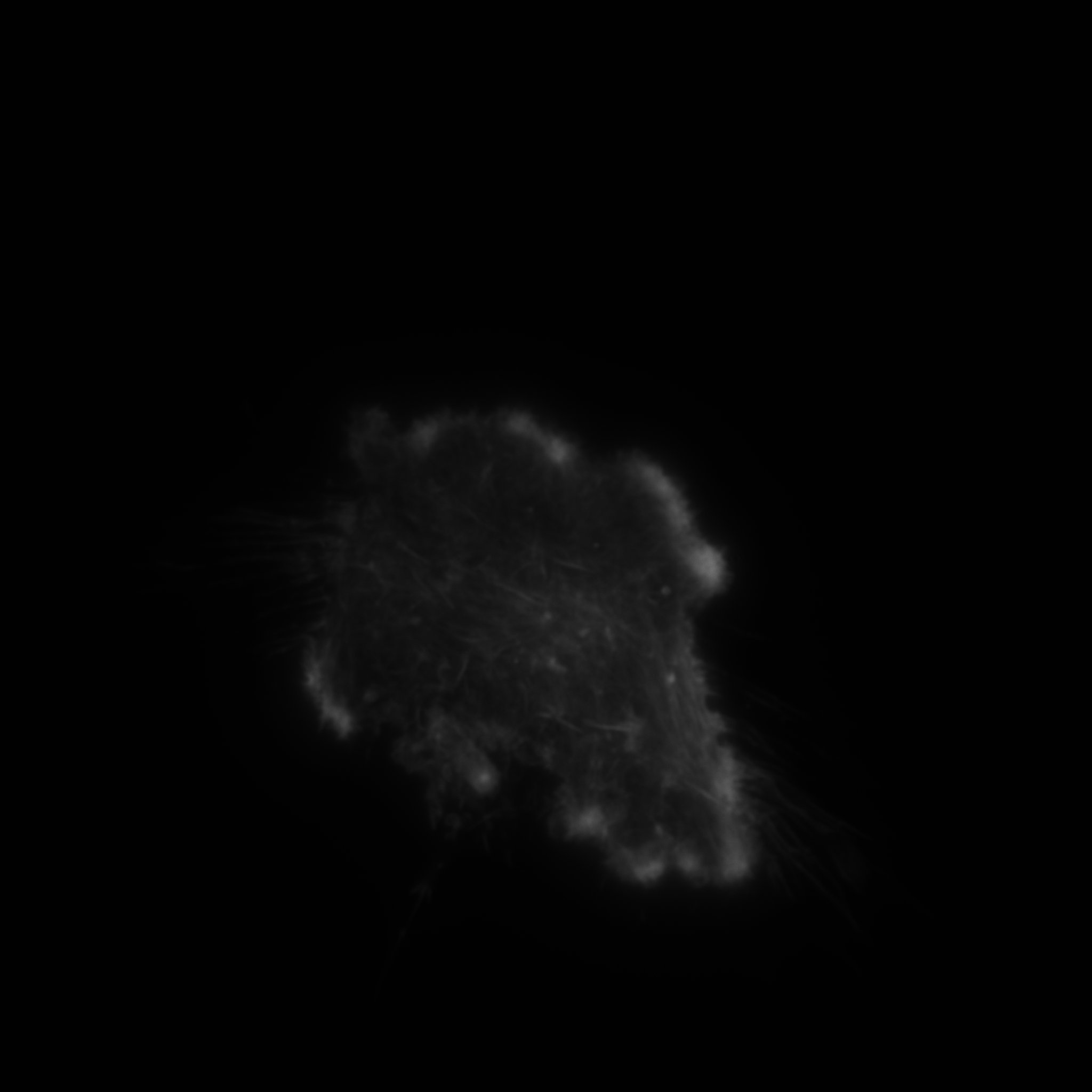

Supplement: Supplementary file 27 — Figure Source Data for Appendix Fig. S7 [file 44319_2026_804_MOESM27_ESM.zip › Appendix Fig. S7/S7D/Cytochalasin B before.tif]

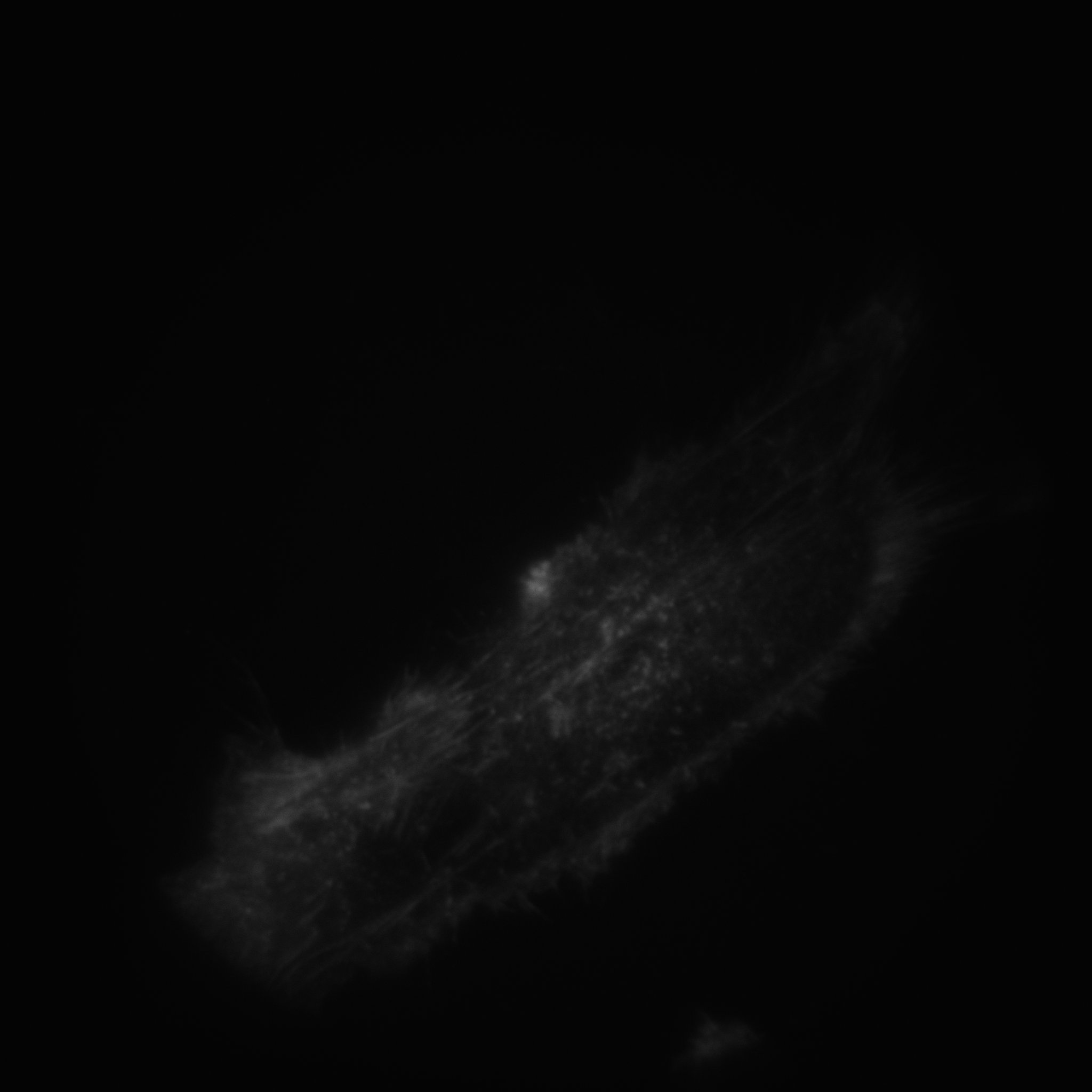

Supplement: Supplementary file 27 — Figure Source Data for Appendix Fig. S7 [file 44319_2026_804_MOESM27_ESM.zip › Appendix Fig. S7/S7D/SMIFH2 after.tif]

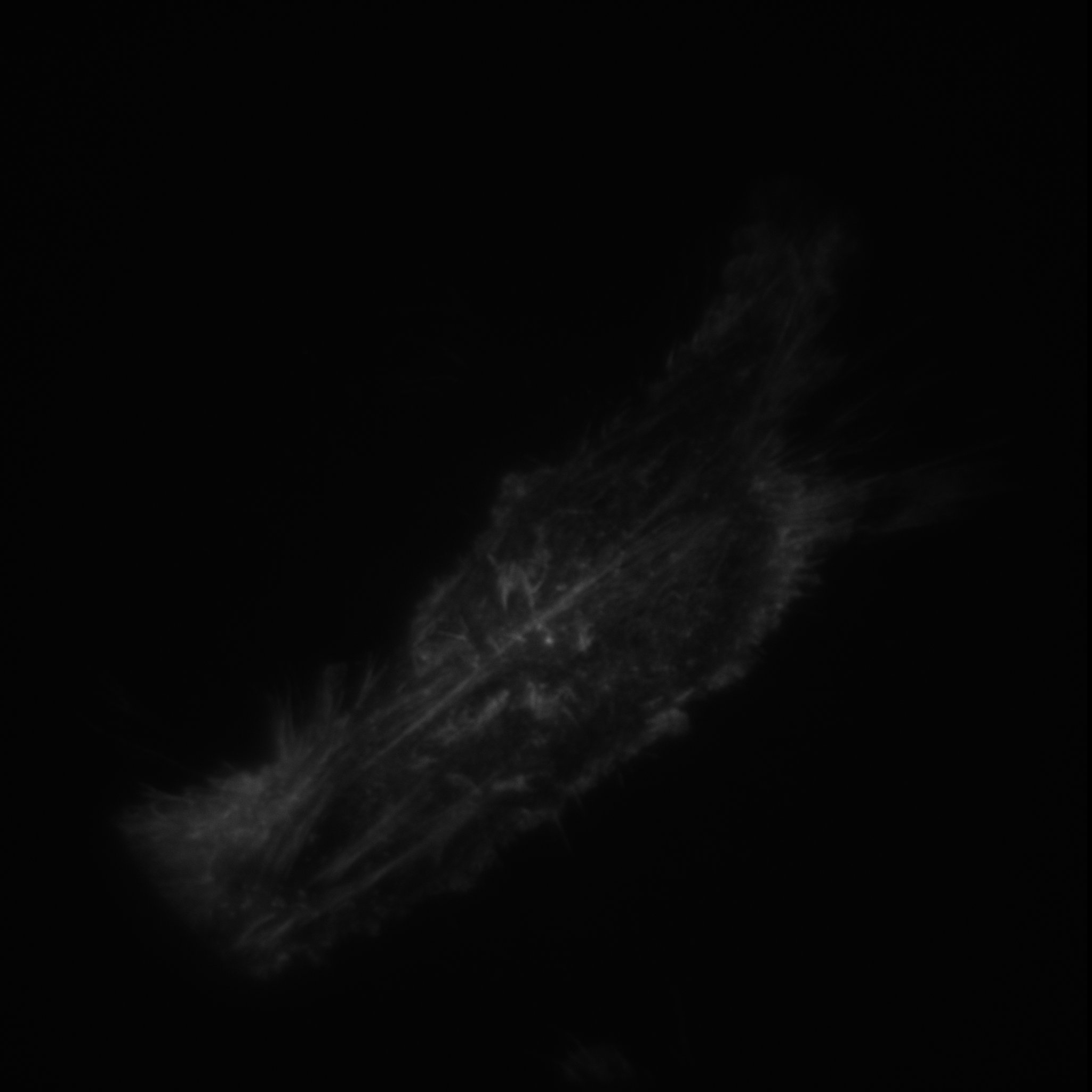

Supplement: Supplementary file 27 — Figure Source Data for Appendix Fig. S7 [file 44319_2026_804_MOESM27_ESM.zip › Appendix Fig. S7/S7D/SMIFH2 before.tif]
