# Supplementary material for: Spontaneous membrane protrusion and cell morphogenesis via self-propelled actin filaments
Source: EMBO Rep. 2026 Jun 25;27(14):3964–81. doi: 10.1038/s44319-026-00804-6 (PMC13400641; doi:10.1038/s44319-026-00804-6)
Supplement: Supplementary file 2 — Peer Review File [file 44319_2026_804_MOESM2_ESM.pdf]

EMBO reports

Spontaneous membrane protrusion and cell morphogenesis via self-propelled actin filaments

Kio Yagami, Takunori Minegishi, Kentarou Baba, Shinji Misu, Hiroko Katsuno-Kambe, Kazunori Okano, Yuichi Sakumura, Yoichiroh Hosokawa, and Naoyuki Inagaki

Corresponding author(s): Naoyuki Inagaki (ninagaki@bs.naist.jp)

Review Timeline:

Submission Date:	28th Jul 25
Editorial Decision:	8th Oct 25
Revision Received:	5th Mar 26
Editorial Decision:	21st Apr 26
Revision Received:	30th Apr 26
Accepted:	11th May 26

Editor: Martina Rembold

Transaction Report:

(Note: With the exception of the correction of typographical or spelling errors that could be a source of ambiguity, letters and reports are not edited. Depending on transfer agreements, referee reports obtained elsewhere may or may not be included in this compilation. Referee reports are anonymous unless the Referee chooses to sign their reports.)

Dear Prof. Inagaki

Thank you for the submission of your research manuscript to our journal. I apologize for the delay in handling your manuscript, which was due to an understaffing of the editorial office. We have now received the full set of referee reports that is copied below.

As you will see, the referees acknowledge that the findings are potentially interesting, but I also note that major concerns are being raised regarding the description and identity of SpTAs. Based on the concerns raised it seems that a major revision will be required to substantiate your findings and conclusions. A better description of SpTAs, their molecular composition, and how these can be distinguished from other actin-based structures in the cel will be required, to name a few. I have discussed this further with the referees, and based on the feedback, I would like to give you the chance to revise your manuscript and address the referee concerns (as detailed above and in their reports).

Acceptance of the manuscript will depend on a positive outcome of a second round of review. It is EMBO Reports policy to allow a single round of revision only and acceptance or rejection of the manuscript will therefore depend on the completeness of your responses included in the next, final version of the manuscript.

We realize that it is difficult to revise to a specific deadline. In the interest of protecting the conceptual advance provided by the work, we recommend a revision within 3 months (January 8th). Please discuss the revision progress ahead of this time with the editor if you require more time to complete the revisions.

I am also happy to discuss the revision further via e-mail or a video call, if you wish.

You can either publish the study as a short report or as a full article. For short reports, the revised manuscript should not exceed 27,000 characters (including spaces but excluding materials & methods and references) and 5 main plus 5 expanded view figures. The results and discussion sections must further be combined, which will help to shorten the manuscript text by eliminating some redundancy that is inevitable when discussing the same experiments twice. For a normal article there are no length limitations, but it should have more than 5 main figures and the results and discussion sections must be separate. In both cases, the entire materials and methods must be included in the main manuscript file.

================
IMPORTANT NOTE:

We perform an initial quality control of all revised manuscripts before re-review. Your manuscript will FAIL this control and the handling will be delayed IN CASE the following APPLIES:

1) A data availability section providing access to data deposited in public databases is missing. If you have not deposited any data, please add a sentence to the data availability section that explains that.

2) Your manuscript contains statistics and error bars based on n=2. Please use scatter blots in these cases. No statistics should be calculated if n=2.

When submitting your revised manuscript, please carefully review the instructions that follow below. Failure to include requested items will delay the evaluation of your revision.

================

When submitting your revised manuscript, we will require:

1) a .docx formatted version of the manuscript text (including legends for main figures, EV figures and tables). Please make sure that the changes are highlighted to be clearly visible.

2) individual production quality figure files as .eps, .tif, .jpg (one file per figure).
Please download our Figure Preparation Guidelines (figure preparation pdf) from our Author Guidelines pages https://www.embopress.org/page/journal/14693178/authorguide for more info on how to prepare your figures.

3) a .docx formatted letter INCLUDING the reviewers' reports and your detailed point-by-point responses to their comments. As part of the EMBO Press transparent editorial process, the point-by-point response is part of the Review Process File (RPF), which will be published alongside your paper.

4) a complete author checklist, which you can download from our author guidelines

(<https://www.embopress.org/page/journal/14693178/authorguide>). Please insert information in the checklist that is also reflected in the manuscript. The completed author checklist will also be part of the RPF.

5) Please note that all corresponding authors are required to supply an ORCID ID for their name upon submission of a revised manuscript (<https://orcid.org/>). Please find instructions on how to link your ORCID ID to your account in our manuscript tracking system in our Author guidelines
(<https://www.embopress.org/page/journal/14693178/authorguide#authorshipguidelines>)

6) We replaced Supplementary Information with Expanded View (EV) Figures and Tables that are collapsible/expandable online. A maximum of 5 EV Figures can be typeset. EV Figures should be cited as 'Figure EV1, Figure EV2" etc... in the text and their respective legends should be included in the main text after the legends of regular figures.

- For the figures that you do NOT wish to display as Expanded View figures, they should be bundled together with their legends in a single PDF file called *Appendix*, which should start with a short Table of Content. Appendix figures should be referred to in the main text as: "Appendix Figure S1, Appendix Figure S2" etc. See detailed instructions regarding expanded view here:
<https://www.embopress.org/page/journal/14693178/authorguide#expandedview>

- Additional Tables/Datasets should be labeled and referred to as Table EV1, Dataset EV1, etc. Legends have to be provided in a separate tab in case of .xls files. Alternatively, the legend can be supplied as a separate text file (README) and zipped together with the Table/Dataset file.

7) Before submitting your revision, primary datasets (and computer code, where appropriate) produced in this study need to be deposited in an appropriate public database (see <
https://www.embopress.org/page/journal/14693178/authorguide#dataavailability>).

Please remember to provide a reviewer password if the datasets are not yet public.

The accession numbers and database should be listed in a formal "Data Availability " section (placed after Materials & Method) that follows the model below (see also < https://www.embopress.org/page/journal/14693178/authorguide#dataavailability>). Please note that the Data Availability Section is restricted to new primary data that are part of this study.

Data availability

The datasets (and computer code) produced in this study are available in the following databases:

- RNA-Seq data: Gene Expression Omnibus GSE46843 (https://www.ncbi.nlm.nih.gov/geo/query/acc.cgi?acc=GSE46843)
- [data type]: [name of the resource] [accession number/identifier/doi] ([URL or identifiers.org/DATABASE:ACCESSION])

*** Note - All links should resolve to a page where the data can be accessed. ***

8) At EMBO Press we ask authors to provide source data for the main manuscript figures. You will receive a separate email with instructions for providing source data with your revised manuscript, including how to upload and organize the files.

Additional information on source data and instruction on how to label the files are available
<https://www.embopress.org/page/journal/14693178/authorguide#sourcedata>

9) The journal requires a statement specifying whether or not authors have competing interests (defined as all potential or actual interests that could be perceived to influence the presentation or interpretation of an article). In case of competing interests, this must be specified in your disclosure statement. Further information: https://www.embopress.org/competing-interests

10) Figure legends and data quantification:
The following points must be specified in each figure legend:

- the name of the statistical test used to generate error bars and P values,
- the EXACT p-values,
- the number (n) of independent experiments (please specify technical or biological replicates) underlying each data point,
- the nature of the bars and error bars (s.d., s.e.m.)

- If the data are obtained from n {less than or equal to} 5, show the individual data points in addition to the SD or SEM.
- If the data are obtained from n {less than or equal to} 2, use scatter blots showing the individual data points.

Discussion of statistical methodology can be reported in the materials and methods section, but figure legends should contain a basic description of n, P and the test applied.

See also the guidelines for figure legend preparation:
https://www.embopress.org/page/journal/14693178/authorguide#figureformat

- Please also include scale bars in all microscopy images and define their size in the legend, not the image.

11) Our journal encourages inclusion of *data citations in the reference list* to directly cite datasets that were re-used and obtained from public databases. Data citations in the article text are distinct from normal bibliographical citations and should directly link to the database records from which the data can be accessed. In the main text, data citations are formatted as follows: "Data ref: Smith et al, 2001" or "Data ref: NCBI Sequence Read Archive PRJNA342805, 2017". In the Reference list, data citations must be labeled with "[DATASET]". A data reference must provide the database name, accession number/identifiers and a resolvable link to the landing page from which the data can be accessed at the end of the reference. Further instructions are available at <https://www.embopress.org/page/journal/14693178/authorguide#referencesformat>.

12) All Materials and Methods need to be described in the main text using our 'Structured Methods' format. According to this format, the Methods section includes a Reagents and Tools Table (listing key reagents, experimental models, software and relevant equipment and including their sources and relevant identifiers) followed by a Methods and Protocols section describing the methods, ideally using a step-by-step protocol format. The aim is to facilitate adoption of the methodologies across labs. Please download and fill our Reagents and Tools Table template (.docx), which you can find in our author guidelines: https://www.embopress.org/page/journal/14693178/authorguide#structuredmethods.
When submitting your revised manuscript, please do not include the Reagents and Tools Table in the Methods section of the manuscript but upload it as a separate file choosing the file type "Reagent Table".
An example of a Method paper with Structured Methods can be found here:
https://www.embopress.org/doi/10.15252/msb.20178071.

13) As part of the EMBO publication's Transparent Editorial Process, EMBO Reports publishes online a Review Process File to accompany accepted manuscripts. This File will be published in conjunction with your paper and will include the referee reports, your point-by-point response and all pertinent correspondence relating to the manuscript.

You are able to opt out of this by letting the editorial office know (emboreports@embo.org). If you do opt out, the Review Process File link will point to the following statement: "No Review Process File is available with this article, as the authors have chosen not to make the review process public in this case."

We would also welcome the submission of cover suggestions, or motifs to be used by our Graphics Illustrator in designing a cover.

I look forward to seeing a revised form of your manuscript when it is ready and please let me know if you have questions or comments regarding the revision.

Yours sincerely,

Martina Rembold, PhD
Senior Editor
EMBO reports

==================

Referee #1:

This study by Kio Yagami et al. describe actin structures that they name "self-propelled treadmilling actin filaments" as self-propelled particles that drive protrusion growth and expansion. Overall, this study provide a substantial amount of microscopy data. However, the description of these "structures" is unclear and their proper role compared to known structures in the cell is not described. The authors claim that those structures are translocated by treadmilling but the use of this term is misleading and no clean demonstration of the mode of transport is provided. In general, a lot of statements are made from a annotated few images. However, quantifications are lacking to support the conclusions proposed by the authors.
For this paper to be appropriate for publication for EMBO reports, the authors should significantly strengthen their analysis and revise their conclusions. In particular, the description of those "SpTAs" should be properly done, with molecular actors forming the structures identified, mode of formation and disassembly described and potential role for those structures more clearly defined.

Major comments
1. Notion of treadmilling.
line 62 : the authors write "F-actins have the inherent property to undergo ... treadmilling". Do the authors refer to actin filaments or to actin networks? Treadmilling is well established for single filaments. However, it is a very slow process and given the regulatory proteins in cells, it is unlikely to contribute to physiological functions. For dynamic actin networks it is more accurate to talk about dynamic steady state when size of the network is constant as well as assembly and disassembly rates are balanced. The authors should clarify this statement. More broadly the use of the word treadmilling is misleading in the whole story and does not make sense for the dynamics described by the authors.
2. Definition and characterization of the SpTAs
Are those SpTA single filaments or actin networks? What is the molecular composition of those SpTAs? The authors say that there are some "filopodium-like" and "lamellipodium-like" F-actin assemblies. How the authors distinguish between those two types of assemblies? How those two types of assemblies are different from each other? This should be clearly demonstrated.
Drug treatments:
- for all the drug treatments described (Figure 1G, Figure S2F, Cytochalasin-B, CK666, SMIFH2), images of the cells without or with the drug treatment should be shown to help the readers to interpret correctly the data.
- An interesting readout would also be to measure the length/size of those assemblies with the different treatments
- The authors says that after treatment with CK666 and SMIFH2, the translocation velocity is slower. Why is this the case? They did not demonstrate that the SpTa were either branched or linear networks. So the use of those two inhibitors and the related effects is a bit unclear. At best, they should depolymerizer other structures present in the cell (lamellipodium or stress fibers), therefore increasing the amount of available monomers and therefore increase the translocation velocity if the hypothesis of the authors (i.e. that the translocation is driven by actin polymerization) is true.

Minor comment
1. Monitoring of actin molecules by speckle imaging of HaloTag actin. Very few examples are shown. On the movie, the HaloTag actin does not seem to be localized to the actin structure. The authors should provide more examples for the statement to be more convincing, as well as a validation of the method.
2. Shootin colocalization Figure S2E: the images are not really convincing to prove a colocalization. More examples should be shown as well as a quantification.
3. Figure 3D there is accumulation of F-actin at the corners. But some snapshots do not demonstrate that "arrival of SpTAs increased F-actins at the protrusive areas". In the patterns there is accumulation of F-actin but no proof of the link with the "SpTAs".

Referee #2:

In the manuscript entitled "Spontaneous cell morphogenesis via self-propelled actin filaments", Kio Yagami and co-workers describe how polymerized actin structures, termed Self-propelled Treadmilling Actin filaments (SpTAs) arise randomly in the cytosol, translocate via treadmilling and accumulate at cell projections. These SpTAs appear to mediate and strengthen the formation of protrusive structures such as lamellipodia and filopodia in glioma cells, necessary for cell polarity. The authors utilize several microscopy techniques to characterize these F-actin structures. Based on their observations, the authors created a mathematical model to predict the behavior of SpTAs and their potential role. Finally, experimental results corroborated the predictions of the model. While the concept of SpTAs as regulators of protrusions, cell polarity and motility is intriguing, the manuscript would benefit from a more in-depth characterization of the SpTAs. Several points need to be addressed before this work can be published in EMBO reports.
• The authors analyze the lifetime of SpTAs, how does this compare to the lifetime of non-SpTAs, such as filopodia? How do the authors distinguish between filopodia-like and filopodial structures? A staining for filopodial markers such as Myosin 10, fascin, FMNL2 or VASP could answer that question.
• What would happen to SpTAs if fascin/VASP are depleted? Would this have an effect on the lifetime or number of SpTAs?
• SpTAs appear to be similar in size to microspikes. Could these ventral SpTAs be microspikes that grow inward because of the substrate on which the cells grow on?
• Inhibition of the Arp2/3 complex by CK666 and formins by SMIFH2 reduced the overall velocity of SpTAs. Do these drug treatments inhibit the formation of de novo SpTAs? Is the lifetime affected as well?

Minor:
Figure 1 G: The durations of the drug treatments are missing in the manuscript.
Figure S2B: time points for [1] and [2] are missing.

Referee #3:

This work reports intriguing observations of self-propelled actin filaments along the ventral and dorsal surface of cells and within the cytoplasm. and shows by simulations and experiments how these filaments respond to cell shape and appear to initiate membrane protrusions. The work raises some questions that need clarification.
Specific comments:
- the title ('cell morphogenesis') is somewhat misleading because it is suggestive of global cell shape changes, whereas the phenomenon reported here is local formation of protrusions. I therefore recommend to make the title clearer and more focused.
- the authors should clarify what is new compared to their earlier study (ref 25 from 2015) that already reported the phenomenon of self-propelled actin filaments
- the authors should clarify the distinction between what they call self-propelled actin filaments versus actin waves. Do these two phenomena co-exist, are they mutually exclusive, can they be clearly distinguished (and how?), and what conditions favor one or the other?
- it is very unclear how actin filaments can move across the cell ventral surface given the crowded nature of the actin cortex and connectivity of actin (via crosslinking proteins) to other actin filaments and other cytoskeletal filaments such as intermediate filaments. Do the authors see any evidence that actin filaments are hampered by being connected to other structures? And if not, how do they explain this?
- The authors distinguish between filopodium-like and lamellipodial-like F-actin assemblies in their data analysis (e.g. Fig 1) but fail to give clear classification criteria. They should explain the criteria in detail.
- The authors observe length-wise motion but also lateral motion. How do they explain the lateral motion along the membrane?
- The authors claim that they observe F-actin also treadmilling in the cytoplasm (Fig 1D), but it is unclear how "self-propelled motion" can be distinguished from diffusion. Presumably both effects are at play. The authors should clarify how they distinguish the sources of motion.
- In the Discussion the authors claim that collisions of actin filaments with the boundary provides a distinct mechanism from actin accumulation via curvature-sensitive actin nucleation, but it is not clear whether the two phenomena can be unambiguously disentangled. The authors should expand on this point.
- For quantitative data, the authors should clarify the statistics: how many biological replicates, how many cells per experiment, how many actin filaments were tracked per cell, etc (e.g. Fig 1G,H; Fig G, I)

Responses to the comments of Referee #1
(Manuscript Number: EMBOR-2025-62421V1).

We thank Referee #1 for the thorough review and for providing constructive suggestions regarding the unclear parts of the paper. We have carefully examined your comments, and addressed all of them point-by-point by adding additional data and explanations. Since new data have been added to this version, the number of Figures has increased from 4 to 6. The following are our point-by-point responses to the comments. Changes in the text of the manuscript are highlighted in red font.

Major comments
1) Notion of treadmilling.
line 62 : the authors write "F-actins have the inherent property to undergo ... treadmilling". Do the authors refer to actin filaments or to actin networks?

Response 1-1: In our original text, “F-actins” refer to single actin filaments. So, this statement aligns with your notion below. We also used the term “F-actin assemblies” to refer to F-actin bundles and networks in cells, not single filaments.

Treadmilling is well established for single filaments. However, it is a very slow process and given the regulatory proteins in cells, it is unlikely to contribute to physiological functions. For dynamic actin networks it is more accurate to talk about dynamic steady state when size of the network is constant as well as assembly and disassembly rates are balanced. The authors should clarify this statement. More broadly the use of the word treadmilling is misleading in the whole story and does not make sense for the dynamics described by the authors.

Response 1-2: In the original text, we explained that SpTA are F-actin “assemblies” (lines 73-75), namely F-actin “bundles and networks”.

Our notion is that single actin filaments have the inherent property to undergo treadmilling, which enables F-actin assemblies to polymerize at the plus ends and disassemble at the minus ends in cells, accompanied by ATP hydrolysis within the filaments. Actin-binding proteins further control the organization and assembly/disassembly of cellular F-actin assemblies. We hope that the reviewer agrees that treadmilling underlies the polymerization at the plus end and disassembly at the minus end of F-actins in SpTAs.

As the reviewer pointed out, the phrase such as “actin filaments undergo directional movement driven by treadmilling” would be misleading. So, we changed this to “actin filament assemblies undergo directional movement driven by their directional assembly and disassembly” (lines 22-23) and corrected similar phrases throughout the revised version. In addition, we explained that “Treadmilling is an inherent property of actin dynamics that allows for the directional assembly and disassembly of F-actin arrays” (lines 297-298).

2) Definition and characterization of the SpTAs
Are those SpTA single filaments or actin networks?

Response 2-1: As explained in **Response 1-2**, SpTAs are actin bundles and networks. As The localization of the F-actin bundling protein fascin in SpTAs (Appendix Fig. S1D) supports this notion. To clarify this point, we indicated the F-actin in Fig. 6A as "F-actin in SpTA" in the revised version.

What is the molecular composition of those SpTAs? The authors say that there are some "filopodium-like" and "lamellipodium-like" F-actin assemblies. How the authors distinguish between those two types of assemblies? How those two types of assemblies are different from each other? This should be clearly demonstrated.

Response 2-2: We thank the referee for this comment. Filopodium-type SpTAs represent self-propelling <u>linear F-actin bundles</u>, while lamellipodium-type SpTAs are self-propelling <u>F-actin meshworks</u>. This has been defined in the revised text (lines 204-206).

In addition, we performed live cell imaging of Lifeact-mCherry (to label SpTA) and mNeonGreen-Fascin (to label fascin) in the revised version. As shown in Appendix Fig. S1D, the filopodial constituent fascin is localized in filopodium-type SpTAs (arrowheads). These data demonstrate molecular component similarities between the of filopodium-type SpTAs and filopodia. Furthermore, the arrival of filopodium-type and lamellipodium-type SpTAs at the cell periphery pushes the membrane to form filopodia (Fig. 3A) and lamellipodia (Fig. 3B), respectively. These data suggest that filopodium-type and lamellipodium-type SpTAs serve as precursors of filopodia and lamellipodia. We also described this in the revised text (lines 206-208).

Drug treatments:
- for all the drug treatments described (Figure 1G, Figure S2F, Cytochalasin-B, CK666, SMIFH2), images of the cells without or with the drug treatment should be shown to help the readers to interpret correctly the data.

Response 2-3: In response to the suggestion, we have shown the images of the cells without or with the drug treatment in the revised version (Appendix Fig. S7D).

In this study, we analyzed the effects of these drugs on filopodium-type SpTAs. We focused on their analyses since the majority of SpTAs are filopodium-type and the filopodium-type and lamellipodium-type are interchangeable. Additionally, the shape of lamellipodium-type SpTAs changes dynamically (arrows, Figs. 1B and Appendix Fig. S1C), making it difficult to analyze their velocity and size. We explained this in the revised version (lines 140-144), and have also shown the images of SpTAs without or with the drug treatment (Cytochalasin B, CK666, SMIFH2) (Fig. 2D and Appendix Fig. S3B).

- An interesting readout would also be to measure the length/size of those assemblies with the different treatments

Response 2-4: In response to the comment, we measured the length of SpTAs in the cells without or with the drug treatment (Cytochalasin B, CK666, SMIFH2) in the revised version. The SpTA length was significantly decreased by the treatment with Cytochalasin B, CK666, or SMIFH2 (Appendix Fig. S3C). We have described these data in the revised version (lines 154-156).

- The authors says that after treatment with CK666 and SMIFH2, the translocation velocity is slower. Why is this the case? They did not demonstrate that the SpTa were either branched or linear networks. So the use of those two inhibitors and the related effects is a bit unclear. At best, they should depolymerizer other structures present in the cell (lamellipodium or stress fibers), therefore increasing the amount of available monomers and therefore increase the translocation velocity if the hypothesis of the authors (i.e. that the translocation is driven by actin polymerization) is true.

Response 2-5: As the reviewer notes, the formin inhibitor SMIFH2 reduced the translocation velocity of filopodium-type SpTAs. Similar effects were observed with VASP knockdown and Cytochalasin B treatment (Fig. 2D and E). Since these treatments inhibit actin polymerization at the plus end, these results support our model that SpTA translocates forward by actin polymerization at the front and disassembly at the rear (Fig. 6A).

We agree that inhibition of actin polymerization by SMFH2 increases the amount of available monomers. However, it would not increase the SpTA translocation velocity because actin polymerization at the front is inhibited by SMFH2.

Interestingly, the Arp2/3 complex inhibitor CK666 also reduced the translocation velocity of filopodium-type SpTAs (Appendix Fig. S3B) and decreased SpTA length (see **Response 2-4**). Although it is unclear how CK666 reduces the translocation velocity of filopodium-type SpTA, consistent with our notion that filopodium-type and SpTAs serve as precursors of filopodia (**Response 2-2**), previous studies reported that CK666 and ARP2/3 knockdown also inhibit filopodia length (He *et al*., *Sci. Rep.* 7, 13685, 2017; Sepúlveda-Ramírez *et al*., *Dev. Biol.* 437, 140–151, 2018). We described these data and discussion in the revised version (Appendix Results and Discussion 3).

Minor comments

1) Monitoring of actin molecules by speckle imaging of HaloTag actin. Very few examples are shown. On the movie, the HaloTag actin does not seem to be localized to the actin structure. The authors should provide more examples for the statement to be more convincing, as well as a validation of the method.

Response 3: Figure 1A for Reviewer #1 shows another example of the speckle imaging data. A filopodium-type SpTA was labelled by EGFP-LifeAct (green). SpTA moved forward (green line). HaloTag actin molecules expressed at a low level were incorporated

at the front of the SpTA (magenta circles), by polymerization, and underwent retrograde flow (magenta lines). These images demonstrate that SpTA moves forward by actin polymerization at the front and undergoes retrograde flow (Figure 1B for Reviewer #1).

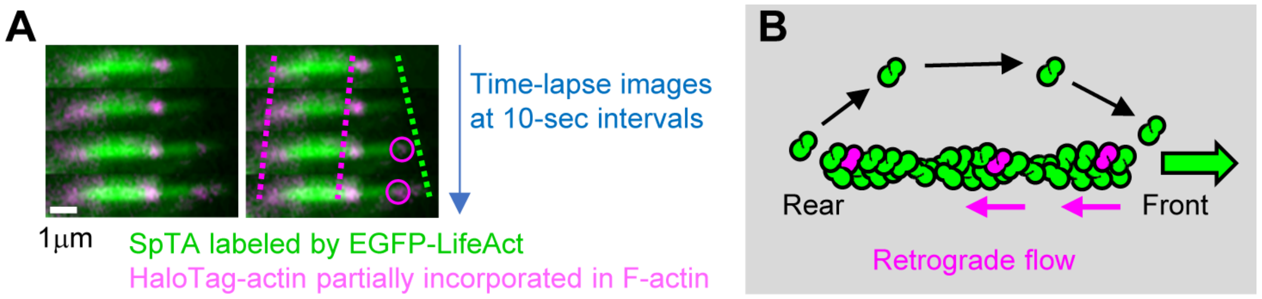

Figure 1 for Reviewer #1. (A) Fluorescence time-lapse images of filopodium-type SpTA in a U251 cell expressing LifeAct-mCherry (green) and HaloTag actin (magenta). The images are identical with (right) and without (left) labelling. (B) A diagram describing actin polymerization at the front of SpTA (green arrow) and the retrograde flow of HaloTag actin (magenta arrow) incorporated in SpTA.

We added this example to the revised version (Appendix Fig. S2A). The revised version shows a total of five examples of similar speckle imaging data (Fig. 2A, Appendix Fig. S2A, S2B, and S2C), thereby supporting our conclusion (Figure 1B for Reviewer #1) and validating the method.

2) Shootin colocalization Figure S2E: the images are not really convincing to prove a colocalization. More examples should be shown as well as a quantification.

Response 4: In addition to the shootin1b colocalization data in Fig. S2E (namely, revised Fig. S2C), we added two examples of the shootin1b colocalization data in the revised version (Appendix Fig. S2E). We also quantified the colocalization of shootin1b and phalloidin in 13 regions using Pearson's coefficient correlation (Appendix Fig. S2F). The Pearson's coefficient correlation exceeded 0.5 (0.59 ± 0.04), indicating colocalization of shootin1b with actin filaments in SpTAs. We described these data in the revised text (lines 147-148).

3) Figure 3D there is accumulation of F-actin at the corners. But some snapshots do not demonstrate that "arrival of SpTAs increased F-actins at the protrusive areas". In the patterns there is accumulation of F-actin but no proof of the link with the "SpTAs".

Response 5: In Fig. 4D (Fig. 3D of the original version), the arrival of the SpTA (yellow arrowheads) increased F-actins at the protrusive area at 65 min. We wonder arrival of which SpTA in Fig. 4D did not increase F-actins at the protrusive areas?

SpTAs appear, move, and then disappear; their average lifetime is 358 ± 28 sec (Appendix Fig. S5C). Therefore, some SpTAs disappear before reaching protrusive areas. Nevertheless, our modeling data, which includes the SpTA lifetime, and experimental data demonstrate that SpTAs accumulate at the protrusive areas (Fig. 4C, E, F and G). Furthermore, inhibition of SpTA translocation by shootin1b KO disrupts actin accumulation at these areas (Fig. 4H and I). These data support our observation that "arrival of SpTAs increased F-actins at the protrusive areas".

Responses to the comments of Referee #2
(Manuscript Number: EMBOR-2025-62421V1).

We thank Referee #2 for the kind review and for suggesting in-depth characterizations of the SpTAs. We have carefully examined your comments, and addressed all of them point-by-point by adding additional data and explanations. Since new data have been added to this version, the number of Figures has increased from 4 to 6. The following are our point-by-point responses to the comments. Changes in the text of the manuscript are highlighted in red font.

Major comments

1) The authors analyze the lifetime of SpTAs, how does this compare to the lifetime of non-SpTAs, such as filopodia?

Response 1-1: In response to this question, we analyzed the lifetime of filopodia. Their lifetime was similar to that of filopodium-type SpTAs (Appendix Fig S5C and J). In addition, the lifetime was also reduced shootin1b by KO (Appendix Fig S5C and J). These finding support our notion that filopodium-type SpTAs are equivalent to the F-actin assemblies in filopodia. We described these data in the revised text (lines 254-257).

How do the authors distinguish between filopodia-like and filopodial structures? A staining for filopodial markers such as Myosin 10, fascin, FMNL2 or VASP could answer that question.

Response 1-2: We thank the referee for pointing out the absence of clear description of these structures. In response to this comment, we performed live cell imaging of U251 cells using Lifeact-mCherry (to label SpTA) and mNeonGreen-Fascin (to label fascin). As shown in Appendix Fig. S1D, the filopodial marker fascin is localized in filopodium-type SpTAs (arrowheads). In addition, the arrival of filopodium-type SpTAs at the cell periphery pushes the membrane to form filopodia (Fig. 3A). Furthermore, the lateral movement of filopodia can be explained by the mechanism of filopodium-type SpTA translocation (Fig. 3E). These data demonstrate the similarity between the filopodium-type SpTAs and filopodia, and suggest that filopodium-type SpTAs serve as precursors of filopodia. We described this in the revised text (lines 204-208).

2) What would happen to SpTAs if fascin/VASP are depleted? Would this have an effect on the lifetime or number of SpTAs?

Response 2: In response to this question, we performed VASP knockdown. VASP knockdown did not significantly change the lifetime or number (de novo formation) of SpTAs (Appendix Fig. S7B and C). However, it decreased their translocation velocity (Fig. 2D and E) and length (Appendix Fig. S3C). Since VASP increases the speed of actin polymerization, these data are consistent with our notion that SpTA translocation is based on actin polymerization. We described these data in the revised version (lines 150-152).

3) SpTAs appear to be similar in size to microspikes. Could these ventral SpTAs be microspikes that grow inward because of the substrate on which the cells grow on?

Response 3: Fascin localized in filopodium-type SpTAs (Appendix Fig. S1D) is also a constituent of microspikes (Vignjevic *et al*, *J Cell Biol* 174, 863–875, 2006), namely F-actin bundles embedded in lamellipodia. Furthermore, the arrival of SpTAs at the cell periphery pushes the membrane to form and expand lamellipodia (Fig. 3B and C). These data suggest that filopodium-type SpTAs also serve as precursors of microspikes. We described this in the revised text (Appendix Results and Discussion 4).

4) Inhibition of the Arp2/3 complex by CK666 and formins by SMIFH2 reduced the overall velocity of SpTAs. Do these drug treatments inhibit the formation of de novo SpTAs? Is the lifetime affected as well?

Response 4: In response to these questions, we analyzed the de novo formation and lifetime of SpTAs. As shown in Appendix Fig. S7B and C, we could not observe their significant change by CK666 or SMIFH2 treatment. These data are described in the revised version (Appendix Results and Discussion 2).

Minor:

Figure 1 G: The durations of the drug treatments are missing in the manuscript.

Response 5: We thank the referee for pointing out this. We analyzed the SpTA velocity before and 10-30 minutes after the drug administration. This has been described in the revised text (lines 873-879).

Figure S2B: time points for [1] and [2] are missing.

Response 6: We thank the reviewer for this comment on Fig. S2B (revised Appendix Fig. S2C). We have indicated that the intervals of the time-lapse montages are 10 seconds in the revised figure legend.

Responses to the comments of Referee #3
(Manuscript Number: EMBOR-2025-62421V1).
We thank Referee #3 for providing constructive suggestions regarding the unclear parts of the paper. We have carefully examined your comments, and addressed all of them point-by-point. Since new data have been added to this version, the number of Figures has increased from 4 to 6. The following are our point-by-point responses to the comments. Changes in the text of the manuscript are highlighted in red font.

Specific comments:
1) the title ('cell morphogenesis') is somewhat misleading because it is suggestive of global cell shape changes, whereas the phenomenon reported here is local formation of protrusions. I therefore recommend to make the title clearer and more focused.
Response 1: We thank the referee for the suggestion to clarify the title. This paper reports that SpTA not only drives local cell protrusions but also global spontaneous cell polarization (Fig. 5) for motility (Appendix Fig. S6D). The translocation of SpTAs into the protrusion results in their lateral decrease (yellow arrows, Fig. 6C). Thus, the self-propelled motion of SpTAs suppresses lateral protrusive activities for polarization (lines 374-376). Nevertheless, as pointed out, the formation of protrusions is a central phenomenon described in this paper. We changed the original title "Spontaneous cell morphogenesis via self-propelled actin filaments" to "Spontaneous cell protrusion and morphogenesis via self-propelled actin filaments" (lines 1-2).

2) the authors should clarify what is new compared to their earlier study (ref 25 from 2015) that already reported the phenomenon of self-propelled actin filaments
Response 2: In the earlier study, we reported that F-actin assemblies translocate along neuronal axons through their directional assembly and disassembly; this is one-dimensional (1D) translocation (Katsuno *et al*, *Cell Rep*, 2015). The present study shows that SpTAs emerge widely in 3D non-neuronal cells and also translocate by directional assembly/disassembly in random directions. A new finding of the present study is that SpTAs move as "self-propelled particles" rather than "waves" (see **Response 3**). Importantly, SpTAs accumulate at protrusive plasma membrane regions in a manner similar to the self-propelled particles. This SpTA accumulation constitutes a positive feedback interaction with the F-actin-driven cell protrusion (red box, 6E), thereby conferring robustness to the protrusions (red arrow) and leading to their further growth (gray arrows, Fig. 6C). We explained this in the revised manuscript (lines 23-30).

3) the authors should clarify the distinction between what they call self-propelled actin filaments versus actin waves. Do these two phenomena co-exist, are they

mutually exclusive, can they be clearly distinguished (and how?), and what conditions favor one or the other?

Response 3: We appreciate the reviewer's feedback regarding this point. As described above, we previously reported F-actin assemblies translocating along axons by their directional assembly/disassembly (Katsuno *et al*, *Cell Rep*, 2015). This F-actin translocation was originally identified by Gary Banker's group and called "waves" (https://onlinelibrary.wiley.com/doi/10.1002/(SICI)1097-4695(199904)39:1%3C97::AID-NEU8%3E3.0.CO;2-Z). Given this historical context, we called them as "actin waves." Because their movement is confined within the narrow axonal shaft, their shape and motion were difficult to analyze in detail in the previous study.

Here, we show F-actin assemblies that translocate by directional assembly/disassembly in non-neuronal cells. These F-actin assemblies are equivalent to the actin wave we reported previously because they both translocate by the same mechanism. The difference is whether they translocate in a 3D environment or in a 1D axonal shaft. Notably, they both move as discrete "particles" rather than propagating uniformly as "waves", and their translocation is not based on the mechanism of the wave propagation. Therefore, it is appropriate to refer to them both as "particles". We discussed this issue in the revised manuscript (lines 318-328).

4) it is very unclear how actin filaments can move across the cell ventral surface given the crowded nature of the actin cortex and connectivity of actin (via crosslinking proteins) to other actin filaments and other cytoskeletal filaments such as intermediate filaments. Do the authors see any evidence that actin filaments are hampered by being connected to other structures? And if not, how do they explain this?

Response 4: Yes, we observed cell ventral surface translocations of SpTA that were hampered by other structures. Please see Figure for Referee #3. In this case, the direction of translocation (arrowheads) was changed by colliding with other actin filaments enriched in the leading edge (arrow). We described these data in the revised version (Appendix Results and Discussion 1, Appendix Fig. S7A, Movie 14).

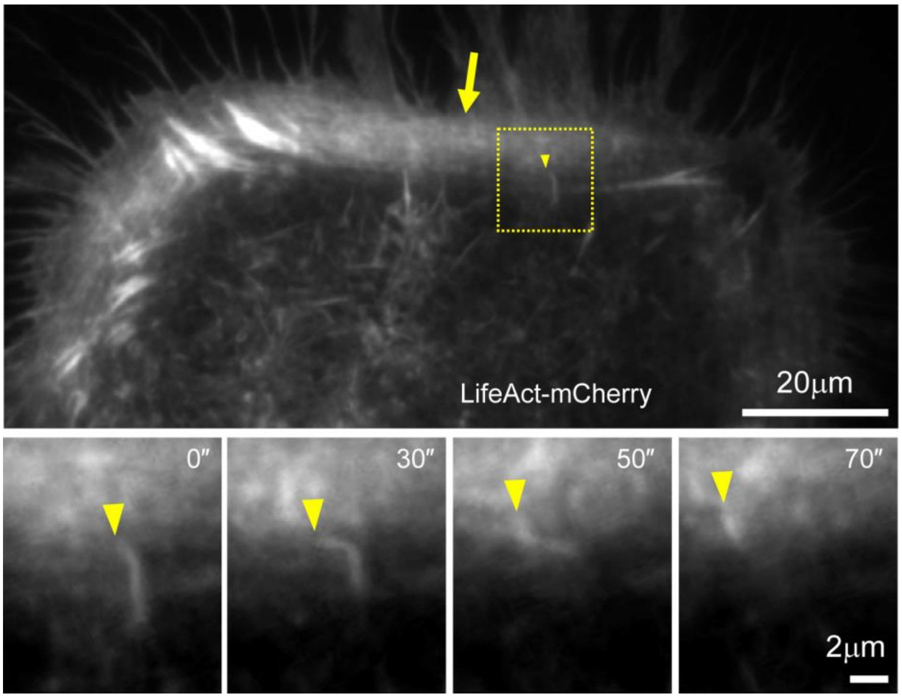

Figure for Reviewer #3. Fluorescence time-lapse images of filopodium-type SpTA in U251 cells expressing LifeAct-mCherry obtained by TIRF microscopy. Lower panels indicate enlarged time-lapse images of the areas indicated by the dashed yellow rectangle.

5) The authors distinguish between filopodium-like and lamellipodial-like F-actin assemblies in their data analysis (e.g. Fig 1) but fail to give clear classification criteria. They should explain the criteria in detail.

Response 5: We thank the referee for pointing out the unclear classification of these structures. Filopodium-type SpTAs represent self-propelling linear F-actin bundles, while lamellipodium-type SpTAs are self-propelling F-actin meshworks. This has been defined in the revised text (lines 204-206).

In addition, we performed live cell imaging of Lifeact-mCherry (to label SpTA) and mNeonGreen-Fascin (to label fascin) in the revised version. As shown in Appendix Fig. S1D, the filopodial constituent fascin is localized in filopodium-type SpTAs (arrowheads). These data demonstrate molecular component similarities between the filopodium-type SpTAs and filopodia. Furthermore, the arrival of filopodium-type and lamellipodium-type SpTAs at the cell periphery pushes the membrane to form filopodia (Fig. 3A) and lamellipodia (Fig. 3B), respectively. These data suggest that filopodium-type and lamellipodium-type SpTAs serve as precursors of filopodia lamellipodia. We also described this in the revised text (lines 206-208).

6) The authors observe length-wise motion but also lateral motion. How do they explain the lateral motion along the membrane?

Response 6: SpTAs undergoing length-wise motion cannot move forward when they arrive at the membrane. However, they can move or slip laterally when lateral obstacles are absent. Since the motion is driven by the length-wise actin polymerization, the force

to propel lateral movement (F_L) is calculated as the cosine θ for the force to propel length-wise motion (F_{LW}), where θ is the angle of the polymerizing F-actins with respect to the membrane (Fig. 3D).

$$F_L = F_{LW} \cos\theta$$

Indeed, as shown in Fig. 3E, the velocity of lateral movement closely matches with the cosine θ for the velocity of the length-wise motion. We included this explanation in the original text (lines 187-199).

7) The authors claim that they observe F-actin also treadmilling in the cytoplasm (Fig 1D), but it is unclear how "self-propelled motion" can be distinguished from diffusion. Presumably both effects are at play. The authors should clarify how they distinguish the sources of motion.

Response 7: As mentioned by the reviewer, diffusion is a core of the translocation mechanism. During SpTA translocation, the local concentration of actin subunits decreases at the front by polymerization and increase at the rear by disassembly, creating a local concentration gradient. The actin subunits diffuse forward along this gradient (magenta arrows, Fig. 6A). Accompanied by this directional diffusion, SpTAs translocate actin. This involvement of diffusion was demonstrated by quantitative mathematical model in our previous paper (Fig. 4 of Katsuno *et al*, https://www.cell.com/cell-reports/fulltext/S2211-1247(15)00674-9). This was explained in the original text (lines 312-316).

8) In the Discussion the authors claim that collisions of actin filaments with the boundary provides a distinct mechanism from actin accumulation via curvature-sensitive actin nucleation, but it is not clear whether the two phenomena can be unambiguously disentangled. The authors should expand on this point.

Response 8: We consider that the present F-actin actin trapping mechanism is distinct from the curvature-sensitive actin nucleation mechanism. The former accumulates F-actins by trapping them at protrusive regions, while the latter accumulates F-actins by promoting F-actin polymerization there. The modeling data of F-actin accumulation (Fig. 4E and F) also support this notion, as our model do not include the curvature-sensitive actin nucleation. The curvature-sensitive actin nucleation is mediated by the curved proteins, so it would sense nanoscopic membrane curvatures. On the other hand, the "trapping" mechanism of SpTA enables F-actin to accumulate in the protrusions of a broader size range, from local protrusions (Fig. 5E) to the global cellular leading edge (Fig. 5F), as well as at different angles (Fig. 4F and G). We discussed this in the revised paper (lines 359-368).

9) For quantitative data, the authors should clarify the statistics: how many biological replicates, how many cells per experiment, how many actin filaments were tracked per cell, etc (e.g. Fig 1G,H; Fig G, I)

Response 9: We thank the reviewer for the careful check of our manuscript. They were included in the revised version (for example lines 873-882).

Dear Prof. Inagaki

Thank you for the submission of your revised manuscript to EMBO reports. I have already sent you a copy of the referee reports (again copied below) and herewith follow up with the official decision letter.

All three referees are very positive about the study and request only minor changes to clarify text and a few aspects of the methodology.

From the editorial side, there are also a few things that we need before we can proceed with the official acceptance of your study.

- The Disclosure and competing interests statement needs to be placed after the Acknowledgments.

- Regarding the Author Contributions, we now use CRediT to specify the contributions of each author in the journal submission system. CRediT replaces the author contribution section, which therefore needs to be removed from the manuscript text. You can use the free text box in our system if you wish to provide more detailed descriptions. See also guide to authors https://www.embopress.org/page/journal/14693178/authorguide#authorshipguidelines.

- Please add a callout for Movie EV14 in the text.

- The Data availability section should exclusively refer to data deposited in public repositories. Therefore, please remove the following statement: "Any additional information required to reanalyze the data reported in this paper is available upon reasonable request."

- You indicated in the Author Checklist, C54, that you included a statement on mycoplasma testing or cell line authentication in the Methods section. I might have missed it but could not find a relevant statement. Could you please double-check?

- Please provide a clean version of the Appendix and upload it as PDF file.

Please address the following points related to the figure legends:
1) Please provide the exact p values in the legends of figures 4E, G, H, I; 5B, E, unless these are smaller than $p<0.0001$.
2) Please define the error bars in the legend of figure 2E.

- Finally, EMBO Reports papers are accompanied online by
A) a short (1-2 sentences) summary of the findings and their significance,
B) 2-3 bullet points highlighting key results and
C) a schematic summary figure that provides a sketch of the major findings (not a data image).
Please provide the summary figure as a separate file in PNG or JPG format at a size of 550x300 pixels (width x height). Please note that the size is rather small and that text needs to be readable at the final size. Please send us this information along with the revised manuscript.

We look forward to seeing a final version of your manuscript as soon as possible.

With kind regards,

Martina Rembold, PhD
Senior Editor
EMBO reports

==================

Referee #1:

I thank the authors for addressing my comments.
My only reservation concerns the classification of SpTAs, and more specifically the way in which the authors distinguish filopodium-type SpTAs from lamellipodium-type SpTAs. Indeed, the difference between these two types of structures has not yet been clearly defined. The authors should provide the objective (non-biased) criteria they use to distinguish between these two types of structures.

Minor point:

In lines 144-147: the authors added this sentence: "To examine whether the observed F-actin assemblies translocate via directional assembly and disassembly, we focused on the F-actin bundles, since they comprise the majority of the F-actin bundles, and since the F-actin bundles meshworks are interchangeable."
I suppose this sentence is meant to explain why the analysis is limited to filopodium-like structures in the presence of drugs. However, it doesn't read very smoothly and is difficult to understand. It should be reworded.

Referee #2:

The authors have done a great job in addressing all my concerns. It is very nice work.

Referee #3:

The authors have satisfactorily addressed the comments. The results remain puzzling and provocative, but are original and are likely to trigger more work. One comment should still be addressed: how do the authors distinguish "SptA" structures that undergo motion due to diffusion from structures undergoing motion due to the claimed self-propelled motion? In their reply the authors mistakenly interpret the question as a question about G-actin diffusion, but the question is about whole-center-of-mass diffusion of the entire SptA structure. Did the authors measure mean square displacements vs time and check power-law exponents to distinguish difffusive vs superdiffusive motion, and did they check for x,y,z (an)isotropy?

Responses to the comments of Referee #1
(Manuscript Number: EMBOR-2025-62421V2).

We thank Referee #1 for the additional careful comments on our paper. The following are our responses to the comments. Changes in the text of the manuscript are highlighted in red font.

Comment 1: My only reservation concerns the classification of SpTAs, and more specifically the way in which the authors distinguish filopodium-type SpTAs from lamellipodium-type SpTAs. Indeed, the difference between these two types of structures has not yet been clearly defined. The authors should provide the objective (non-biased) criteria they use to distinguish between these two types of structures.

Response 1: In the revised version, we clearly defined that filopodium-type SpTAs as linear F-actin bundles that undergo directional movement via directional actin assembly and disassembly (arrowheads, Fig. 1B; Movie EV1 right), while lamellipodium-type SpTAs are F-actin meshworks that undergo directional movement via directional actin assembly and disassembly (arrows, Fig. 1B; Movie EV1 right), indicating their figures and movies (lines 210-212). We think that readers can easily distinguish between the two types.

Comment 2: In lines 144-147: the authors added this sentence: "To examine whether the observed F-actin assemblies translocate via directional assembly and disassembly, we focused on the F-actin bundles, since they comprise the majority of the F-actin bundles, and since the F-actin bundles meshworks are interchangeable."… However, it doesn't read very smoothly and is difficult to understand. It should be reworded.

Response 2: We thank the referee for this comment. The sentence “To examine whether the observed F-actin assemblies translocate via directional assembly and disassembly, we focused on the F-actin bundles, since they comprise the majority of the F-actin bundles, and since the F-actin bundles meshworks are interchangeable” was reworded to:
“To examine whether the observed F-actin assemblies translocate via directional assembly and disassembly, we further analyzed their motion under drug treatments and molecular knockdown. However, the shape of the F-actin meshworks changes dynamically, which makes analyzing their velocity difficult. Additionally, the F-actin bundles comprise the majority of the F-actin assemblies, and the F-actin bundles and meshworks are interchangeable. Therefore, we focused on the analyses of the F-actin bundles.” (lines 142-148).

Responses to the comments of Referee #3
(Manuscript Number: EMBOR-2025-62421V2).

We thank Referee #1 for the additional comment on our paper. We now understood the Referee's comment on diffusion. The following is our response.

Comment: One comment should still be addressed: how do the authors distinguish "SptA" structures that undergo motion due to diffusion from structures undergoing motion due to the claimed self-propelled motion? … Did the authors measure mean square displacements vs time and check power-law exponents to distinguish difffusive vs superdiffusive motion, and did they check for x,y,z (an)isotropy?

Response 1: In response to the question, we traced the trajectories of SpTAs and noted that they were distributed isotropically around their points of origin, with no apparent directional bias (**Figure 1A for Reviewer #3**). Since the SpTA movement data were obtained using TIRF microscopy, which visualizes the region near the ventral plasma membrane, the trajectories are described in two dimensions. We then quantified SpTA dynamics by computing the mean square displacement (MSD) as a function of time lag

Figure for referee with unpublished data and its description has been removed upon request by the authors.

(τ) and extracting the power-law exponent α from log–log fits. The MSD curves display clear power-law scaling (**Figure 1B for Reviewer #3**). The α values of the SpTAs are mainly distributed between the reference lines for diffusive ($\alpha = 1$) and ballistic ($\alpha = 2$) motion (**Figure 1C for Reviewer #3**). This indicates that the SpTA motion is predominantly superdiffusive rather than diffusive.

Any unbound molecular assembly would diffuse to some extent in the cytoplasm. However, ventrally translocating SpTAs are anchored to the adhesive substrates (Fig. 2B left), which humpers their diffusion. Therefore, the isotropic and superdiffusive motions of SpTAs are consistent with the mechanism that drives their motility.

We noticed that the power-law exponent α appears to peak at around 1.5 (**Figure 1C for Reviewer #3**), implying the interesting possibility that there is an optimal α value for the efficient SpTA activity. As the optimal α value of SpTA for cell morphogenesis is beyond the scope of this paper, we will address this in a future study.

2nd Revision - Editorial Decision **11th May 2026**

Prof. Naoyuki Inagaki
Nara Institute of Science and Technology
Division of Biological Science
Takayama-cho 8916-5
Ikoma, Nara 630-0192
Japan

Dear Prof. Inagaki,

I am very pleased to accept your manuscript for publication in the next available issue of EMBO reports. Thank you for your contribution to our journal.

Your manuscript will be processed for publication by EMBO Press. It will be copy edited and you will receive page proofs prior to publication. Please note that you will be contacted by Springer Nature Author Services to complete licensing and payment information.

You may qualify for financial assistance for your publication charges - either via a Springer Nature fully open access agreement or an EMBO initiative. Check your eligibility: https://link.springer.com/journal/44319/how-to-publish-with-us

Should you be planning a Press Release on your article, please get in contact with embo_production@springernature.com as early as possible in order to coordinate publication and release dates.

If you have any questions, please do not hesitate to contact the Editorial Office. Thank you for your contribution to EMBO Reports.

Yours sincerely,

Martina Rembold, PhD
Senior Editor
EMBO reports

>>> Please note that it is EMBO Reports policy for the transcript of the editorial process (containing referee reports and your response letter) to be published as an online supplement to each paper. If you do NOT want this, you will need to inform the Editorial Office via email immediately. More information is available here: https://link.springer.com/partners/embo-press/editorial-policies#Peer%20review

EMBO Press Author Checklist

Corresponding Author Name: Naoyuki Inagaki
Journal Submitted to: EMBO Reports
Manuscript Number: EMBOR-2025-62421V1

USEFUL LINKS FOR COMPLETING THIS FORM
The EMBO Journal - Author Guidelines
EMBO Reports - Author Guidelines
Molecular Systems Biology - Author Guidelines
EMBO Molecular Medicine - Author Guidelines

Reporting Checklist for Life Science Articles (updated January
This checklist is adapted from Materials Design Analysis Reporting (MDAR) Checklist for Authors. MDAR establishes a minimum set of requirements in transparent reporting in the life sciences (see Statement of Task: 10.31222/osf.io/9sm4x). Please follow the journal's guidelines in preparing your
Please note that a copy of this checklist will be published alongside your article.

Abridged guidelines for figures
1. Data
The data shown in figures should satisfy the following conditions:
- the data were obtained and processed according to the field's best practice and are presented to reflect the results of the experiments in an accurate and unbiased manner.
- ideally, figure panels should include only measurements that are directly comparable to each other and obtained with the same assay.
- plots include clearly labeled error bars for independent experiments and sample sizes. Unless justified, error bars should not be shown for technical
- if n<5, the individual data points from each experiment should be plotted. Any statistical test employed should be justified.
- Source Data should be included to report the data underlying figures according to the guidelines set out in the authorship guidelines on Data

2. Captions
Each figure caption should contain the following information, for each panel where they are relevant:
- a specification of the experimental system investigated (eg cell line, species name).
- the assay(s) and method(s) used to carry out the reported observations and measurements.
- an explicit mention of the biological and chemical entity(ies) that are being measured.
- an explicit mention of the biological and chemical entity(ies) that are altered/varied/perturbed in a controlled manner.
- the exact sample size (n) for each experimental group/condition, given as a number, not a range;
- a description of the sample collection allowing the reader to understand whether the samples represent technical or biological replicates (including how many animals, litters, cultures, etc.).
- a statement of how many times the experiment shown was independently replicated in the laboratory.
- definitions of statistical methods and measures:
  - common tests, such as t-test (please specify whether paired vs. unpaired), simple χ2 tests, Wilcoxon and Mann-Whitney tests, can be unambiguously identified by name only, but more complex techniques should be described in the methods section;
  - are tests one-sided or two-sided?
  - are there adjustments for multiple comparisons?
  - exact statistical test results, e.g., P values = x but not P values < x;
  - definition of 'center values' as median or average;
  - definition of error bars as s.d. or s.e.m.

Please complete ALL of the questions below.
Select "Not Applicable" only when the requested information is not relevant for your study.

Materials

Newly Created Materials	Information included in the manuscript?	In which section is the information available? (Reagents and Tools Table, Materials and Methods, Figures, Data Availability Section)
New materials and reagents need to be available; do any restrictions apply?	Yes	Reagents and Tools Table, Methods

Antibodies	Information included in the manuscript?	In which section is the information available? (Reagents and Tools Table, Materials and Methods, Figures, Data Availability Section)
For **antibodies** provide the following information:<br>- Commercial antibodies: RRID (if possible) or supplier name, catalogue number and or/clone number<br>- Non-commercial: RRID or citation	Yes	Reagents and Tools Table, Methods

DNA and RNA sequences	Information included in the manuscript?	In which section is the information available? (Reagents and Tools Table, Materials and Methods, Figures, Data Availability Section)
**Short novel DNA or RNA including primers, probes**: provide the sequences.	Yes	Reagents and Tools Table, Methods

Cell materials	Information included in the manuscript?	In which section is the information available? (Reagents and Tools Table, Materials and Methods, Figures, Data Availability Section)
**Cell lines:** Provide species information, strain. Provide accession number in repository **OR** supplier name, catalog number, clone number, and/**OR** RRID.	Yes	Reagents and Tools Table, Methods
**Primary cultures:** Provide species, strain, sex of origin, genetic modification status.	Not Applicable	
Report if the cell lines were recently **authenticated** (e.g., by STR profiling) and tested for mycoplasma contamination.	Yes	Methods

Experimental animals	Information included in the manuscript?	In which section is the information available? (Reagents and Tools Table, Materials and Methods, Figures, Data Availability Section)
**Laboratory animals or Model organisms:** Provide species, strain, sex, age, genetic modification status. Provide accession number in repository **OR** supplier name, catalog number, clone number, **OR** RRID.	Not Applicable	
**Animal observed in or captured from the field:** Provide species, sex, and age where possible.	Not Applicable	
Please detail **housing and husbandry conditions**.	Not Applicable	

Plants and microbes	Information included in the manuscript?	In which section is the information available? (Reagents and Tools Table, Materials and Methods, Figures, Data Availability Section)
**Plants:** provide species and strain, ecotype and cultivar where relevant, unique accession number if available, and source (including location for collected wild specimens).	Not Applicable	
**Microbes:** provide species and strain, unique accession number if available, and source.	Not Applicable	

Human research participants	Information included in the manuscript?	In which section is the information available? (Reagents and Tools Table, Materials and Methods, Figures, Data Availability Section)
If collected and within the bounds of privacy constraints report on age, sex and gender or ethnicity for all study participants.	Not Applicable	

Core facilities	Information included in the manuscript?	In which section is the information available? (Reagents and Tools Table, Materials and Methods, Figures, Data Availability Section)
If your work benefited from core facilities, was their service mentioned in the acknowledgments section?	Not Applicable	

Design

Study protocol	Information included in the manuscript?	In which section is the information available? (Reagents and Tools Table, Materials and Methods, Figures, Data Availability Section)
If study protocol has been **pre-registered, provide DOI in the manuscript**. For clinical trials, provide the trial registration number **OR** cite DOI.	Not Applicable	
Report the **clinical trial registration number** (at ClinicalTrials.gov or equivalent), where applicable.	Not Applicable	

Laboratory protocol	Information included in the manuscript?	In which section is the information available? (Reagents and Tools Table, Materials and Methods, Figures, Data Availability Section)
Provide DOI OR other citation details if **external detailed step-by-step protocols** are available.	Not Applicable	

Experimental study design and statistics	Information included in the manuscript?	In which section is the information available? (Reagents and Tools Table, Materials and Methods, Figures, Data Availability Section)
Include a statement about **sample size** estimate even if no statistical methods were used.	Yes	Figure legends
Were any steps taken to minimize the effects of subjective bias when allocating animals/samples to treatment (e.g. **randomization procedure**)? If yes, have they been described?	Yes	Methods
Include a statement about **blinding** even if no blinding was done.	Yes	Methods
Describe **inclusion/exclusion criteria** if samples or animals were excluded from the analysis. Were the criteria pre-established?<br><br>If sample or data points were omitted from analysis, report if this was due to attrition or intentional exclusion and provide justification.	Not Applicable	
For every figure, are **statistical tests** justified as appropriate? Do the data meet the assumptions of the tests (e.g., normal distribution)? Describe any methods used to assess it. Is there an estimate of variation within each group of data? Is the variance similar between the groups that are being statistically compared?	Yes	Methods, Figure legends, Statistical source data

Sample definition and in-laboratory replication	Information included in the manuscript?	In which section is the information available? (Reagents and Tools Table, Materials and Methods, Figures, Data Availability Section)
In the figure legends: state number of times the experiment was **replicated** in laboratory.	Yes	Methods, Figure legends
In the figure legends: define whether data describe **technical or biological replicates**.	Yes	Figure legends

Ethics

Ethics	Information included in the manuscript?	In which section is the information available? (Reagents and Tools Table, Materials and Methods, Figures, Data Availability Section)
Studies involving **human participants**: State details of **authority granting ethics approval** (IRB or equivalent committee(s), provide reference number for approval.	Not Applicable	
Studies involving **human participants**: Include a statement confirming that **informed consent** was obtained from all subjects and that the experiments conformed to the principles set out in the WMA Declaration of Helsinki and the Department of Health and Human Services Belmont Report.	Not Applicable	
Studies involving **human participants:** For publication of **patient photos**, include a statement confirming that consent to publish was obtained.	Not Applicable	
Studies involving experimental **animals**: State details of **authority granting ethics approval** (IRB or equivalent committee(s), provide reference number for approval. Include a statement of compliance with ethical regulations.	Not Applicable	
Studies involving **specimen and field samples:** State if relevant **permits** obtained, provide details of authority approving study; if none were required, explain why.	Not Applicable	

Dual Use Research of Concern (DURC)	Information included in the manuscript?	In which section is the information available? (Reagents and Tools Table, Materials and Methods, Figures, Data Availability Section)
Could your study fall under dual use research restrictions? Please check biosecurity documents and list of **select agents and toxins** (CDC): https://www.selectagents.gov/sat/list.htm	Not Applicable	
If you used a select agent, is the security level of the lab appropriate and reported in the manuscript?	Not Applicable	
If a study is subject to dual use research of concern regulations, is the name of the **authority granting approval and reference number** for the regulatory approval provided in the manuscript?	Not Applicable	

Reporting

The MDAR framework recommends adoption of discipline-specific guidelines, established and endorsed through community initiatives. Journals have their own policy about requiring specific guidelines and recommendations to complement MDAR.

Adherence to community standards	Information included in the manuscript?	In which section is the information available? (Reagents and Tools Table, Materials and Methods, Figures, Data Availability Section)
State if relevant guidelines or checklists (e.g., **ICMJE, MIBBI, ARRIVE, PRISMA**) have been followed or provided.	Not Applicable	
For **tumor marker prognostic studies,** we recommend that you follow the **REMARK** reporting guidelines (see link list at top right). See author guidelines, under 'Reporting Guidelines'. Please confirm you have followed these guidelines.	Not Applicable	
For **phase II and III randomized controlled trials**, please refer to the **CONSORT** flow diagram (see link list at top right) and submit the CONSORT checklist (see link list at top right) with your submission. See author guidelines, under 'Reporting Guidelines'. Please confirm you have submitted this list.	Not Applicable	

Data Availability

Data availability	Information included in the manuscript?	In which section is the information available? (Reagents and Tools Table, Materials and Methods, Figures, Data Availability Section)
Have **primary datasets** been deposited according to the journal's guidelines (see 'Data Deposition' section) and the respective accession numbers provided in the Data Availability Section?	Yes	Reagents and Tools Table, Methods
Were **human clinical and genomic datasets** deposited in a public access-controlled repository in accordance to ethical obligations to the patients and to the applicable consent agreement?	Not Applicable	
Are **computational models** that are central and integral to a study available without restrictions in a machine-readable form? Were the relevant accession numbers or links provided?	Yes	Reagents and Tools Table, Methods
If publicly available data were reused, provide the respective **data citations in the reference list**.	Not Applicable	
